# An Efficient Approach to Phosphorylated Isoindoline Fused with Triazoles via Zn-Catalyzed Cascade Cyclization of 2–Propynol Benzyl Azides and Diarylphosphine Oxides

**DOI:** 10.3390/molecules24193526

**Published:** 2019-09-29

**Authors:** Tao Yang, Xian-Rong Song, Ruchun Yang, Haixin Ding, Jiang Bai, Qiang Xiao

**Affiliations:** Key Laboratory of Organic Chemistry, Institute of Organic Chemistry, Jiangxi Science & Technology Normal University, Nanchang 330013, Jiangxi, China; tao_yang2019@yeah.net (T.Y.); ouyangruchun@yeah.net (R.Y.); dinghaixin0204@yeah.net (H.D.); mtbaijiang@yeah.net (J.B.)

**Keywords:** Zn-catalyzed, cascade cyclization, propargylic alcohols, azides, phosphorylated isoindolines, triazoles

## Abstract

An efficient approach for the synthesis of phosphorylated isoindoline fused with triazoles via Zn(OTf)_2_-catalyzed cascade cyclization of easily prepared ortho–propynol benzyl azides and diarylphosphine oxides is developed. The transformation occurred smoothly in moderate to excellent yields and tolerated various propargylic alcohol substrates.

## 1. Introduction

**Figure 1:**
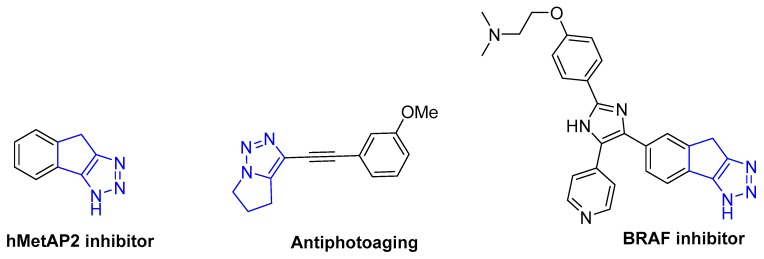
Representative molecules of bioactive fused 1,2,3–triazoles.

Isoindolines are important scaffolds that are widely found in various drugs, bioactive compounds, and natural products [1,2]. On the other hand, fused 1,2,3–triazoles are also interesting skeletons found to exhibit a wide range of biological activities. Figure 1 shows the representative examples of some bioactive heterocyclic compounds fused with triazoles. In particular, isoindolines fused with 1,2,3–triazoles have attracted significant attention due to their distinct synthetic applications and pharmacological characteristics [3,4,5,6,7]. In the past several decades, some conventional approaches for construction of such compounds have been developed [8,9,10,11,12,13,14,15,16,17,18,19,20,21,22,23,24,25,26,27,28]. These approaches mainly include: (1) the reaction of azide and alkyne functionalities via intramolecular cycloaddition [9]; (2) Metal-catalyzed cyclization of terminal alkynes and azidoaryl halides [10,11,12,13]; and (3) Organocatalyzed the cyclization of organic azides and carbonyl compounds [14,15,16,17,18,19,20,21,22,23,24,25,26,27,28]. Despite these advances, new approaches for the construction of diverse functionalized isoindolines fused with triazoles under mild conditions is still attractive and desirable.

Meanwhile, organophosphorus compounds have been widely applied in material science and organic and pharmaceutical chemistry [29,30,31]. It is well established that the introduction of organophosphorus functionalities into heterocycles could potentially introduce new biological and physical properties into the parent molecule [32,33,34]. If both the isoindoline–triazole and phosphinoyl group can be simultaneously installed in organic frameworks, such compounds could be used to modulate the bioactivity of original pharmaceutical molecule or lead compounds. Therefore, it is important to develop an efficient strategy for the one-pot construction isoindolines fused with triazoles and C–P bond.

**Scheme 1:**
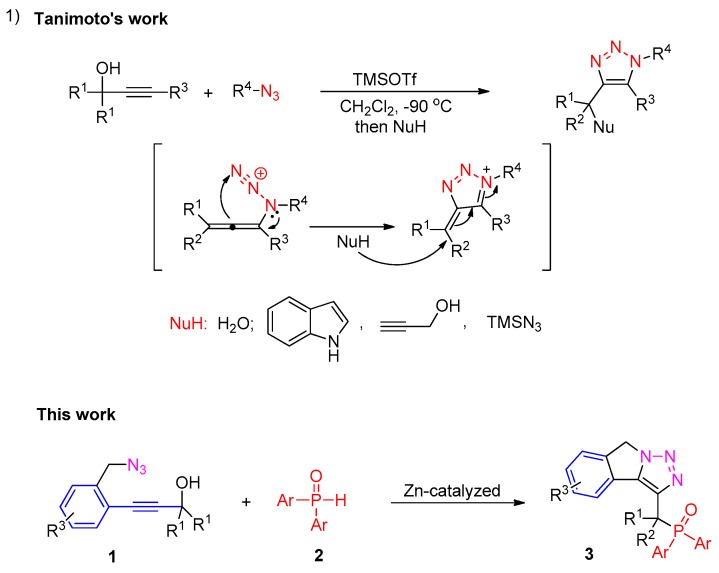
Previous work and our strategy.

In recent years, tandem reactions of propynols with nucleophiles have emerged as powerful tools for the construction of various carbo- and heterocycle compounds [35,36,37,38]. In 2013, the Tanimoto group developed an efficient approach to prepare fully substituted 1H–1,2,3–triazoles through TMSOTf-promoted cyclization of alkynols with organic azides [39,40]. Mechanistically, the reaction takes place by the nucleophilic attack of the organic azides to the allenyl cation intermediate, followed by another nucleophilic attack on intermediate **II**. In their creative work, a series of nucleophilies, including indoles, azides, alcohols, amines, etc., were successfully added to intermediate **II**. Notably, phosphine nucleophiles have not been reported to capture intermediate **II**. In a continuation of our work on propargylic alcohols [41,42,43,44,45,46] and the synthesis of organophosphorus compounds, we herein report our detailed findings (Scheme 1).

## 2. Results and Discussion

To begin, the reaction between 2–propynol benzyl azides **1a** and diphenylphosphine oxide **2a** was selected as the mock-up reaction to examine the optimal conditions, as shown in Table 1. In an initial experiment, the reaction was performed in DCE at 100 ºC catalyzed by Sc (OTf)_3_ under open air (entry 1). Gratifyingly, our expected product ((8H–[1,2,3]triazolo[5,1–a]isoindol–3–yl)diphenylmethyl)diphenylphosphine oxide **3a** was isolated in 42% yield after 1.0 h. The **3a** was unambiguously confirmed by X-ray structure analysis [47]. On the basis of this result, a subsequent brief screening of some representative Lewis acid, including Zn(OTf)_2_, Cu(OAc)_2_, Cu(OTf)_2_, CuCl_2_, and AgOTf revealed that Zn(OTf)_2_ performed most efficiently and could increase the yield of desired product **3a** to 53% (entries 2−6). Subsequently, among the reaction solvents examined, it turned out that the reaction in CH_3_CN gave the best yields (entries 7–10). No better results were obtained when decreasing or increasing the reaction temperature (entries 11–12). Then, the loading of catalyst and diphenylphosphine oxide **2a** was also investigated, and the results indicated that 20 mol% of Zn(OTf)_2_ and 2.5 equiv of **2a** gave the better yield for this transformation (entries 13–16). Therefore, we selected the following optimum conditions: 0.1 mmol of 2–propynol benzyl azides **1**, 20 mol% of Zn(OTf)_2_, 2.5 equiv of diphenylphosphine oxide **2a** in CH_3_CN at 100 °C for 1.0 h.

**Figure 2:**
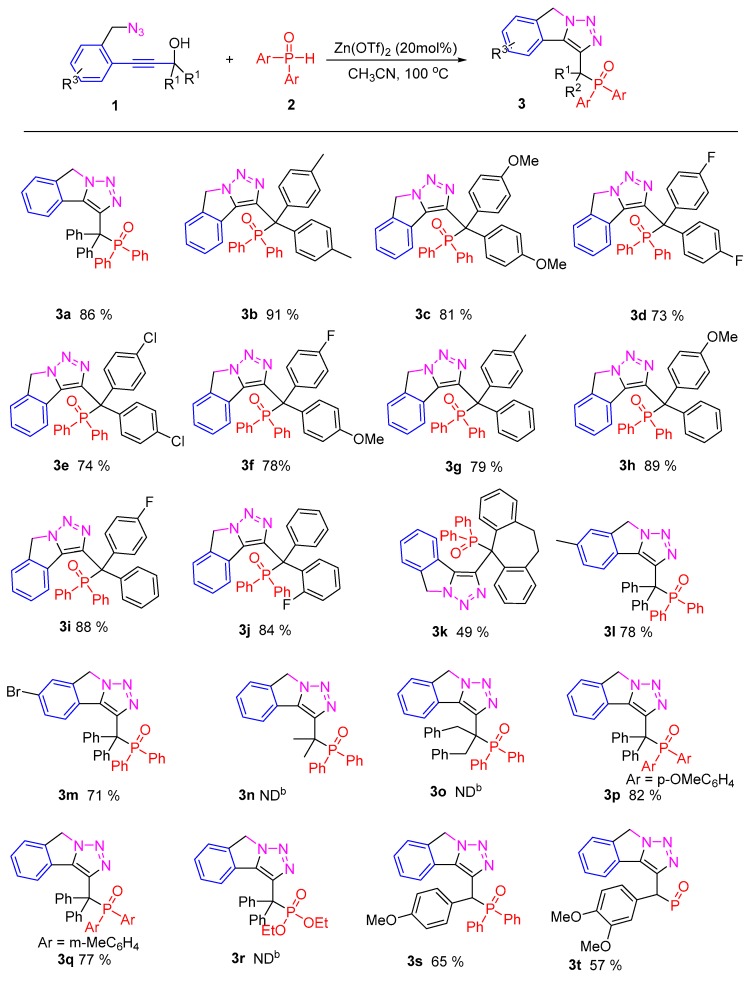
Transformation of propargylic alcohols **1** to phosphorylated isoindoine fused with trizoles **3**. ^b^ The corresponding product cannot be detected by TLC.

With optimal reaction conditions for the transformation of 2–propynol benzyl azides to P-containing isoindoline fused with triazoles in hand, we investigated the generality of the cascade reaction, and the corresponding results are summarized in Figure 2. The phosphorylated isoindoline fused with triazoles **3** were formed in moderate to excellent yields under optimal conditions. Both electron-donating (Me, OMe) and electron-withdrawing groups (F, Cl) on two aromatic rings were tolerated well to generate the target compounds in acceptable yields. In general, substrates containing electron-poor substituents gave lower yields than electron-rich ones (**3a–3c** vs **3d–3e**). Furthermore, the steric effect of substituents showed little effect on this transformation; substrate bearing the ortho-position substituents on aryl groups generates a good yield of desired product (**3j**). Notably, the substrate with polycyclic aryl ring still gave a moderate yield of 49% (**3k**), which might be due to the ring strain. Moreover, no desired products (**3n** and **3o**) were observed using substrates with dibenzyl and dimethyl instead of aryl groups under the optimal conditions. This might be due to the fact that it is difficult to the formation of allenic intermediate. Subsequently, the substituents (R^2^) on another benzene ring were also examined under the optimal conditions. Both electron-donating and withdrawing substituents on the para-position of aryl ring were found to be compatible. Additionally, diarylphosphine oxides with representative substituents at the 3- and 4-positions (**1p**–**1q**) were tolerated well for this reaction (**3p**–**3q**). However, no desired product was obtained when diethyl phosphite **1r** was employed under the optimal conditions. This might be due to the low nucleophilicity and thermostability of the diethyl phosphite. The final concern was that the reaction system still worked smoothly with secondary propynols; the corresponding products (**3s**–**3t**) were obtained in moderate yield. Compared to tertiary propynols substrates, lower yields were obtained because of the formation of unstable intermediate **B**.

**Scheme 2:**
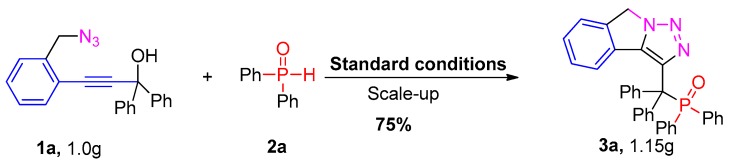
Scale-up experiment.

Furthermore, a gram-scale reaction of ortho–propynol benzyl azides **1a** and diphenylphosphine oxide **2a** could be conducted smoothly to demonstrate the scalability of this reaction under the standard conditions (Scheme 2). The target compound phosphorylated isoindoline **3a** was generated in 75% yield, which may offer potential application in organic synthesis and medicinal chemistry.

**Scheme 3:**
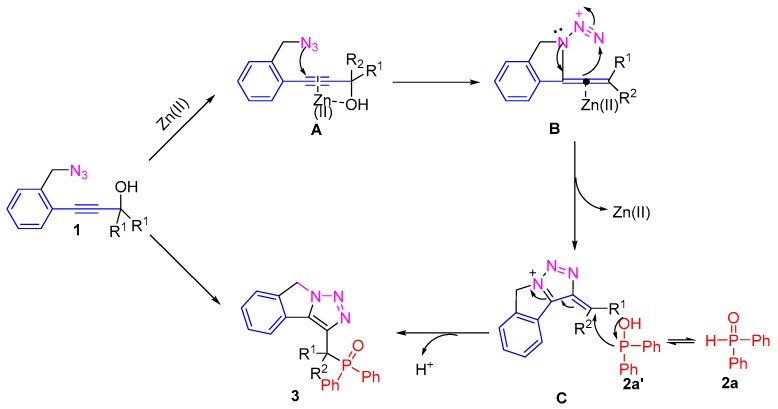
Proposed reaction mechanism.

According to our experimental results and reported literatures [39,40,48,49], a plausible reaction mechanism was proposed in Scheme 3. Firstly, the coordination of the hydroxyl and alkynyl group of 2–propynol benzyl azides **1** with Zn(OTf)_2_ afforded complex **A**. Intermediate **A** was then intramolecularly attacked by the azides group to generate allenylaminodiazonium intermediate **B**. Subsequently, intermediate **B** undergoes intramolecular cyclization to generate the intermediate **C**. Finally, the nucleophilic attack on intermediate **C** by diphenylphosphine oxide **2a** followed by the loss of a proton produced the desired product **3**.

## 3. Materials and Methods

**General Remarks:** Column chromatography was carried out on silica gel. ^1^H–NMR spectra were recorded on 400 MHz in CDCl_3_ and ^13^C–NMR spectra were recorded on 100 MHz in CDCl_3_. Chemical shifts (ppm) were recorded with tetramethylsilane (TMS) as the internal reference standard. Multiplicities are given as: s (singlet), d (doublet), t (triplet), dd (doublet of doublets), q (quartet), or m (multiplet). High-resolution mass spectrometry (HRMS) was performed on a TOF/Q–TOF mass spectrometer. Melting points were determined on a microscopic apparatus and were uncorrected. Copies of the ^1^H–NMR and ^13^C–NMR spectra are provided in the Supplementary Materials. Commercially available reagents were used without further purification. All solvents were dried under standard method.

**General Procedure for the Construction of Phosphorylated Isoindoline Fused with Triazoles 3:** To a seal tube was added Zn(OTf)_2_ (0.04 mmol), ortho–propynol benzyl azides (**1**) (0.2 mmol), diphenylphosphine oxide **2a** (0.5 mmol), in CH_3_CN at 100°C under air atmosphere. After 1.0 h, as monitored by TLC, the reaction mixture was concentrated in vacuum and purified by column chromatography to generate **3**.

**((8H–[1,2,3]triazolo[5,1–*a*]isoindol–3–yl)diphenylmethyl)diphenylphosphine oxide (3a):** The title compound was prepared according to the general procedure and purified by column chromatography (silica gel, petroleum ether/ethyl acetate) to give a product **3a** (86%); Rf = 0.47 (petroleum ether/ethyl acetate 50:20); white solid; mp: 172–174 °C. ^1^H–NMR (400 MHz, CDCl_3_): δ ppm 5.30–5.32 (m, 3 H), 6.89–6.90 (m, 1 H), 7.15–7.28 (m, 11 H), 7.31–7.36 (m, 3 H), 7.42–7.44 (m, 4 H), 7.80–7.84 (m, 4 H). ^13^C-NMR (100 MHz, CDCl_3_): δ ppm 50.8, 59.5, 60.1, 123.2, 127.3, 127.4, 127.5, 128.0, 128.1, 128.2, 128.3, 130.6, 130.6, 131.0, 131.0, 132.6, 133.5, 134.3, 134.3, 139.7, 139.7, 140.5, 141.1, 143.1, 143.2. ^31^P NMR (162 MHz, CDCl_3_): δ ppm 40.31. HRMS (ESI, *m*/*z*): calcd for C_34_H_26_N_3_OP: M + H = 524.1886; found: 524.1883.

**((8H–[1,2,3]triazolo[5,1–*a*]isoindol–3-yl)di–p–tolylmethyl)diphenylphosphine oxide (3b):** The title compound was prepared according to the general procedure and purified by column chromatography (silica gel, petroleum ether/ethyl acetate) to give a product **3b** (91%); Rf = 0.47 (petroleum ether/ethyl acetate 50:20); yellow liquid. ^1^H–NMR (400 MHz, CDCl_3_): δ ppm 2.21 (s, 6 H), 5.19 (s, 2 H), 5.30 (d, *J* = 8.0 Hz, 1 H), 6.80 (t, *J* = 7.6 Hz, 1 H), 6.91–6.93 (m, 4 H), 7.06–7.09 (m, 1 H), 7.12–7.17 (m, 4 H), 7.19–7.26 (m, 7 H), 7.69–7.74 (m, 4 H). ^13^C–NMR (100 MHz, CDCl_3_): δ ppm 20.9, 50.7, 58.7, 59.3, 123.0, 123.3, 127.1, 127.2, 127.8, 128.0, 128.1, 128.8, 128.8, 130.3, 130.3, 130.8, 132.7, 133.6, 134.1, 134.2, 136.6, 136.7, 136.9, 137.0, 140.4, 141.3, 141.3, 143.0, 143.0. ^31^P NMR (162 MHz, CDCl_3_): δ ppm 40.13. HRMS (ESI, *m*/*z*): calcd for C_36_H_30_N_3_OP: M + H = 552.2199; found: 552.2197.

**((8H–[1,2,3]triazolo[5,1–*a*]isoindol–3–yl)bis(4–methoxyphenyl)methyl)diphenylphosphine oxide (3c):** The title compound was prepared according to the general procedure and purified by column chromatography (silica gel, petroleum ether/ethyl acetate) to give a product **3c** (81%); Rf = 0.47 (petroleum ether/ethyl acetate 50:20); white solid; mp: 133–135 °C. ^1^H–NMR (400 MHz, CDCl_3_): δ ppm 3.77 (s, 6 H), 5.31 (s, 2 H), 5.56 (d, *J* = 8.0 Hz, 1 H), 6.74 (d, *J* = 8.8 Hz, 4 H), 6.95 (t, *J* = 7.6 Hz, 1 H), 7.17–7.26 (m, 5 H), 7.30–7.37 (m, 7 H), 7.73–7.78 (m, 4 H). ^13^C-NMR (100 MHz, CDCl_3_): δ ppm 50.8, 55.2, 57.9, 58.5, 113.4, 123.1, 123.3, 127.2, 127.3, 127.9, 128.1, 128.4, 130.9, 131.0, 131.7, 131.7, 131.8, 131.8, 132.6, 133.6, 134.1, 134.2, 140.5, 141.4, 143.0, 143.0, 158.7. ^31^P NMR (162 MHz, CDCl_3_): δ ppm 39.72. HRMS (ESI, *m*/*z*): calcd for C_36_H_30_N_3_O_3_P: M + H = 584.2098; found: 584.2096.

**((8H–[1,2,3]triazolo[5,1–*a*]isoindol–3-yl)bis(4–fluorophenyl)methyl)diphenylphosphine oxide (3d):** The title compound was prepared according to the general procedure and purified by column chromatography (silica gel, petroleum ether/ethyl acetate) to give a product **3d** (73%); Rf = 0.47 (petroleum ether/ethyl acetate 50:20); yellow solid; mp: 105–107 °C. ^1^H–NMR (400 MHz, CDCl_3_): δ ppm 5.32 (s, 2 H), 5.45 (d, *J* = 7.6 Hz, 1 H), 6.89–6.99 (m, 5 H), 7.20–7.24 (m, 3 H), 7.26–7.28 (m, 2 H), 7.34–7.40 (m, 7 H), 7.79–7.84 (m, 4 H). ^13^C–NMR (100 MHz, CDCl_3_): δ ppm 50.9, 58.2, 58.8, 115.0, 115.2, 122.7, 123.4, 127.4, 127.5, 127.7, 128.3, 128.5, 131.3, 131.3, 131.9, 132.1, 132.1, 132.1, 132.2, 132.8, 134.1, 134.2, 135.3, 140.5, 140.7, 143.0, 143.0, 160.7, 163.2. ^31^P NMR (162 MHz, CDCl_3_): δ ppm 35.74. HRMS (ESI, *m*/*z*): calcd for C_34_H_24_F_2_N_3_OP: M + H = 560.1698; found: 560.1698.

**((8H–[1,2,3]triazolo[5,1–*a*]isoindol–3–yl)bis(4–chlorophenyl)methyl)diphenylphosphine oxide (3e):** The title compound was prepared according to the general procedure and purified by column chromatography (silica gel, petroleum ether/ethyl acetate) to give a product **3e** (74%); Rf = 0.47 (petroleum ether/ethyl acetate 50:20); yellow liquid. ^1^H–NMR (400 MHz, CDCl_3_): δ ppm 5.33 (s, 2 H), 5.45 (d, *J* = 8.0 Hz, 1 H), 6.99 (t, *J* = 8.0 Hz, 1 H), 7.18–7.22 (m, 4 H), 7.24–7.29 (m, 5 H), 7.32–7.40 (m, 7 H), 7.82–7.87 (m, 4 H). ^13^C-NMR (100 MHz, CDCl_3_): δ ppm 50.9, 58.6, 59.2, 122.8, 123.4, 127.5, 127.6, 128.4, 128.4, 128.6, 131.4, 131.4, 131.7, 131.8, 132.7, 133.7, 133.8, 134.2, 138.0, 138.0, 140.2, 140.6, 143.0, 143.1. ^31^P NMR (162 MHz, CDCl_3_): δ ppm 40.45. HRMS (ESI, *m*/*z*): calcd for C_34_H_24_Cl_2_N_3_OP: M + H = 592.1107; found: 592.1105.

**((8H–[1,2,3]triazolo[5,1–*a*]isoindol–3–yl)(4–fluorophenyl)(4 methoxyphenyl)methyl)diphenylphosphine oxide (3f):** The title compound was prepared according to the general procedure and purified by column chromatography (silica gel, petroleum ether/ethyl acetate) to give a product **3f** (78%); Rf = 0.47 (petroleum ether/ethyl acetate 50:20); red liquid. ^1^H–NMR (400 MHz, CDCl_3_): δ ppm 3.77 (s, 3 H), 5.31 (s, 2 H), 5.50 (d, *J* = 7.6 Hz, 1 H), 6.75 (d, *J* = 8.8 Hz, 2 H), 6.88–6.89 (m, 3 H), 7.19–7.27 (m, 4 H), 7.30 (s, 1 H), 7.31–7.41 (m, 7 H), 7.76–7.82 (m, 4 H). ^13^C–NMR (100 MHz, CDCl_3_): δ ppm 50.8, 55.2, 58.0, 58.6, 113.6, 114.8, 115.0, 123.0, 123.3, 127.3, 127.4, 127.8, 128.1, 128.4, 131.1, 131.5, 131.5, 131.6, 132.2, 132.2, 132.2, 132.3, 133.2, 133.2, 134.1, 134.2, 135.7, 140.5, 141.0, 143.0, 143.0, 158.8, 160.7, 163.2. ^31^P NMR (162 MHz, CDCl_3_): δ ppm 35.36. HRMS (ESI, *m*/*z*): calcd for C_35_H_27_FN_3_O_2_P: M + H = 572.1898; found: 572.1894.

**((8H–[1,2,3]triazolo[5,1–*a*]isoindol-3–yl)(phenyl)(p–tolyl)methyl)diphenylphosphine oxide (3g):** The title compound was prepared according to the general procedure and purified by column chromatography (silica gel, petroleum ether/ethyl acetate) to give a product **3g** (79%); Rf = 0.47 (petroleum ether/ethyl acetate 50:20); yellow liquid. ^1^H–NMR (400 MHz, CDCl_3_): δ ppm 2.30 (s, 3 H), 5.29–5.36 (m, 3 H), 6.89 (t, *J* = 8.0 Hz, 1 H), 7.01–7.03 (m, 2 H), 7.16–7.26 (m, 8 H), 7.28–7.36 (m, 5 H), 7.40–7.42 (m, 2 H), 7.78–7.83 (m, 4 H). ^13^C–NMR (100 MHz, CDCl_3_): δ ppm 20.9, 50.8, 59.1, 59.7, 123.1, 123.2, 127.2, 127.2, 127.3, 127.3, 127.9, 128.0, 128.1, 128.2, 128.9, 130.3, 130.4, 130.5, 130.5, 130.9, 130.9, 132.5, 132.6, 133.5, 133.6, 134.2, 134.3, 136.5, 137.1, 139.8, 140.5, 141.2, 143.0, 143.1. ^31^P NMR (162 MHz, CDCl_3_): δ ppm 40.26. HRMS (ESI, *m*/*z*): calcd for C_35_H_28_N_3_OP: M + H = 538.2043; found: 538.2041.

**((8H–[1,2,3]triazolo[5,1–*a*]isoindol–3–yl)(4–methoxyphenyl)(phenyl)methyl)diphenylphosphine oxide (3h):** The title compound was prepared according to the general procedure and purified by column chromatography (silica gel, petroleum ether/ethyl acetate) to give a product **3h** (89%); Rf = 0.47 (petroleum ether/ethyl acetate 50:20); yellow liquid. ^1^H–NMR (400 MHz, CDCl_3_): δ ppm 3.68 (s, 3 H), 5.22 (s, 2 H), 5.34 (d, *J* = 7.6 Hz, 1 H), 6.67 (d, *J* = 9.2 Hz, 1 H), 6.83 (t, *J* = 8.0 Hz, 1 H), 7.10–7.18 (m, 9 H), 7.24–7.28 (m, 4 H), 7.32 (d, *J* = 8.0 Hz, 2 H), 7.68–7.74 (m, 4 H). ^13^C–NMR (100 MHz, CDCl_3_): δ ppm 50.8, 55.2, 58.7, 59.3, 113.4, 123.1, 123.2, 127.2, 127.2, 127.3, 127.3, 127.9, 128.0, 128.1, 128.3, 130.5, 130.5, 130.9, 131.4, 131.5, 131.7, 131.7, 132.5, 132.6, 133.4, 133.5, 134.1, 134.2, 134.2, 134.3, 139.9, 140.0, 140.5, 141.2, 143.0, 143.1, 158.7, 158.8. ^31^P NMR (162 MHz, CDCl_3_): δ ppm 40.17. HRMS (ESI, *m*/*z*): calcd for C_35_H_28_N_3_O_2_P: M + H = 554.1992; found: 554.1988.

**((8H–[1,2,3]triazolo[5,1–a]isoindol–3–yl)(4–fluorophenyl)(phenyl)methyl)diphenylphosphine oxide (3i):** The title compound was prepared according to the general procedure and purified by column chromatography (silica gel, petroleum ether/ethyl acetate) to give a product **3i** (88%); Rf = 0.47 (petroleum ether/ethyl acetate 50:20); yellow liquid. ^1^H–NMR (400 MHz, CDCl_3_): δ ppm 5.23 (s, 2 H), 5.29 (d, *J* = 7.6 Hz, 1 H), 6.80–6.86 (m, 3 H), 7.12–7.18 (m, 8 H), 7.24–7.29 (m, 2 H), 7.30–7.35(m, 5 H), 7.70–7.77 (m, 4 H). ^13^C–NMR (100 MHz, CDCl_3_): δ ppm 50.9, 58.9, 59.5, 114.9, 115.1, 123.0, 123.3, 127.3, 127.4, 127.4, 127.5, 127.6, 127.9, 128.2, 128.4, 128.4, 128.7, 130.4, 130.4, 131.2, 132.2, 132.3, 132.3, 132.4, 132.4, 133.1, 133.2, 134.2, 134.3, 134.3, 135.4, 139.5, 139.7, 140.6, 140.9, 143.1, 143.2, 160.8, 163.3. ^31^P NMR (162 MHz, CDCl_3_): δ ppm 40.61. HRMS (ESI, *m*/*z*): calcd for C_34_H_25_FN_3_OP: M + H = 542.1792; found: 542.1791.

**((8H–[1,2,3]triazolo[5,1–*a*]isoindol–3–yl)(2–fluorophenyl)(phenyl)methyl)diphenylphosphine oxide (3j):** The title compound was prepared according to the general procedure and purified by column chromatography (silica gel, petroleum ether/ethyl acetate) to give a product **3j** (84%); Rf = 0.47 (petroleum ether/ethyl acetate 50:20); yellow liquid. ^1^H–NMR (400 MHz, CDCl_3_): δ ppm 5.20 (s, 2 H), 5.66 (d, *J* = 8.0 Hz, 1 H), 6.71–6.76 (m, 1 H), 6.87 (t, *J* = 8.0 Hz, 1 H), 7.09–7.19 (m, 8 H), 7.19–7.29 (m, 5 H), 7.45–7.53 (m, 6 H), 8.24 (t, *J* = 8.0 Hz, 1 H). ^13^C–NMR (100 MHz, CDCl_3_): δ ppm 50.6, 56.6, 57.2, 117.0, 117.2, 123.0, 123.1, 123.9, 123.9, 127.1, 127.3, 127.5, 127.6, 127.7, 127.7, 127.7, 128.2, 128.2, 129.3, 129.4, 129.7, 129.8, 131.0, 131.1, 131.1, 131.2, 131.2, 131.3, 131.6, 131.8, 132.1, 132.2, 132.6, 132.7, 133.4, 133.5, 133.7, 133.8, 137.7, 138.7, 140.4, 142.8, 160.2, 160.3, 162.7, 162.8. ^31^P–NMR (162 MHz, CDCl_3_): δ ppm 38.49. HRMS (ESI, *m*/*z*): calcd for C_34_H_25_FN_3_OP: M + H = 542.1792; found: 542.1791.

**(5–(8H–[1,2,3]triazolo[5,1–*a*]isoindol–3–yl)–10,11–dihydro–5H–dibenzo[a,d][7]annulen–5–yl)diphenylphosphine oxide (3k):** The title compound was prepared according to the general procedure and purified by column chromatography (silica gel, petroleum ether/ethyl acetate) to give a product **3k** (49%); Rf = 0.47 (petroleum ether/ethyl acetate 50:20); yellow solid; mp: 276–278 °C. ^1^H–NMR (400 MHz, CDCl_3_): δ ppm 2.98–3.04 (m, 2 H), 4.01–4.07 (m, 2 H), 5.19 (s, 2 H), 6.02 (d, *J* = 7.6 Hz, 1 H), 6.33–6.35 (m, 2 H), 6.41–6.44 (m, 2 H), 6.87–6.90 (m, 3 H), 7.03–7.07 (m, 1 H), 7.08–7.10 (m, 2 H), 7.17–7.23 (m, 6 H), 7.25–7.29 (m, 1 H), 8.10–8.14 (m, 4 H). ^13^C–NMR (100 MHz, CDCl_3_): δ ppm 37.1, 51.1, 121.8, 123.3, 124.9, 127.1, 127.2, 127.3, 127.7, 127.8, 128.5, 130.5, 130.9, 131.9, 132.8, 134.2, 134.4, 134.5, 136.2, 140.3, 145.2, 146.5. ^31^P–NMR (162 MHz, CDCl_3_): δ ppm 41.14. HRMS (ESI, *m*/*z*): calcd for C_36_H_28_N_3_OP: M + H = 550.2043; found: 550.2041.

**((6–methyl–8H–[1,2,3]triazolo[5,1–*a*]isoindol–3–yl)diphenylmethyl)diphenylphosphine oxide (3l):** The title compound was prepared according to the general procedure and purified by column chromatography (silica gel, petroleum ether/ethyl acetate) to give a product **3l** (78%); Rf = 0.47 (petroleum ether/ethyl acetate 50:20); yellow solid; mp: 252–254 °C. ^1^H–NMR (400 MHz, CDCl_3_): δ ppm 2.19 (s, 3 H), 5.08 (d, *J* = 8.0 Hz, 1 H), 5.17 (s, 2 H), 6.61 (d, *J* = 8.0 Hz, 1 H), 7.08–7.18 (m, 11 H), 7.23–7.27 (m, 2 H), 7.33–7.35 (m, 4 H), 7.73–7.78 (m, 4 H). ^13^C–NMR (100 MHz, CDCl_3_): δ ppm 21.4, 50.7, 59.4, 60.0, 122.7, 123.8, 125.3, 127.2, 127.3, 127.4, 128.1, 129.0, 130.5, 130.5, 130.9, 130.9, 132.5, 133.5, 134.2, 134.3, 138.3, 139.6, 139.6, 140.5, 140.8, 143.1, 143.2. ^31^P–NMR (162 MHz, CDCl_3_): δ ppm 40.64. HRMS (ESI, *m*/*z*): calcd for C_35_H_28_N_3_OP: M + H = 538.2043; found: 538.2041.

**((6–bromo–8H–[1,2,3]triazolo[5,1–*a*]isoindol–3–yl)diphenylmethyl)diphenylphosphine oxide (3m):** The title compound was prepared according to the general procedure and purified by column chromatography (silica gel, petroleum ether/ethyl acetate) to give a product **3q** (71%); Rf = 0.47 (petroleum ether/ethyl acetate 50:20); red solid; mp: 274–276 °C. ^1^H–NMR (400 MHz, CDCl_3_): δ ppm 5.13 (d, *J* = 8.4 Hz, 1 H), 5.22 (s, 2 H), 6.96 (dd, *J* = 1.6, 8.4 Hz, 1 H), 7.12–7.19 (m, 10 H), 7.25–7.28 (m, 2 H), 7.29–7.33 (m, 4 H), 7.42 (d, *J* = 0.8 Hz, 1 H), 7.64–7.69 (m, 4 H). ^13^C–NMR (100 MHz, CDCl_3_): δ ppm 50.4, 59.4, 60.0, 122.1, 124.5, 126.5, 127.0, 127.3, 127.4, 127.5, 128.3, 130.5, 130.6, 131.1, 131.1, 131.6, 132.2, 133.1, 134.0, 134.1, 139.5, 139.5, 141.3, 142.2, 142.4, 142.5. ^31^P–NMR (162 MHz, CDCl_3_): δ ppm 39.94. HRMS (ESI, *m*/*z*): calcd for C_34_H_25_BrN_3_OP: M + H = 602.0991; found: 602.0990.

**((8H–[1,2,3]triazolo[5,1–*a*]isoindol–3–yl)diphenylmethyl)bis(4–methoxyphenyl)phosphine oxide (3p):** The title compound was prepared according to the general procedure and purified by column chromatography (silica gel, petroleum ether/ethyl acetate) to give a product **3p** (82%); Rf = 0.47 (petroleum ether/ethyl acetate 50:20); yellow solid; mp: 118–120 °C. ^1^H–NMR (400 MHz, CDCl_3_): δ ppm 3.74 (s, 6 H), 5.30–5.34 (m, 3 H), 6.73–6.79 (m, 4 H), 6.88 (t, *J* = 8.0 Hz, 1 H), 7.14–7.25 (m, 7 H), 7.34 (d, *J* = 7.6 Hz, 1 H), 7.41–7.43 (m, 4 H), 7.66–7.71 (m, 4 H). ^13^C–NMR (100 MHz, CDCl_3_): δ ppm 50.7, 55.0, 59.3, 60.0, 112.7, 112.8, 123.1, 123.2, 123.8, 124.8, 127.3, 127.9, 128.0, 128.1, 128.2, 130.5, 130.6, 135.9, 136.0, 139.9, 139.9, 140.4, 141.3, 143.1, 143.1. ^31^P–NMR (162 MHz, CDCl_3_): δ ppm 40.00. HRMS (ESI, *m*/*z*): calcd for C_36_H_30_N_3_O_3_P: M+H = 584.2098; found: 584.2096.

**((8H–[1,2,3]triazolo[5,1–*a*]isoindol–3–yl)diphenylmethyl)di–m–tolylphosphine oxide (3q):** The title compound was prepared according to the general procedure and purified by column chromatography (silica gel, petroleum ether/ethyl acetate) to give a product **3q** (77%); Rf = 0.47 (petroleum ether/ethyl acetate 50:20); yellow liquid. ^1^H–NMR (400 MHz, CDCl_3_): δ ppm 2.13 (s, 6 H), 5.22 (s, 2 H), 5.36 (d, *J* = 8.0 Hz, 1 H), 6.82 (t, J = 7.6 Hz, 1 H), 7.01–7.10 (m, 5 H), 7.13–7.17 (m, 6 H), 7.26–7.28 (m, 1 H), 7.32 – 7.43 (m, 8 H). ^13^C–NMR (100 MHz, CDCl_3_): δ ppm 21.3, 50.8, 59.5, 60.1, 123.1, 123.4, 127.1, 127.2, 127.4, 127.9, 128.1, 128.2, 128.3, 130.9, 130.9, 131.2, 131.2, 131.9, 131.9, 132.1, 133.0, 134.5, 134.6, 136.9, 137.0, 139.8, 140.5, 141.0, 143.2, 143.2. ^31^P–NMR (162 MHz, CDCl_3_): δ ppm 39.96. HRMS (ESI, *m*/*z*): calcd for C_36_H_30_N_3_OP: M + H = 552.2199; found: 552.2197.

**((8H–[1,2,3]triazolo[5,1–*a*]isoindol–3–yl)(4–methoxyphenyl)methyl)diphenylphosphine oxide (3s):** The title compound was prepared according to the general procedure and purified by column chromatography (silica gel, petroleum ether/ethyl acetate) to give a product **3s** (65%); Rf = 0.47 (petroleum ether/ethyl acetate 50:20); yellow liquid. ^1^H–NMR (400 MHz, CDCl_3_): δ ppm 3.62 (s, 3 H), 5.10 (s, 2 H), 5.45 (d, *J* = 12.0 Hz, 1 H), 6.62 (d, *J* = 8.8 Hz, 2 H), 7.22–7.35 (m, 10 H), 7.42–7.46(m, 1 H), 7.59–7.64 (m, 2 H), 7.72–7.77 (m, 2 H), 8.61 (d, *J* = 8.0 Hz, 1 H). ^13^C–NMR (100 MHz, CDCl_3_): δ ppm 45.7, 46.3, 50.8, 55.2, 113.9, 123.4, 124.3, 126.9, 126.9, 128.2, 128.2, 128.3, 128.5, 129.1, 130.8, 130.9, 131.1, 131.2, 131.3, 131.4, 131.5, 131.6, 131.6, 132.0, 132.1, 133.0, 135.0, 135.1, 140.5, 140.9, 140.9, 158.7. ^31^P–NMR (162 MHz, CDCl_3_): δ ppm 30.54. HRMS (ESI, *m*/*z*): calcd for C_29_H_24_N_3_O_2_P: M + H = 478.1679; found: 478.1673.

**((8H–[1,2,3]triazolo[5,1–*a*]isoindol–3–yl)(3,4–dimethoxyphenyl)methyl)diphenylphosphine oxide (3t):** The title compound was prepared according to the general procedure and purified by column chromatography (silica gel, petroleum ether/ethyl acetate) to give a product **3t** (57%); Rf = 0.47 (petroleum ether/ethyl acetate 50:20); yellow solid; mp: 225–227 °C. ^1^H–NMR (400 MHz, CDCl_3_): δ ppm 3.52 (s, 3 H), 3.69 (s, 3 H), 5.12 (s, 2 H), 5.45 (d, *J* = 11.6 Hz, 1 H), 6.58 (d, *J* = 8.4 Hz, 1 H), 6.87–6.89 (m, 2 H), 7.24–7.38 (m, 8 H), 7.45 (t, *J* = 7.2 Hz, 1 H), 7.60–7.64 (m, 2 H), 7.75–7.80 (m, 2 H), 8.72 (d, *J* = 8.0 Hz, 1 H). ^13^C–NMR (100 MHz, CDCl_3_): δ ppm 46.0, 46.7, 50.8, 55.6, 55.7, 110.8, 112.8, 112.8, 122.0, 122.0, 123.4, 124.3, 127.1, 127.2, 128.1, 128.3, 128.3, 128.4, 128.4, 128.9, 130.9, 131.1, 131.2, 131.3, 131.4, 131.5, 131.6, 131.6, 131.6, 131.9, 132.0, 133.0, 134.8, 134.8, 140.5, 140.8, 140.9, 148.0, 148.5. ^31^P–NMR (162 MHz, CDCl_3_): δ ppm 30.51. HRMS (ESI, *m*/*z*): calcd for C_30_H_26_N_3_O_3_P: M + H = 508.1785; found: 508.1783.

## 4. Conclusions

We have successfully described a general and novel Zn(OTf)_2_-catalyzed cascade cyclization of ortho–propynol benzyl azides and diphenylphosphine oxides. In this transformation, a series of alkynols substrates with various functional groups could be tolerated to form the corresponding phosphorylated isoindolines fused with triazoles in moderate to excellent yields. This reaction likely proceeds via the formation of allenylaminodiazonium intermediate followed by cyclization and nuclophilic attack of diphenylphosphine oxide. Moreover, this reaction can be performed in gram-scale with good yield, which could lead to potential application in organic synthesis. Further studies into the use of this novel strategy for the construction of other functionalized heterocyclics fused with triazoles are ongoing in our group.

## Figures and Tables

**Table 1:**
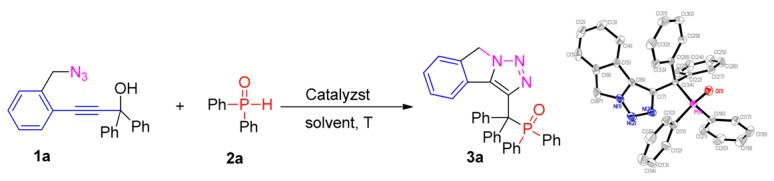
Optimization of the reaction for the synthesis of **3a** *^a^*.

Entry	Catalyst (x mol%)	Solvent	*T* [°C]	Yield [%]
1	Sc(OTf)_3_ (30)	DCE	100	42
2	Zn(OTf)_2_ (30)	DCE	100	53
3	Cu(OTf)_2_ (30)	DCE	100	trace
4	Cu(OAc)_2_ (30)	DCE	100	trace
5	CuCl_2_ (30)	DCE	100	<5
6	AgOTf (30)	DCE	100	trace
7	Zn(OTf)_2_ (30)	MeNO_2_	100	61
8	Zn(OTf)_2_ (30)	CH_3_CN	100	81
9	Zn(OTf)_2_ (30)	1,4-dioxane	100	trace
10	Zn(OTf)_2_ (30)	DCM	40	32
11	Zn(OTf)_2_ (30)	CH_3_CN	80	75
12	Zn(OTf)_2_ (30)	CH_3_CN	110	80
13	Zn(OTf)_2_ (20)	CH_3_CN	100	82
14	Zn(OTf)_2_ (10)	CH_3_CN	100	67
15 *^b^*	Zn(OTf)_2_ (20)	CH_3_CN	100	71
16 *^c^*	Zn(OTf)_2_ (20)	CH_3_CN	100	86

*^a^* Unless otherwise noted, all reactions were performed with 0.2 mmol of **1a** with diphenylphosphine oxide **2a** (2.0 equiv) in solvent (2.0 mL) for 1.0 h. *^b^* 1.5 equiv. of **2a**. *^c^* 2.5 equiv. of **2a**.

## Supplementary Materials

The following are available online at https://www.mdpi.com/1420-3049/24/19/3526/s1.
