# Supplementary material for: An Efficient Approach to Phosphorylated Isoindoline Fused with Triazoles via Zn-Catalyzed Cascade Cyclization of 2–Propynol Benzyl Azides and Diarylphosphine Oxides
Source: Molecules. 2019 Sep 29;24(19):3526. doi: 10.3390/molecules24193526 (PMC6803861; doi:10.3390/molecules24193526)
Supplement: Supplementary file 1 [file molecules-24-03526-s001.pdf]

An Efficient Approach to Phosphorylated Isoindoline Fused with Triazoles *via* Zn-Catalyzed Cascade Cyclization of 2-Propynol Benzyl Azides and Diarylphosphine Oxides

Tao Yang,[1] Xianrong Song,*[1] Ruchun Yang,[1] Haixin Ding,[1] Jiang Bai[1] and Qiang Xiao*[1]

[1] *Institute of Organic Chemistry, Jiangxi Science & Technology Normal University,*

Key Laboratory of Organic Chemistry, Jiangxi Province, Nanchang 330013, China:

E-mail: songxr2015@163.com, xiaoqiang@tsinghua.org.cn .

Table of Contents

**1**	**Crystal preparation and X-ray diffraction analysis of compound 3a**	**S3**
**2**	**^{1}H NMR and ^{13}C NMR spectra for compounds 3a-3t**	**S4-S54**

Crystal preparation and X-ray diffraction analysis of compound 3a

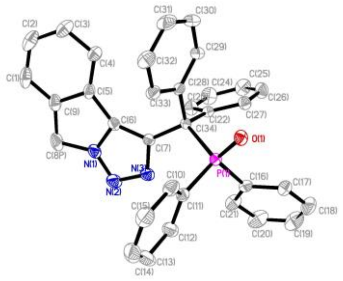

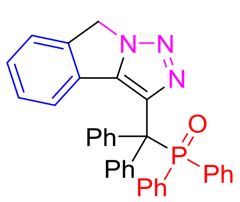

Datablock: 1

Bond precision: C-C = 0.0047 A Wavelength=0.71073

Cell: a=10.3355(13) b=15.828(2) c=19.435(2)
alpha=90.738(2) beta=101.662(2) gamma=107.843(2)
Temperature: 296 K

	Calculated	Reported
Volume	2954.4(6)	2954.3(6)
Space group	P -1	P -1
Hall group	-P 1	-P 1
Moiety formula	2(C34 H26 N3 O P), C4 H8 O2	?
Sum formula	C72 H60 N6 O4 P2	C72 H60 N6 O4 P2
Mr	1135.20	1135.20
Dx,g cm-3	1.276	1.276
Z	2	2
Mu (mm-1)	0.131	0.131
F000	1192.0	1192.0
F000'	1192.85	
h,k,lmax	12,19,23	12,19,23
Nref	11618	11539
Tmin,Tmax	0.961,0.965	
Tmin'	0.961	

Correction method= Not given

Data completeness= 0.993 Theta(max)= 25.999

R(reflections)= 0.0563(7635) wR2(reflections)= 0.1784(11539)

S = 1.007 Npar= 757

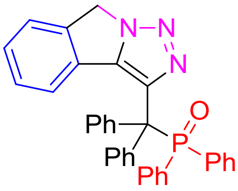
N
N
N
O
Ph
Ph
P
Ph
Ph

3a

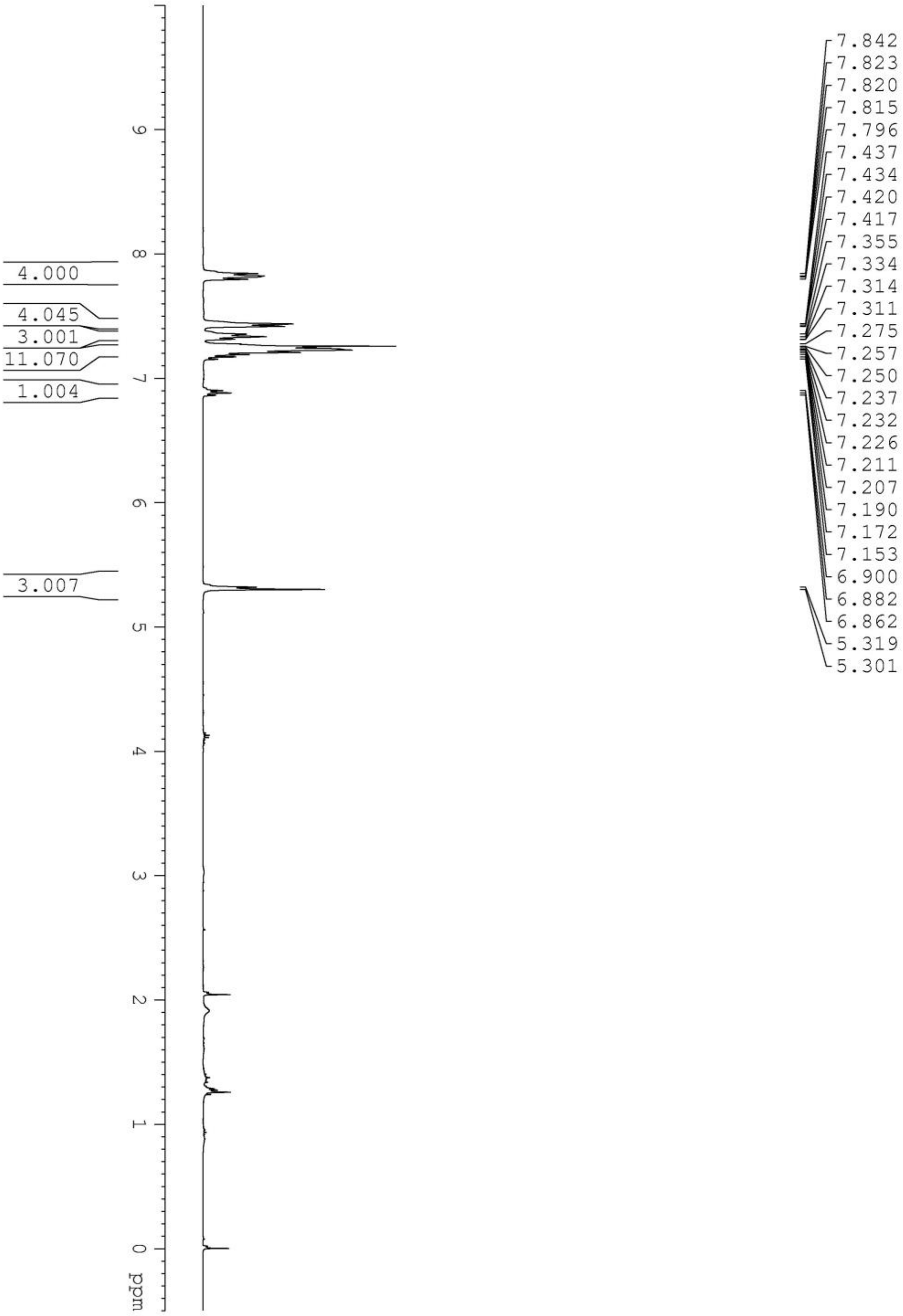
4.000
4.045
3.001
11.070
1.004
3.007
9
8
7
6
5
4
3
2
1
0
ppm
7.842
7.823
7.820
7.815
7.796
7.437
7.434
7.420
7.417
7.355
7.334
7.314
7.311
7.275
7.257
7.250
7.237
7.232
7.226
7.211
7.207
7.190
7.172
7.153
6.900
6.882
6.862
5.319
5.301

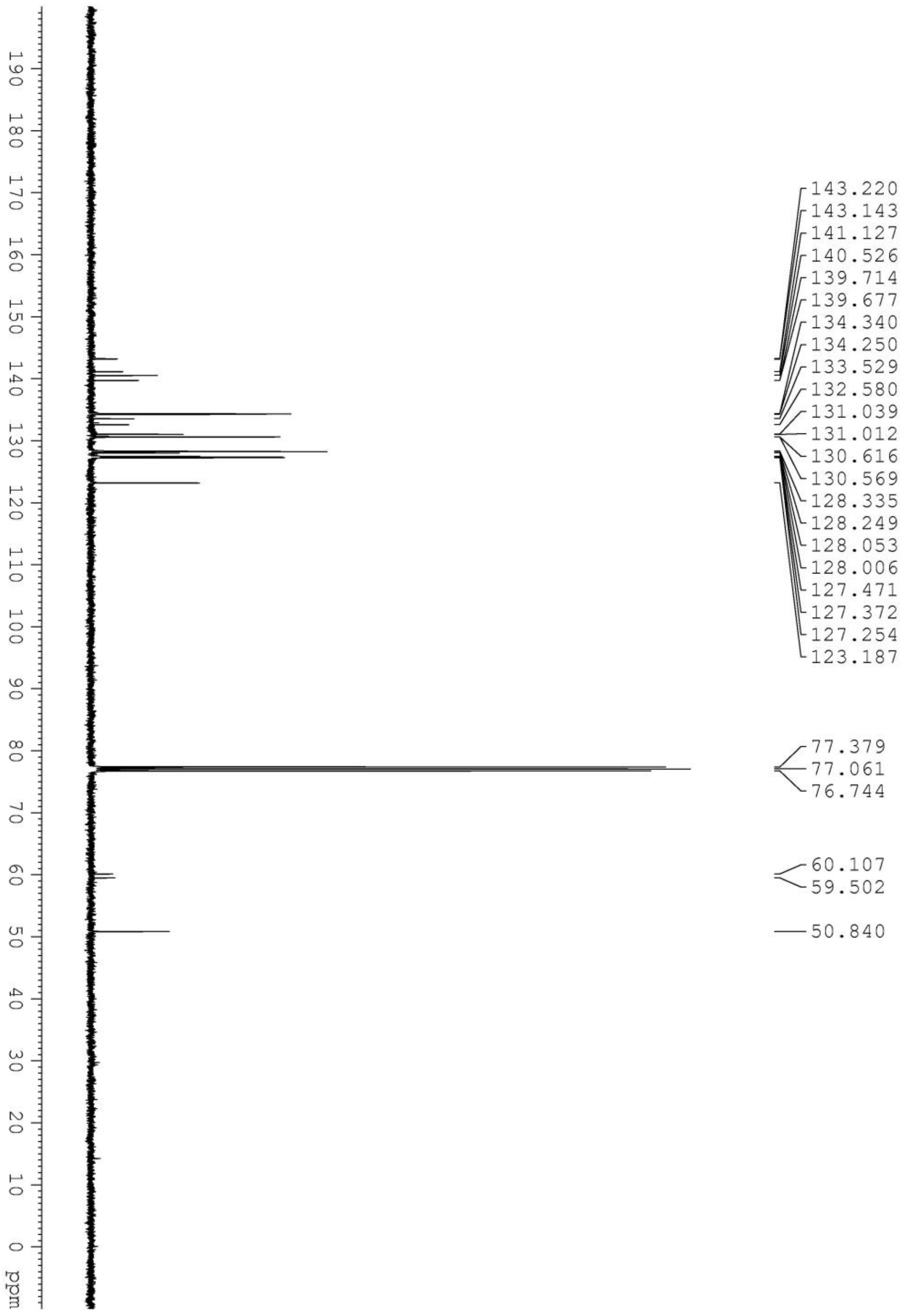
143.220
143.143
141.127
140.526
139.714
139.677
134.340
134.250
133.529
132.580
131.039
131.012
130.616
130.569
128.335
128.249
128.053
128.006
127.471
127.372
127.254
123.187
77.379
77.061
76.744
60.107
59.502
50.840
ppm

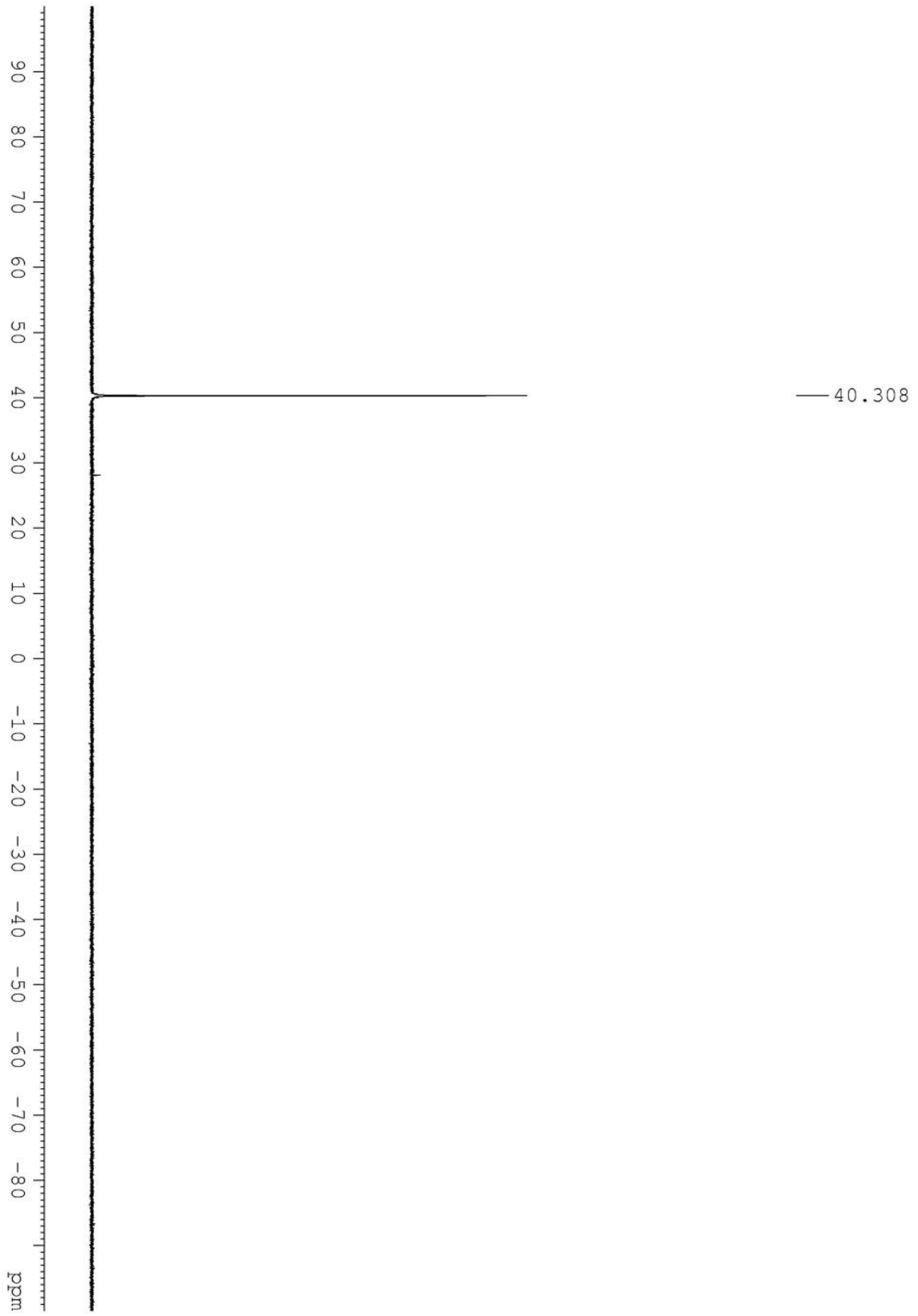
40.308
90
80
70
60
50
40
30
20
10
0
-10
-20
-30
-40
-50
-60
-70
-80
ppm

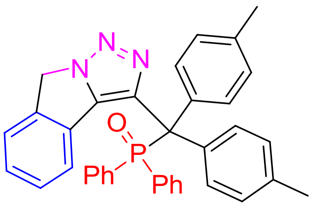
N
N
N
O
P
Ph
Ph

3b

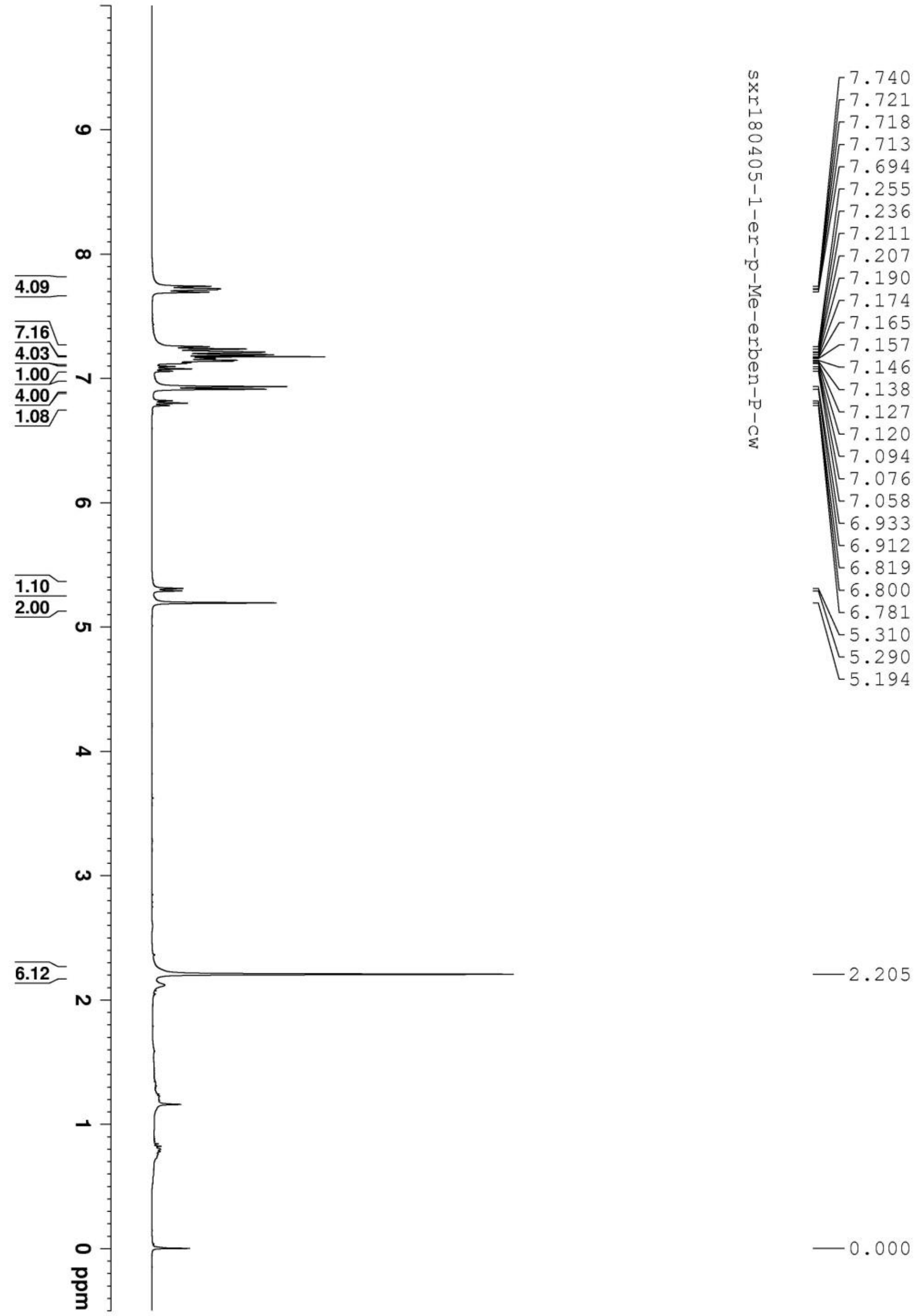
sxr180405-1-er-p-Me-erben-P-cw
7.740
7.721
7.718
7.713
7.694
7.255
7.236
7.211
7.207
7.190
7.174
7.165
7.157
7.146
7.138
7.127
7.120
7.094
7.076
7.058
6.933
6.912
6.819
6.800
6.781
5.310
5.290
5.194
2.205
0.000
4.09
7.16
4.03
1.00
4.00
1.08
1.10
2.00
6.12
ppm

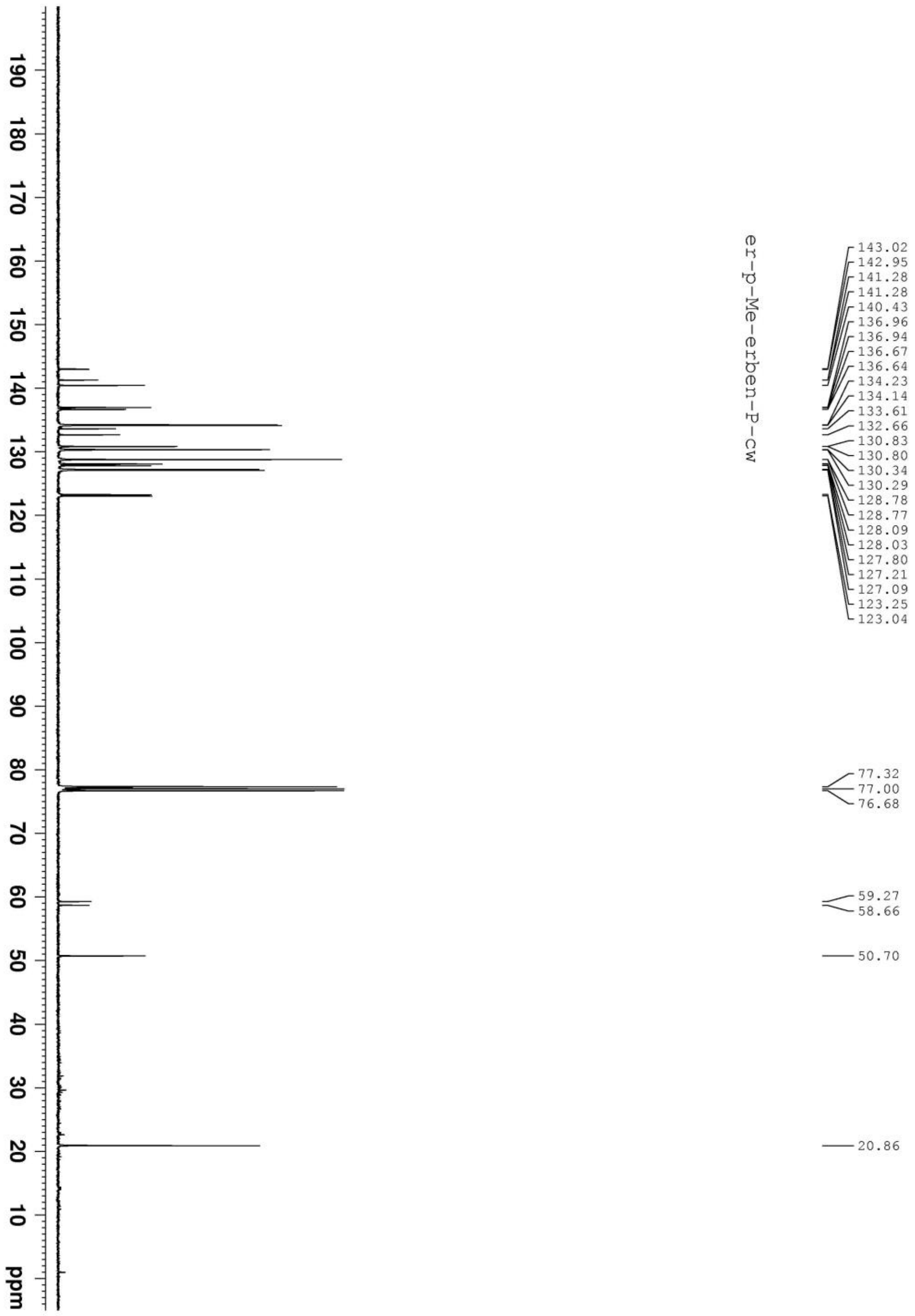

er-p-Me-erben-P-cw
143.02
142.95
141.28
141.28
140.43
136.96
136.94
136.67
136.64
134.23
134.14
133.61
132.66
130.83
130.80
130.34
130.29
128.78
128.77
128.09
128.03
127.80
127.21
127.09
123.25
123.04
77.32
77.00
76.68
59.27
58.66
50.70
20.86
190
180
170
160
150
140
130
120
110
100
90
80
70
60
50
40
30
20
10
ppm

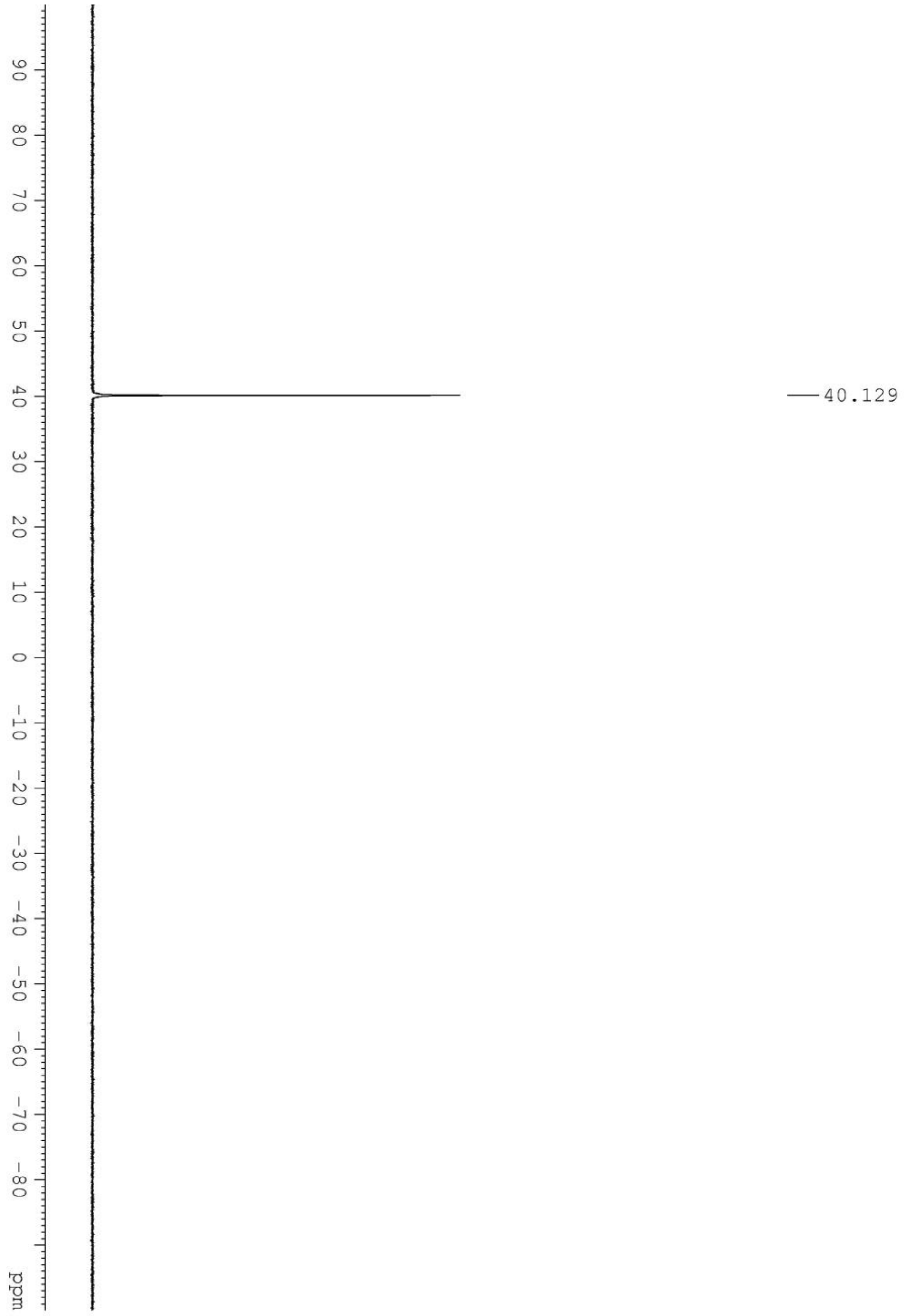
40.129
90
80
70
60
50
40
30
20
10
0
-10
-20
-30
-40
-50
-60
-70
-80
ppm

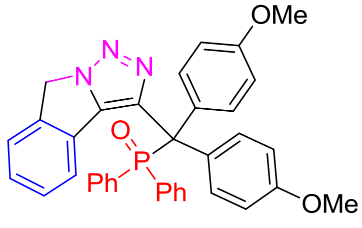
OMe
N
N
N
O
P
Ph
Ph
OMe

3c

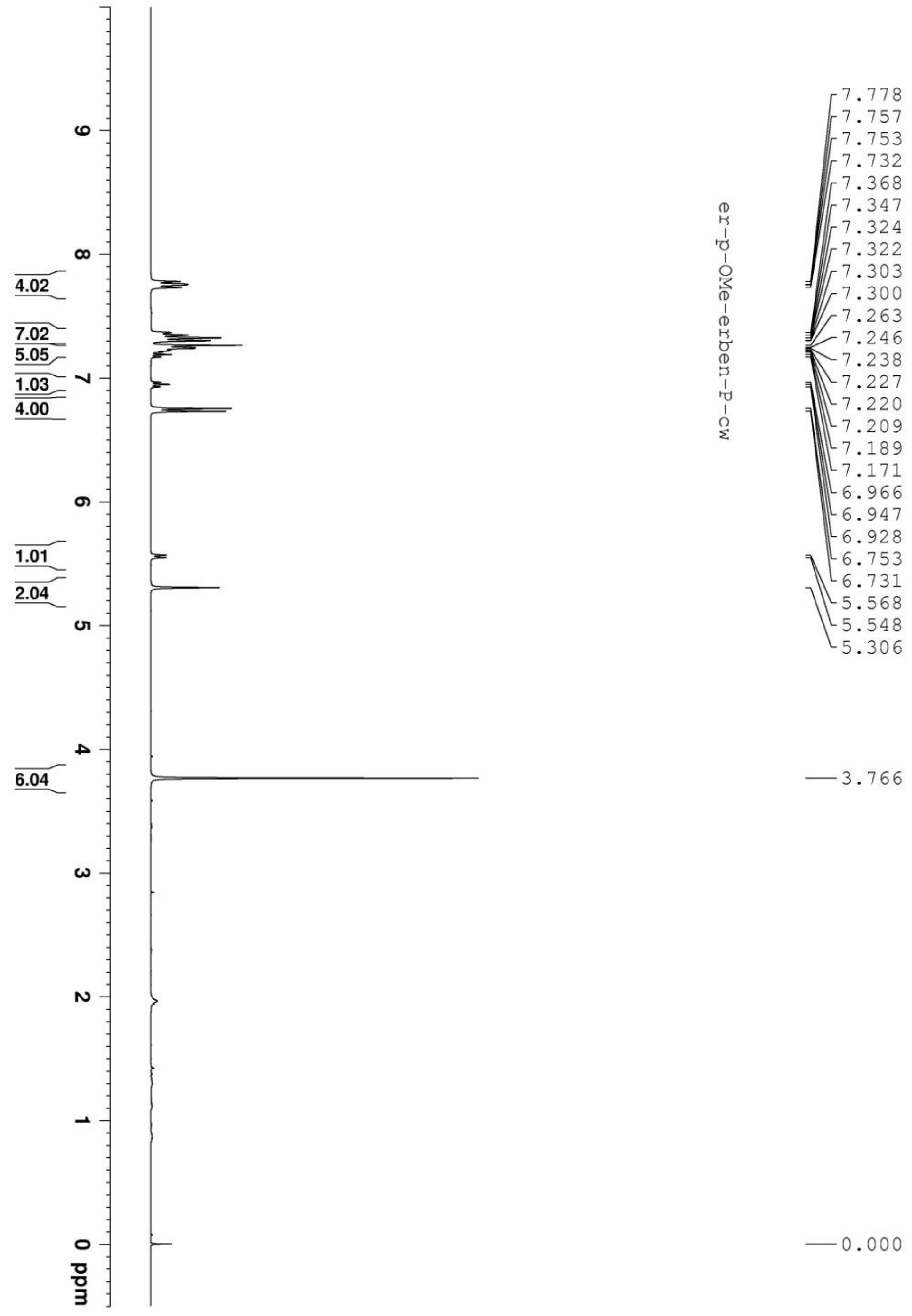
ppm
9
8
7
6
5
4
3
2
1
0
4.02
7.02
5.05
1.03
4.00
1.01
2.04
6.04
er-p-OMe-erben-P-cw
7.778
7.757
7.753
7.732
7.368
7.347
7.324
7.322
7.303
7.300
7.263
7.246
7.238
7.227
7.220
7.209
7.189
7.171
6.966
6.947
6.928
6.753
6.731
5.568
5.548
5.306
3.766
0.000

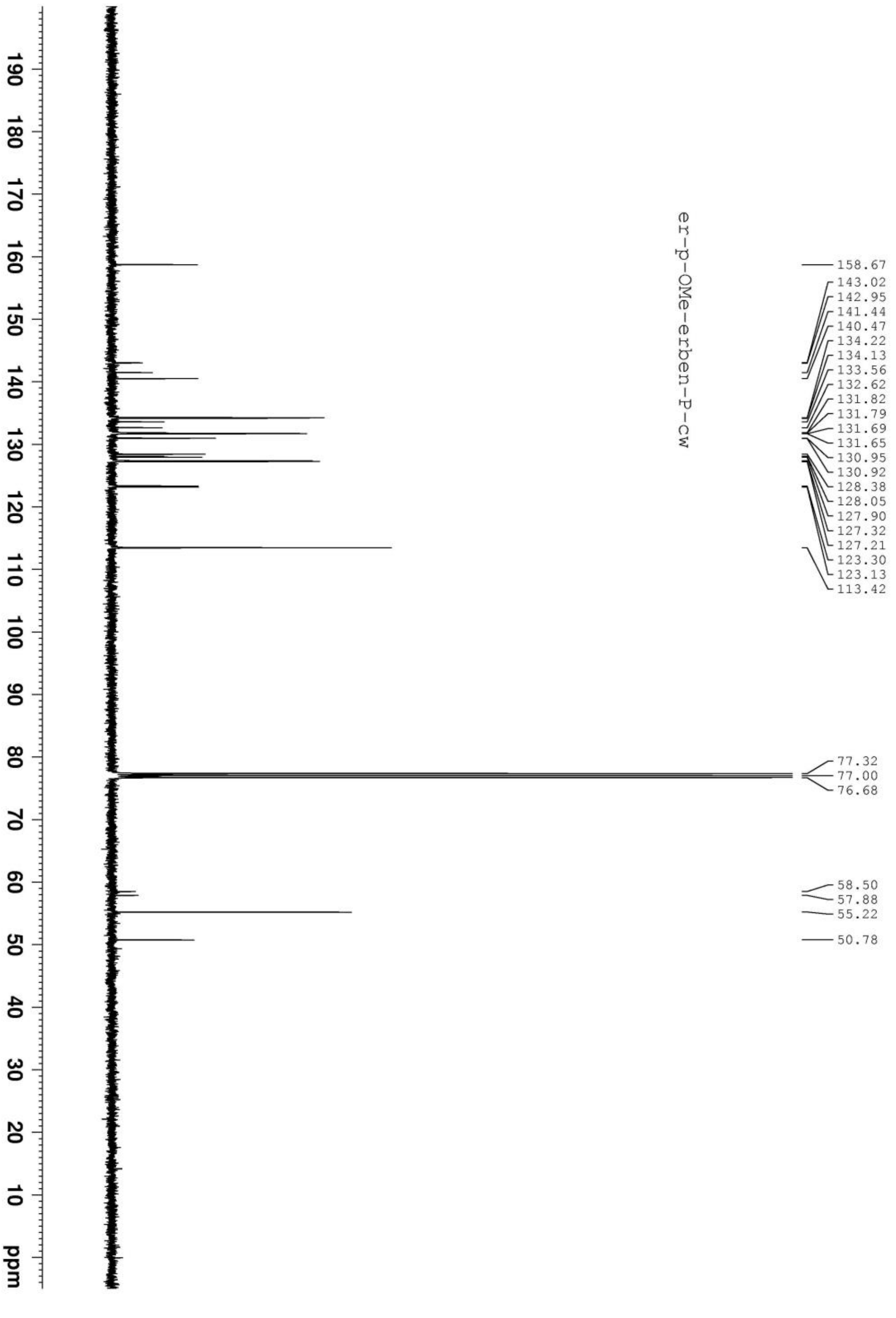
er-p-OMe-erben-P-cw
158.67
143.02
142.95
141.44
140.47
134.22
134.13
133.56
132.62
131.82
131.79
131.69
131.65
130.95
130.92
128.38
128.05
127.90
127.32
127.21
123.30
123.13
113.42
77.32
77.00
76.68
58.50
57.88
55.22
50.78
190
180
170
160
150
140
130
120
110
100
90
80
70
60
50
40
30
20
10
ppm

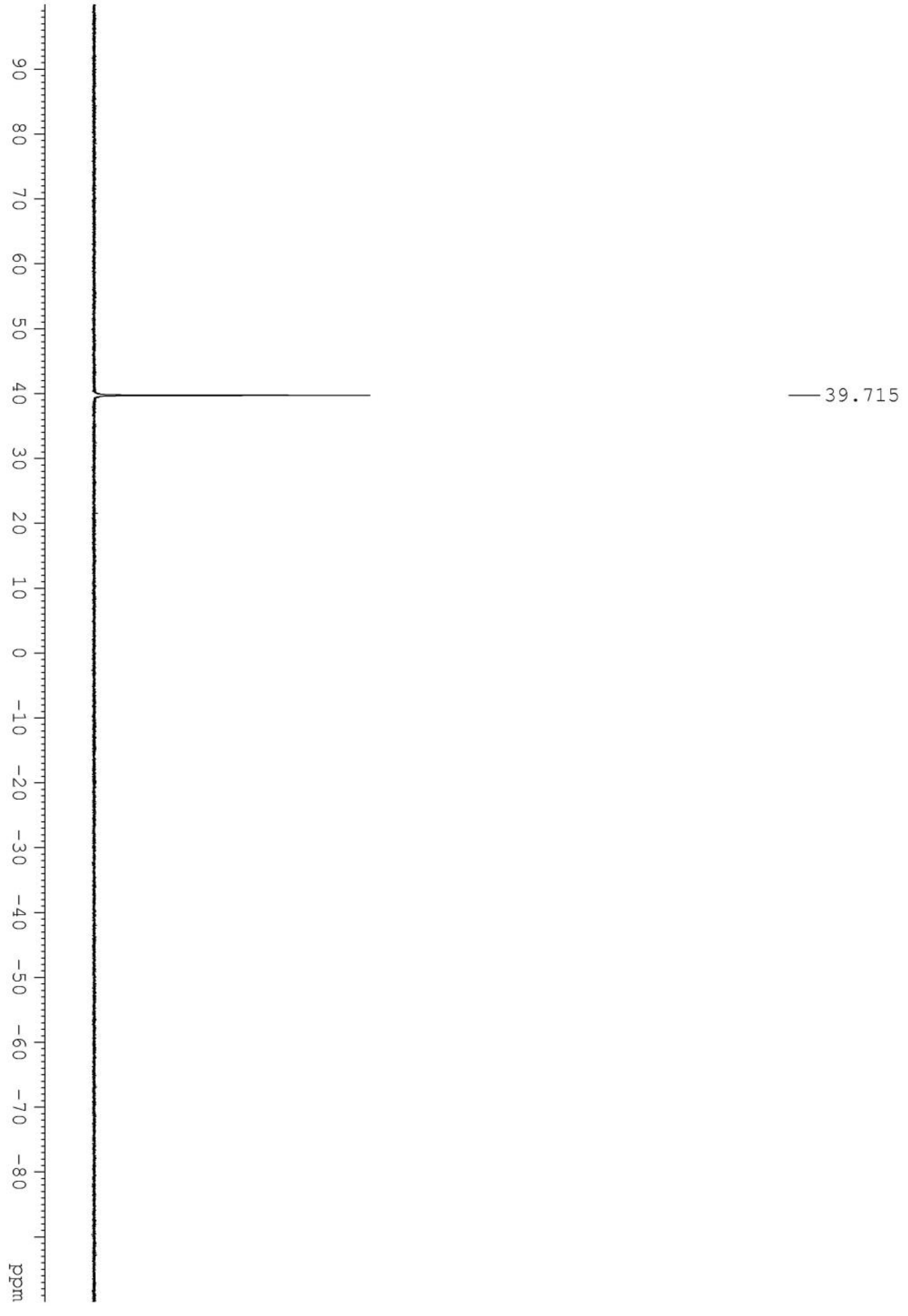

39.715
90
80
70
60
50
40
30
20
10
0
-10
-20
-30
-40
-50
-60
-70
-80
ppm

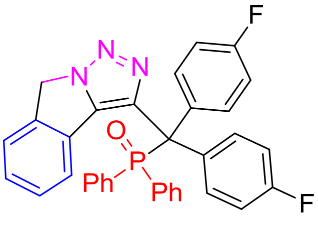
F
N
N
N
O
P
Ph
Ph
F

3d

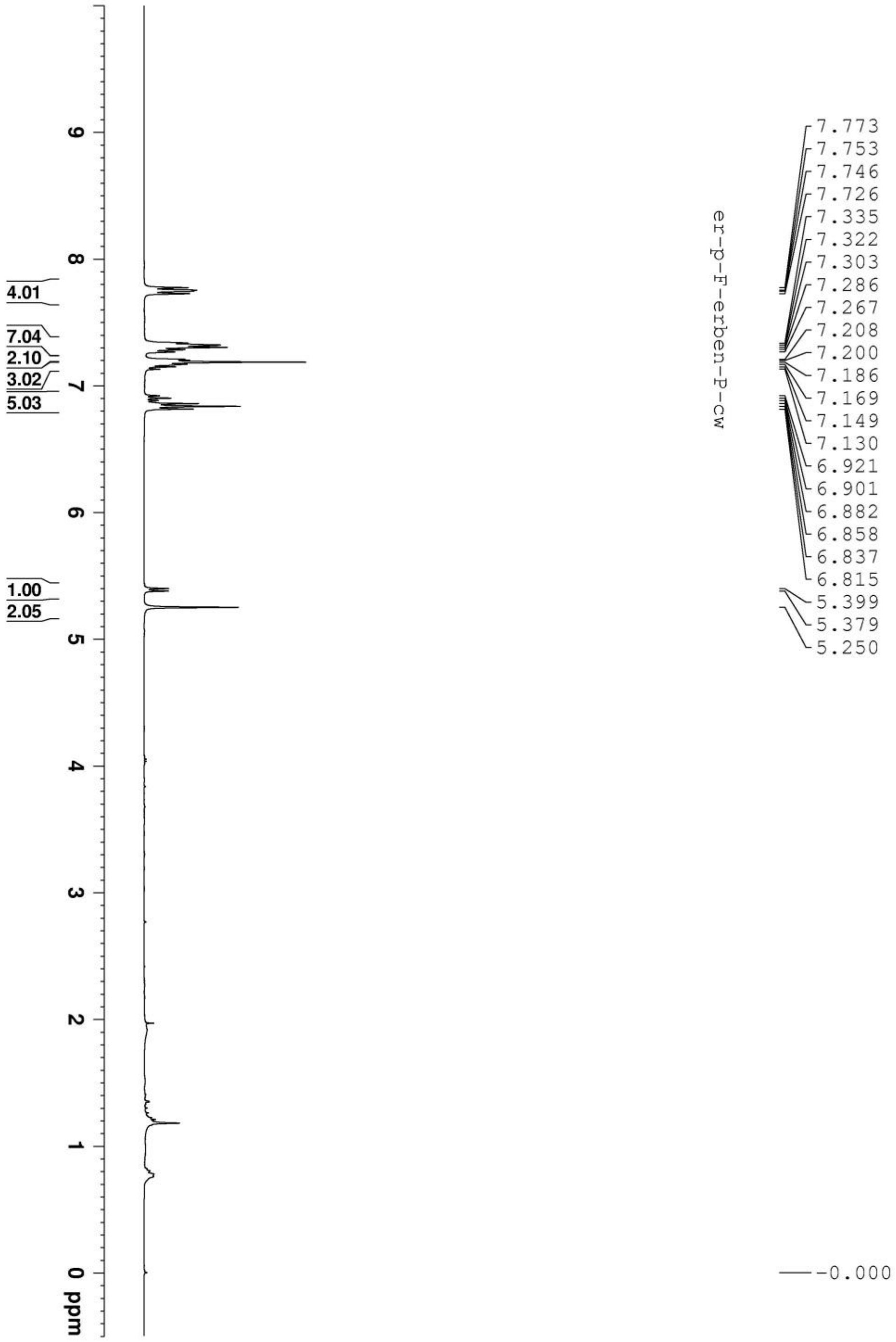
er-p-F-erben-P-cw
7.773
7.753
7.746
7.726
7.335
7.322
7.303
7.286
7.267
7.208
7.200
7.186
7.169
7.149
7.130
6.921
6.901
6.882
6.858
6.837
6.815
5.399
5.379
5.250
-0.000
4.01
7.04
2.10
3.02
5.03
1.00
2.05
9
8
7
6
5
4
3
2
1
0 ppm

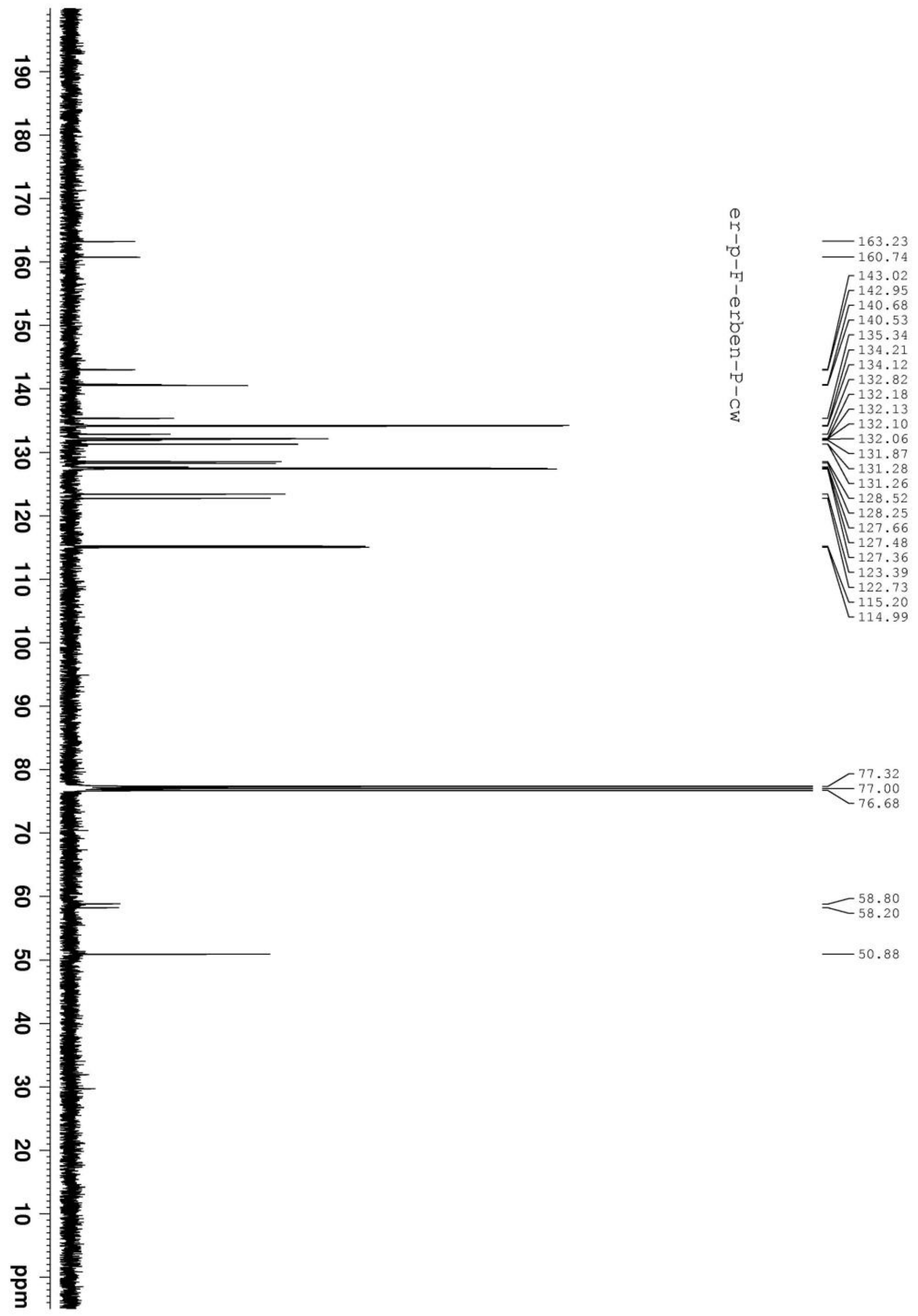
er-p-F-erben-P-cw
163.23
160.74
143.02
142.95
140.68
140.53
135.34
134.21
134.12
132.82
132.18
132.13
132.10
132.06
131.87
131.28
131.26
128.52
128.25
127.66
127.48
127.36
123.39
122.73
115.20
114.99
77.32
77.00
76.68
58.80
58.20
50.88
ppm

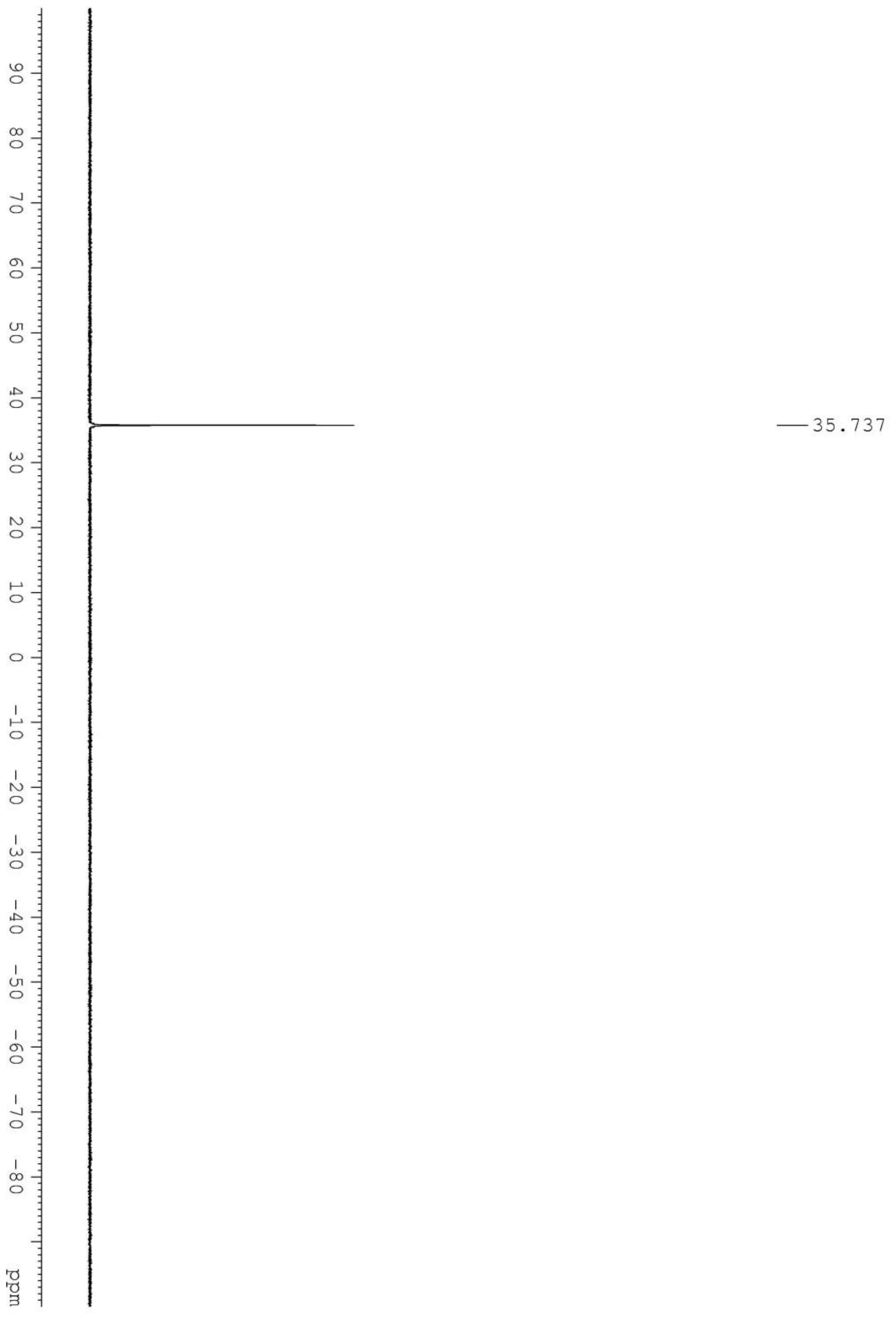
35.737
90
80
70
60
50
40
30
20
10
0
-10
-20
-30
-40
-50
-60
-70
-80
ppm

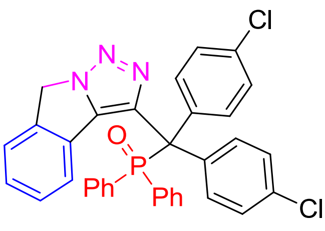
N
N
N
O
P
Ph
Ph
Cl
Cl

3e

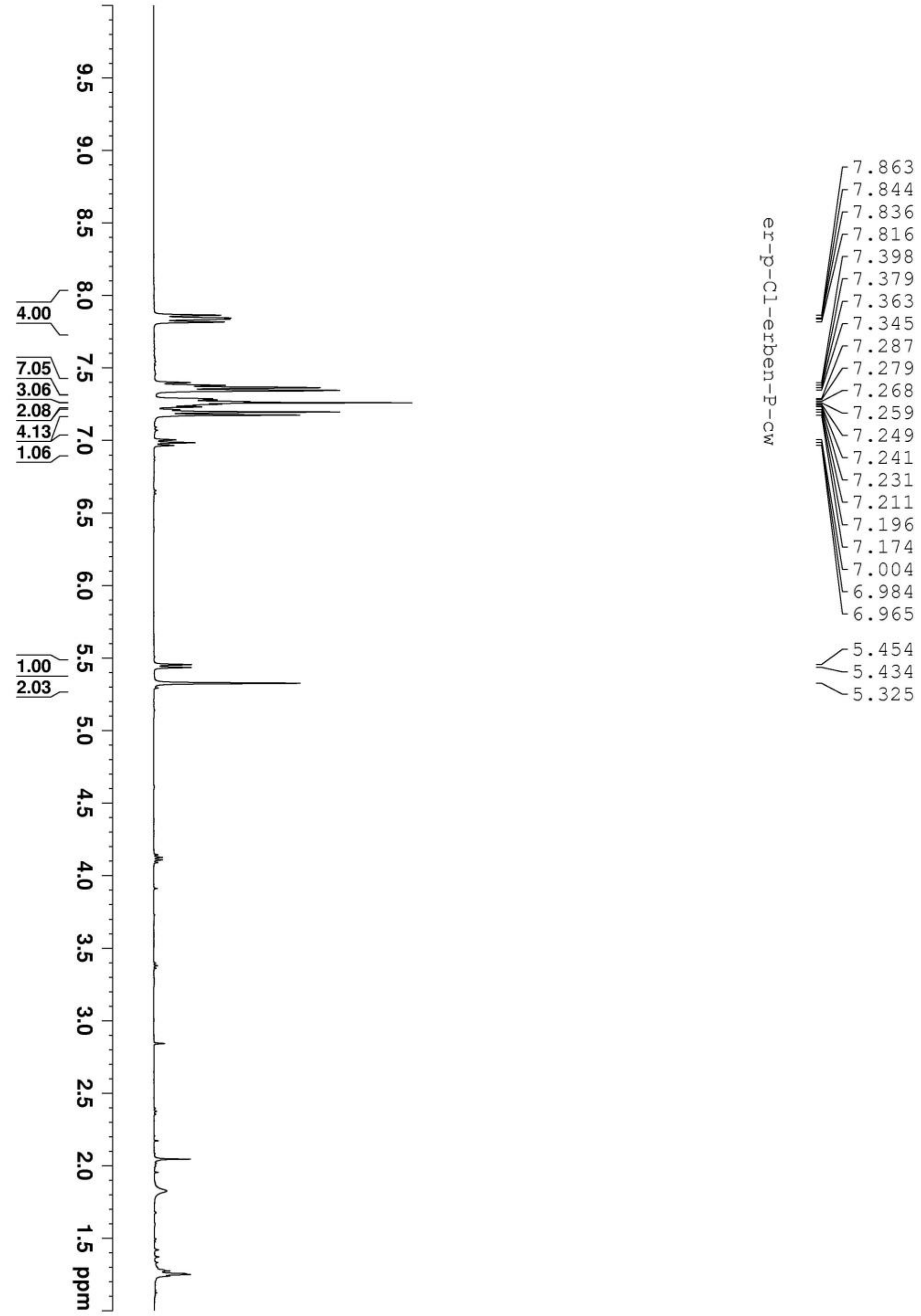
er-p-Cl-erben-P-cw
7.863
7.844
7.836
7.816
7.398
7.379
7.363
7.345
7.287
7.279
7.268
7.259
7.249
7.241
7.231
7.211
7.196
7.174
7.004
6.984
6.965
5.454
5.434
5.325
4.00
7.05
3.06
2.08
4.13
1.06
1.00
2.03
9.5
9.0
8.5
8.0
7.5
7.0
6.5
6.0
5.5
5.0
4.5
4.0
3.5
3.0
2.5
2.0
1.5 ppm

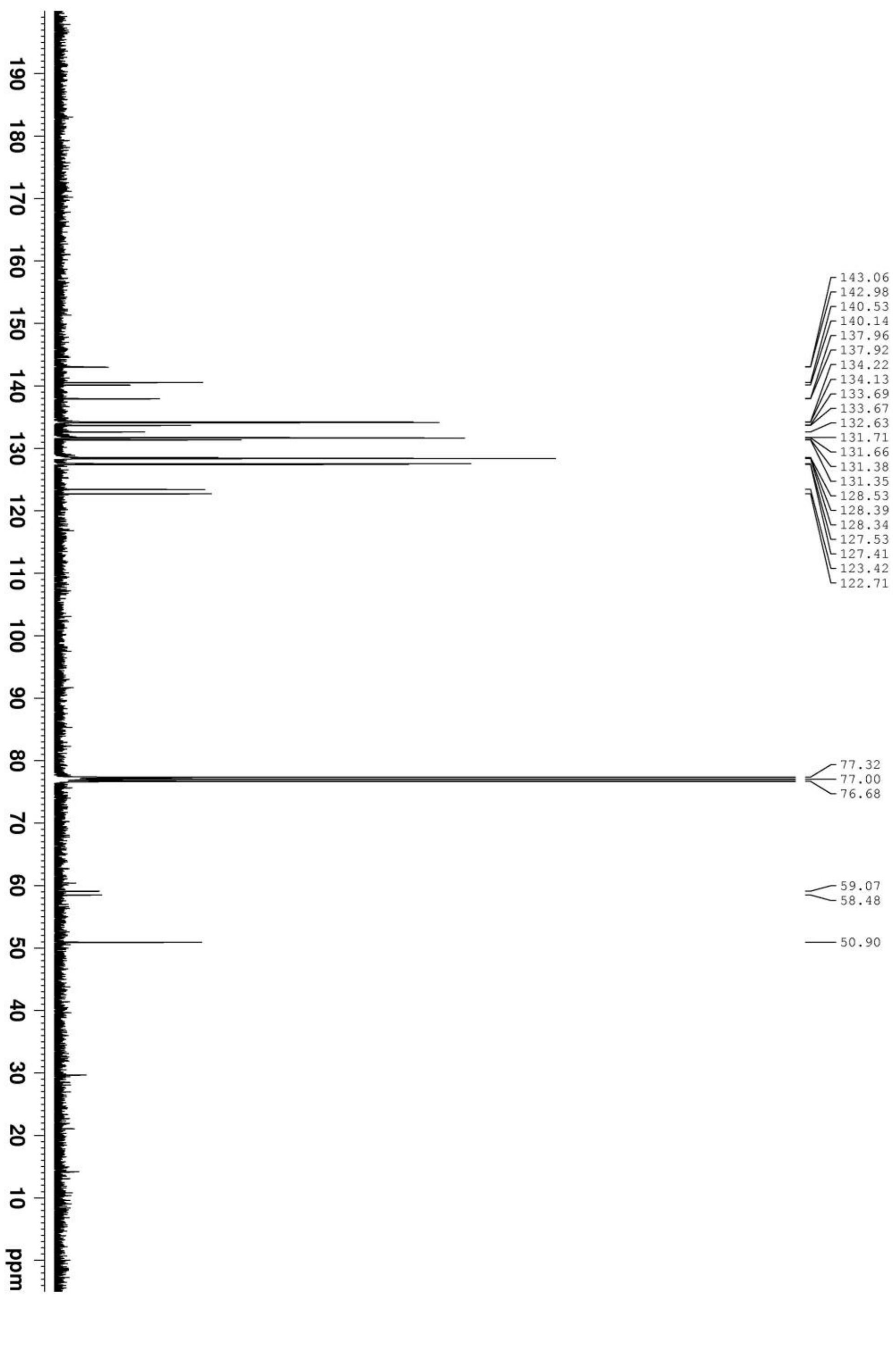
143.06
142.98
140.53
140.14
137.96
137.92
134.22
134.13
133.69
133.67
132.63
131.71
131.66
131.38
131.35
128.53
128.39
128.34
127.53
127.41
123.42
122.71
77.32
77.00
76.68
59.07
58.48
50.90
ppm

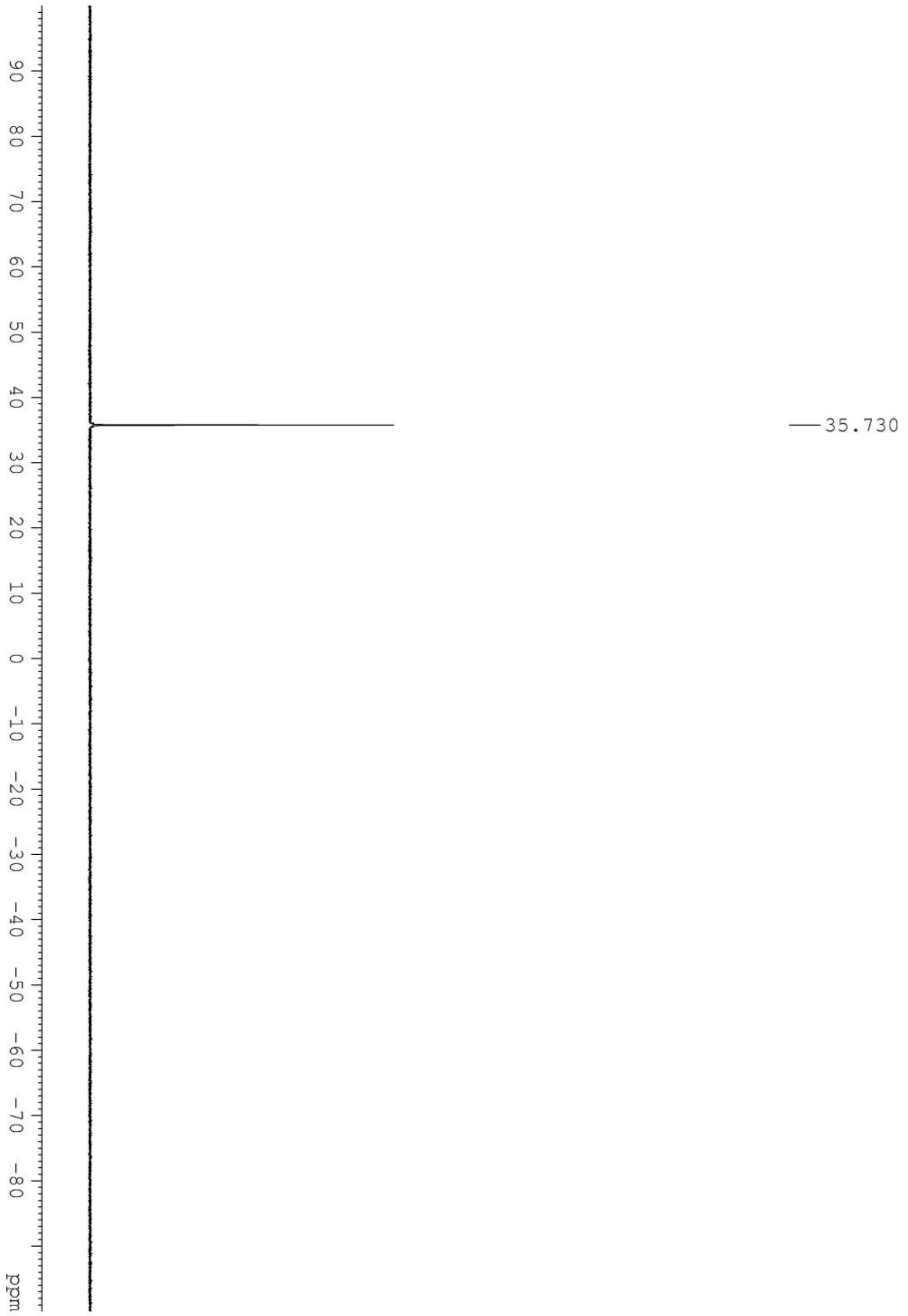
35.730
90 80 70 60 50 40 30 20 10 0 -10 -20 -30 -40 -50 -60 -70 -80
ppm

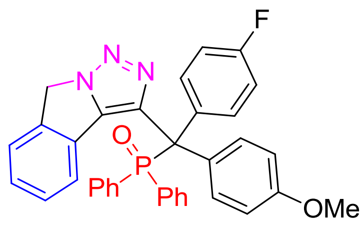
N
N
N
F
O
P
Ph
Ph
OMe

3f

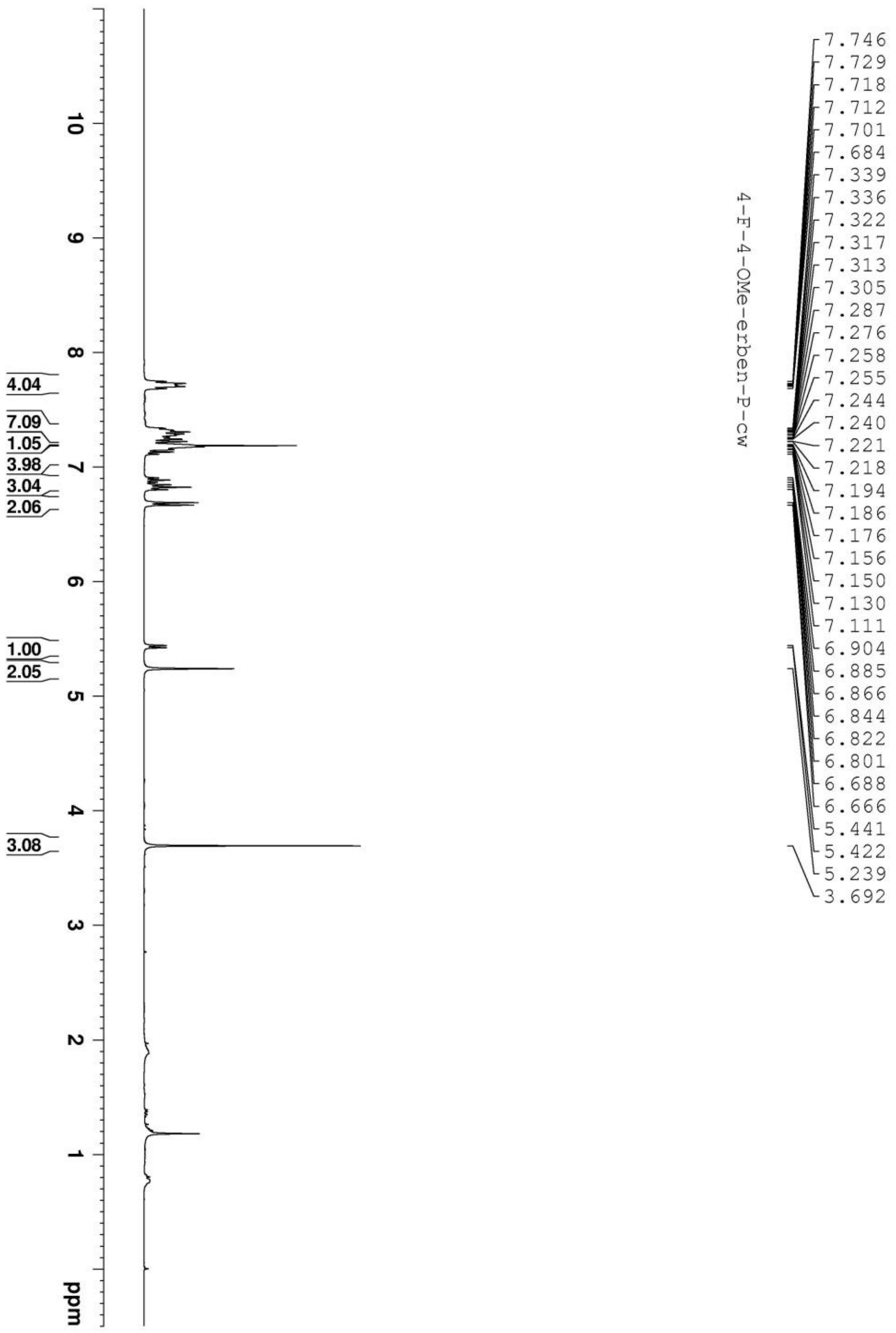
4-F-4-OMe-erben-P-cw
7.746
7.729
7.718
7.712
7.701
7.684
7.339
7.336
7.322
7.317
7.313
7.305
7.287
7.276
7.258
7.255
7.244
7.240
7.221
7.218
7.194
7.186
7.176
7.156
7.150
7.130
7.111
6.904
6.885
6.866
6.844
6.822
6.801
6.688
6.666
5.441
5.422
5.239
3.692
4.04
7.09
1.05
3.98
3.04
2.06
1.00
2.05
3.08
10
9
8
7
6
5
4
3
2
1
ppm

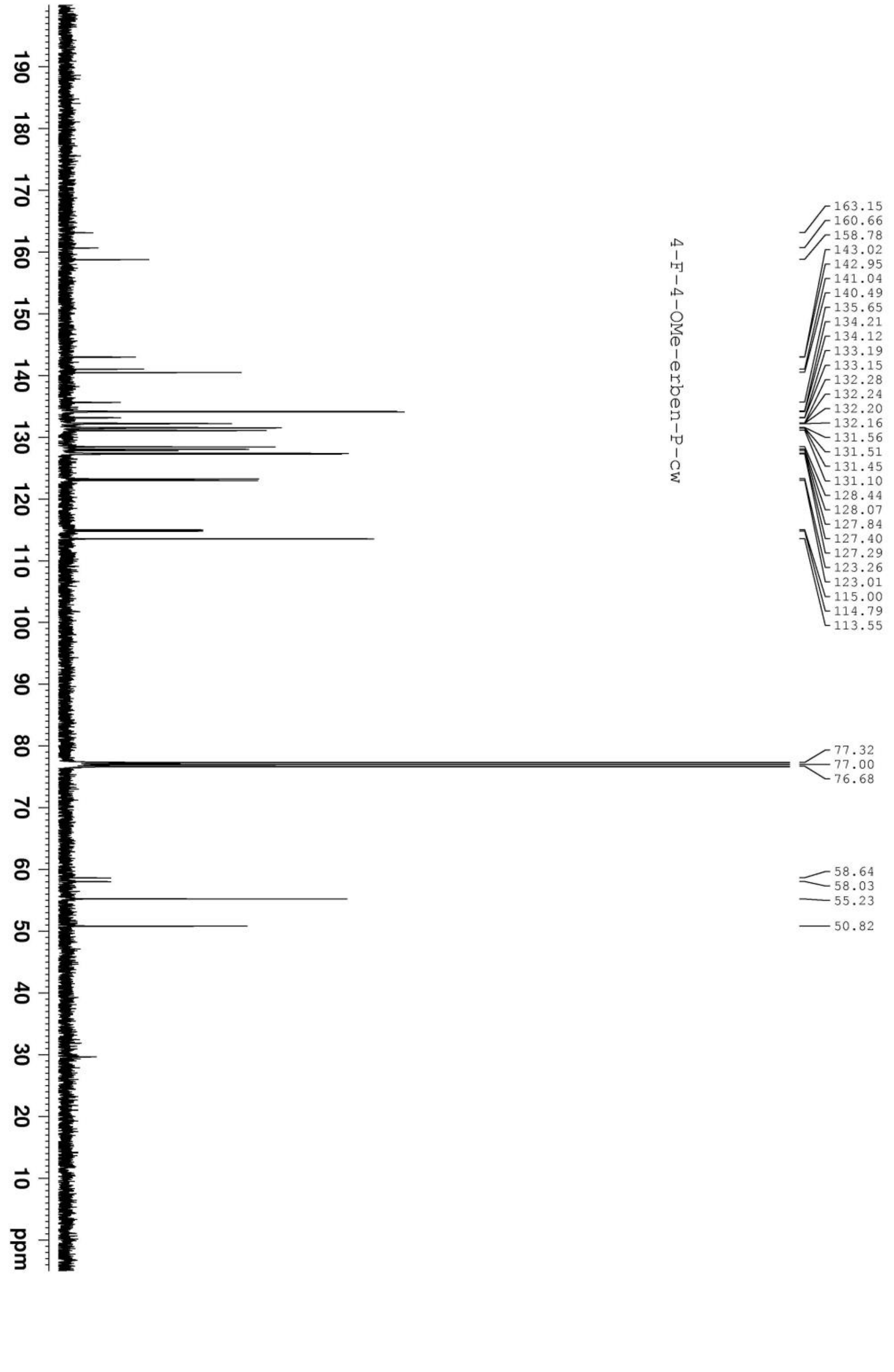
4-F-4-OMe-erben-P-cw
163.15
160.66
158.78
143.02
142.95
141.04
140.49
135.65
134.21
134.12
133.19
133.15
132.28
132.24
132.20
132.16
131.56
131.51
131.45
131.10
128.44
128.07
127.84
127.40
127.29
123.26
123.01
115.00
114.79
113.55
77.32
77.00
76.68
58.64
58.03
55.23
50.82
ppm

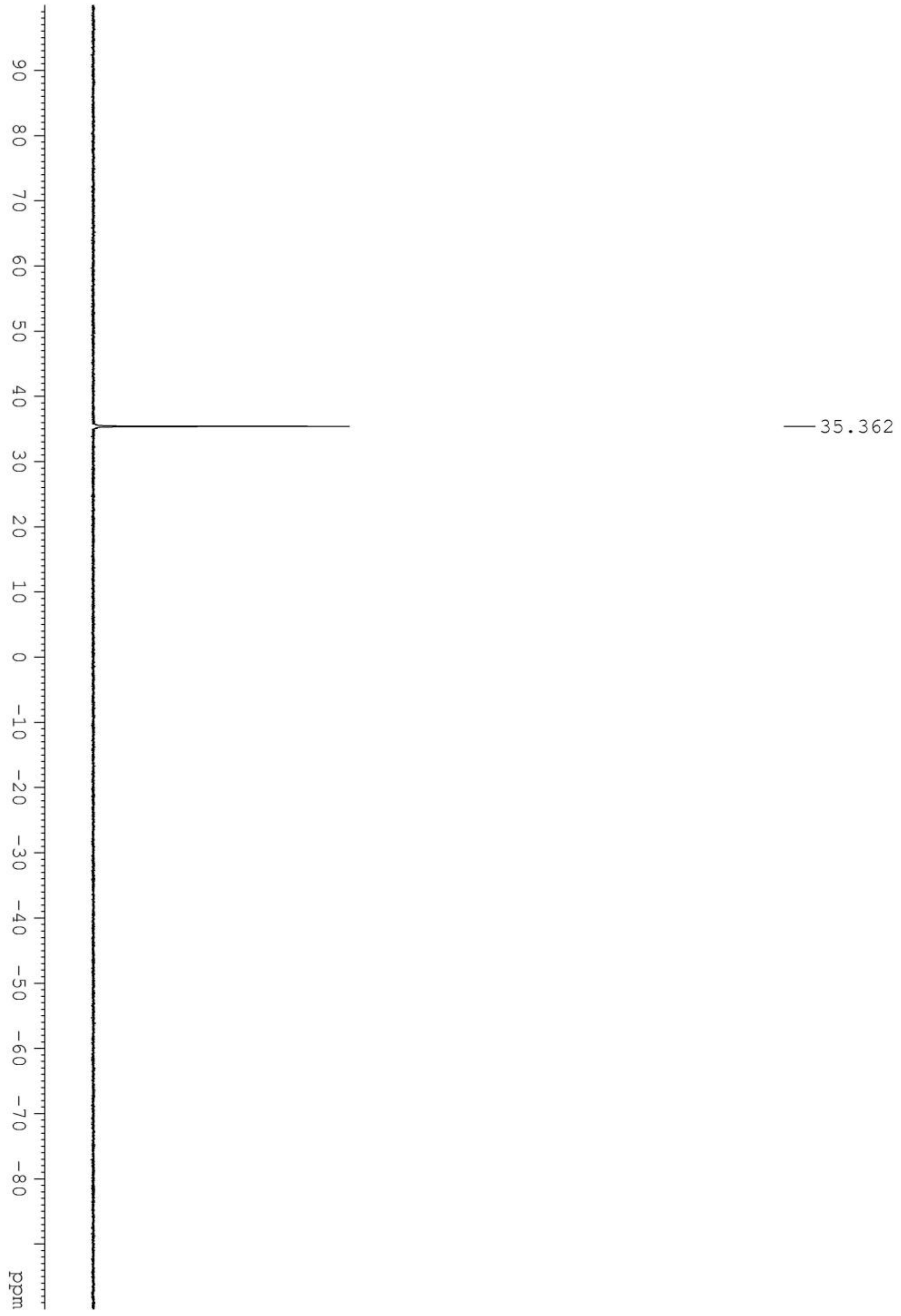
35.362
90
80
70
60
50
40
30
20
10
0
-10
-20
-30
-40
-50
-60
-70
-80
ppm

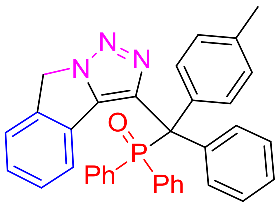
N
N
N
O
P
Ph
Ph

3g

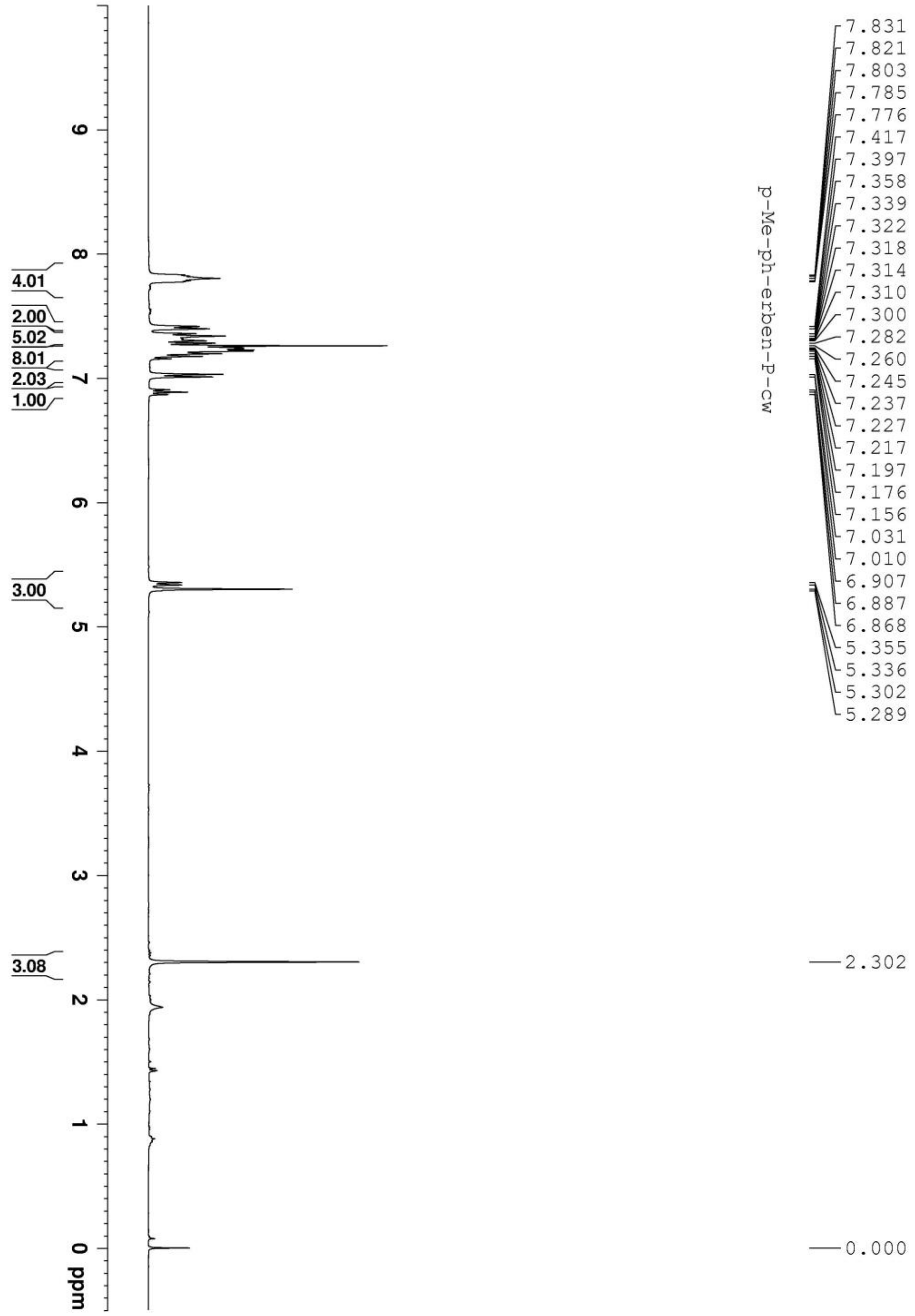
p-Me-ph-erben-P-cw
7.831
7.821
7.803
7.785
7.776
7.417
7.397
7.358
7.339
7.322
7.318
7.314
7.310
7.300
7.282
7.260
7.245
7.237
7.227
7.217
7.197
7.176
7.156
7.031
7.010
6.907
6.887
6.868
5.355
5.336
5.302
5.289
2.302
0.000
4.01
2.00
5.02
8.01
2.03
1.00
3.00
3.08
9
8
7
6
5
4
3
2
1
0 ppm

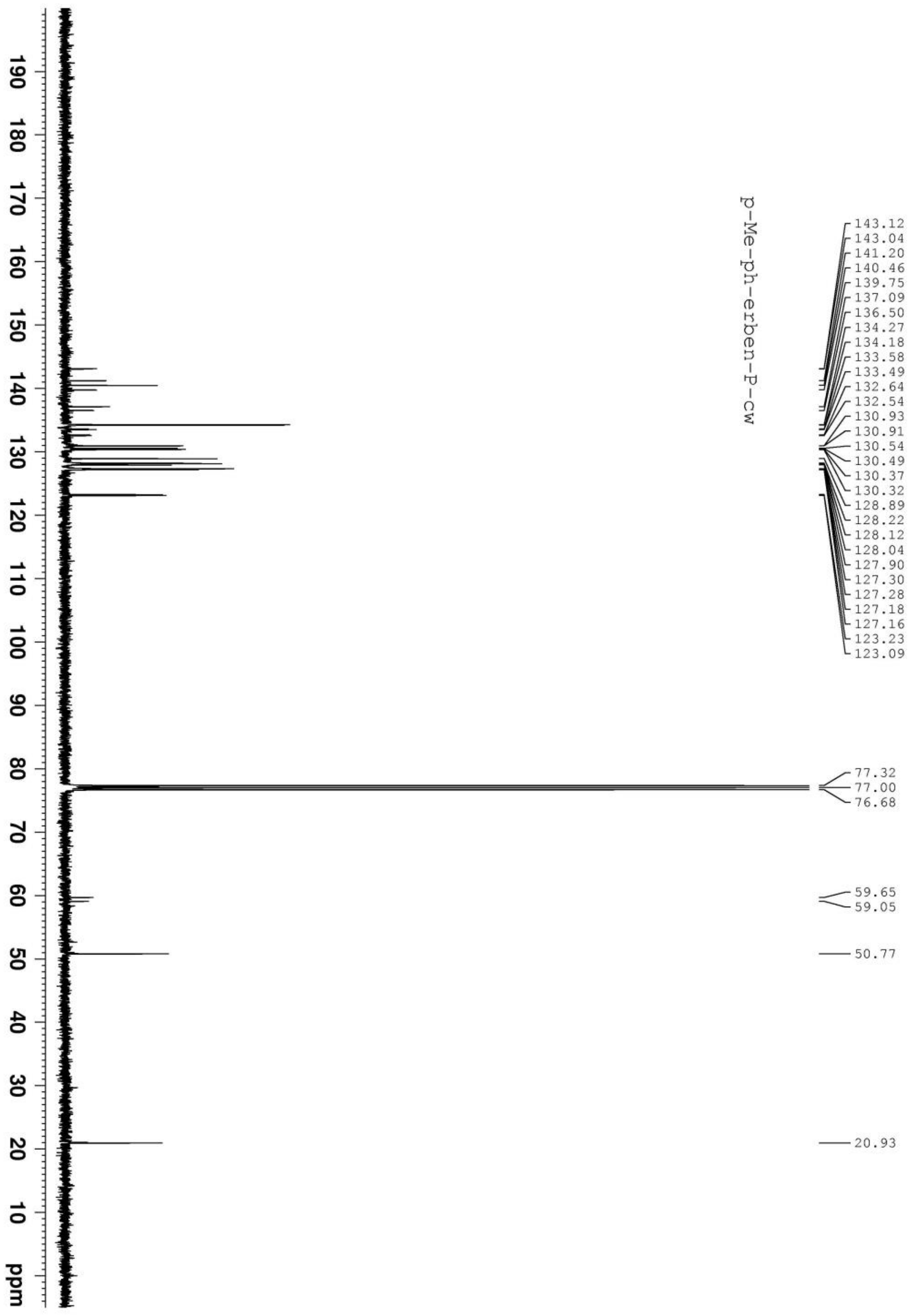
p-Me-ph-erben-P-cw
143.12
143.04
141.20
140.46
139.75
137.09
136.50
134.27
134.18
133.58
133.49
132.64
132.54
130.93
130.91
130.54
130.49
130.37
130.32
128.89
128.22
128.12
128.04
127.90
127.30
127.28
127.18
127.16
123.23
123.09
77.32
77.00
76.68
59.65
59.05
50.77
20.93
190 180 170 160 150 140 130 120 110 100 90 80 70 60 50 40 30 20 10 ppm

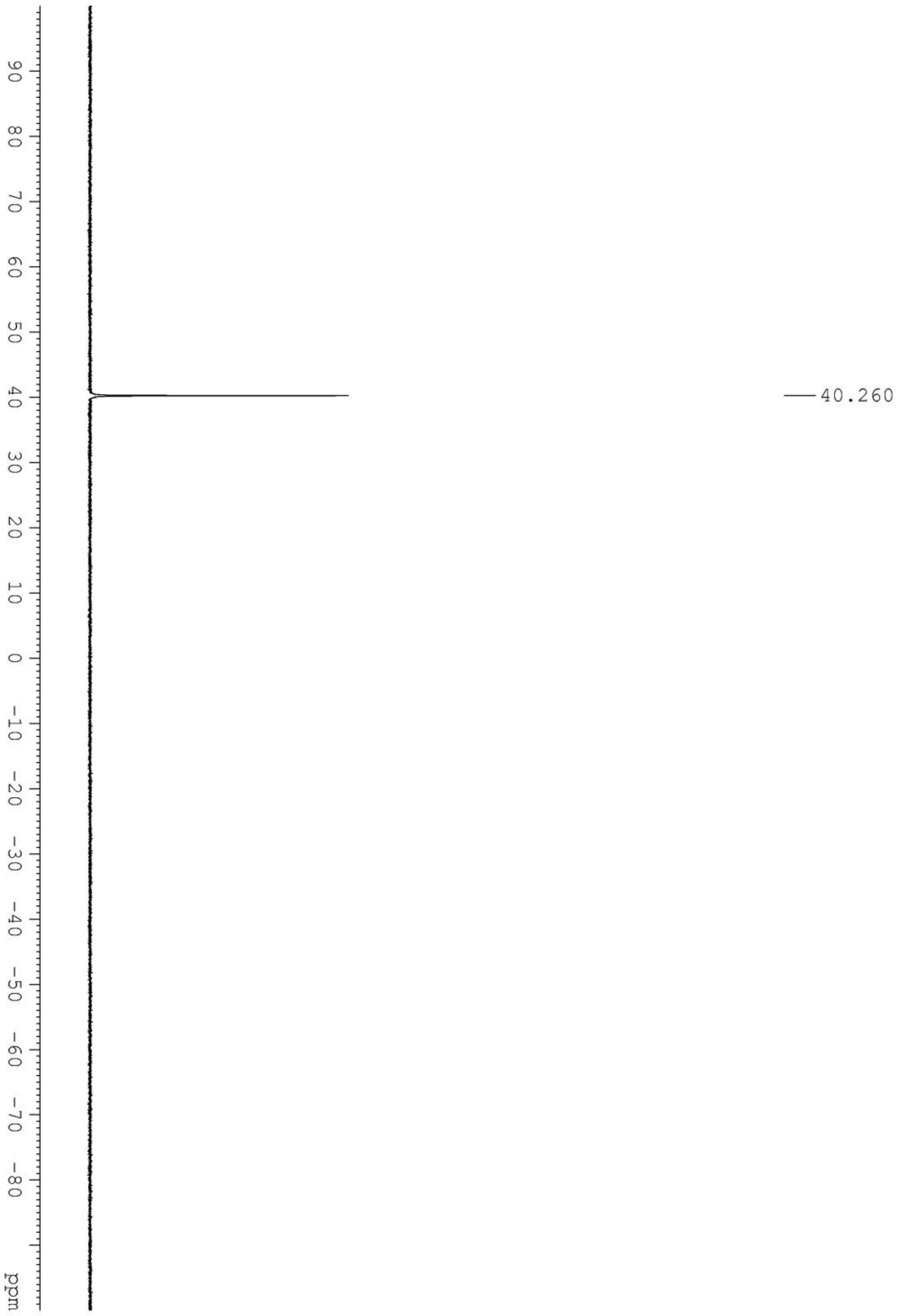
40.260
ppm

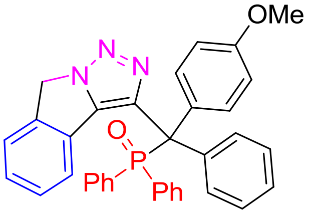
OMe
N
N
N
O
P
Ph
Ph

3h

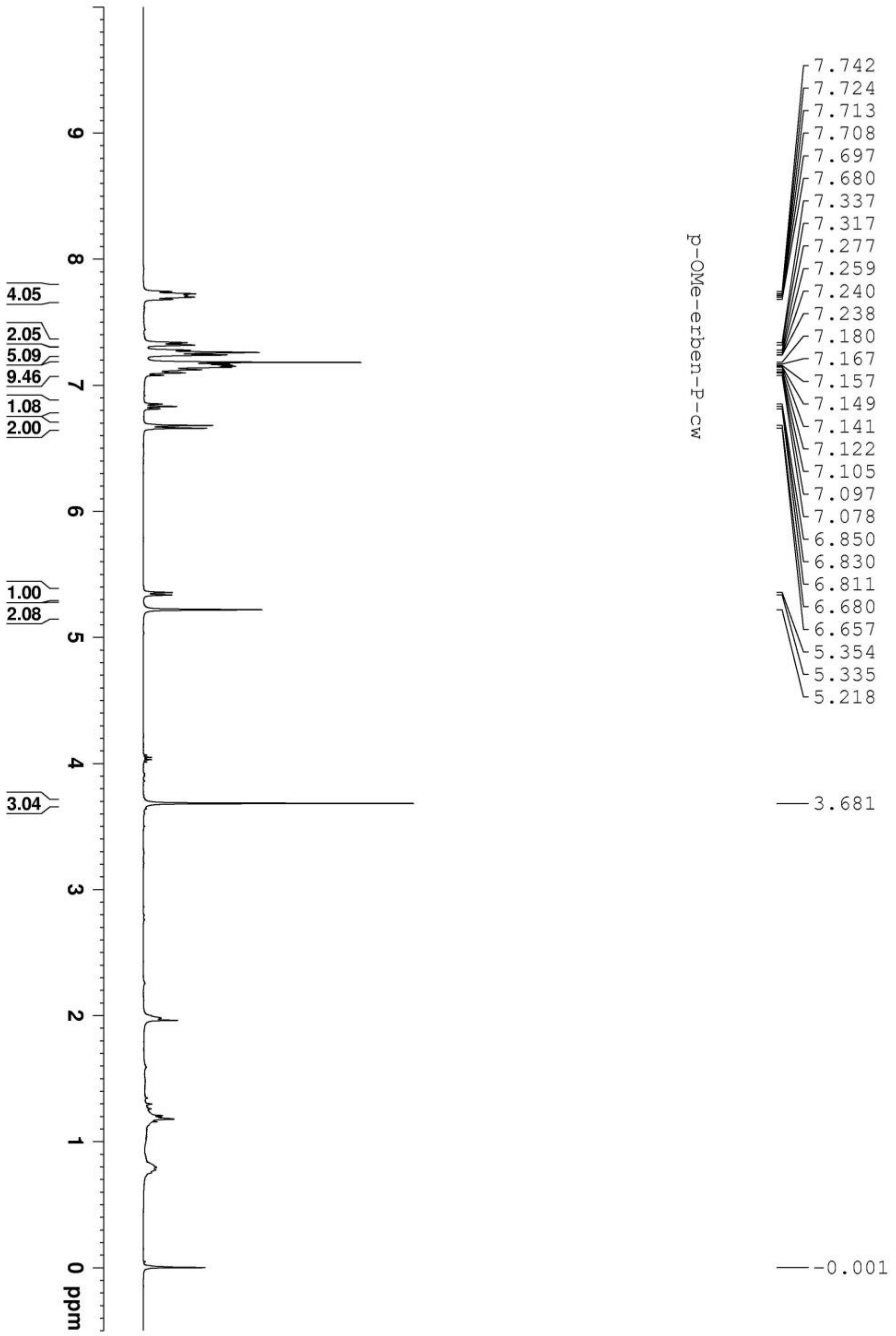
p-OMe-erben-P-cw
7.742
7.724
7.713
7.708
7.697
7.680
7.337
7.317
7.277
7.259
7.240
7.238
7.180
7.167
7.157
7.149
7.141
7.122
7.105
7.097
7.078
6.850
6.830
6.811
6.680
6.657
5.354
5.335
5.218
3.681
-0.001
4.05
2.05
5.09
9.46
1.08
2.00
1.00
2.08
3.04
ppm

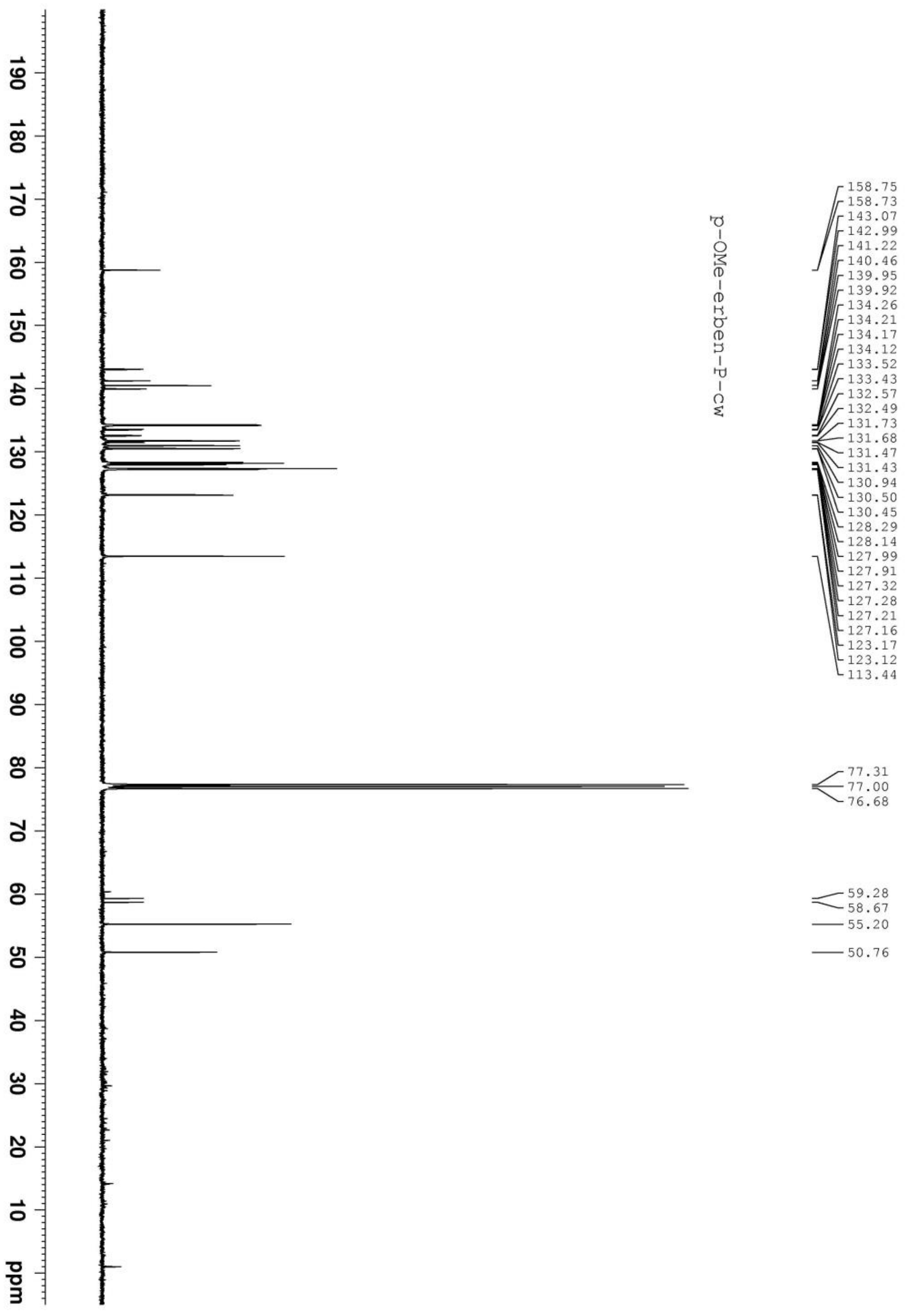
p-OMe-erben-P-cw
158.75
158.73
143.07
142.99
141.22
140.46
139.95
139.92
134.26
134.21
134.17
134.12
133.52
133.43
132.57
132.49
131.73
131.68
131.47
131.43
130.94
130.50
130.45
128.29
128.14
127.99
127.91
127.32
127.28
127.21
127.16
123.17
123.12
113.44
77.31
77.00
76.68
59.28
58.67
55.20
50.76
190
180
170
160
150
140
130
120
110
100
90
80
70
60
50
40
30
20
10
ppm

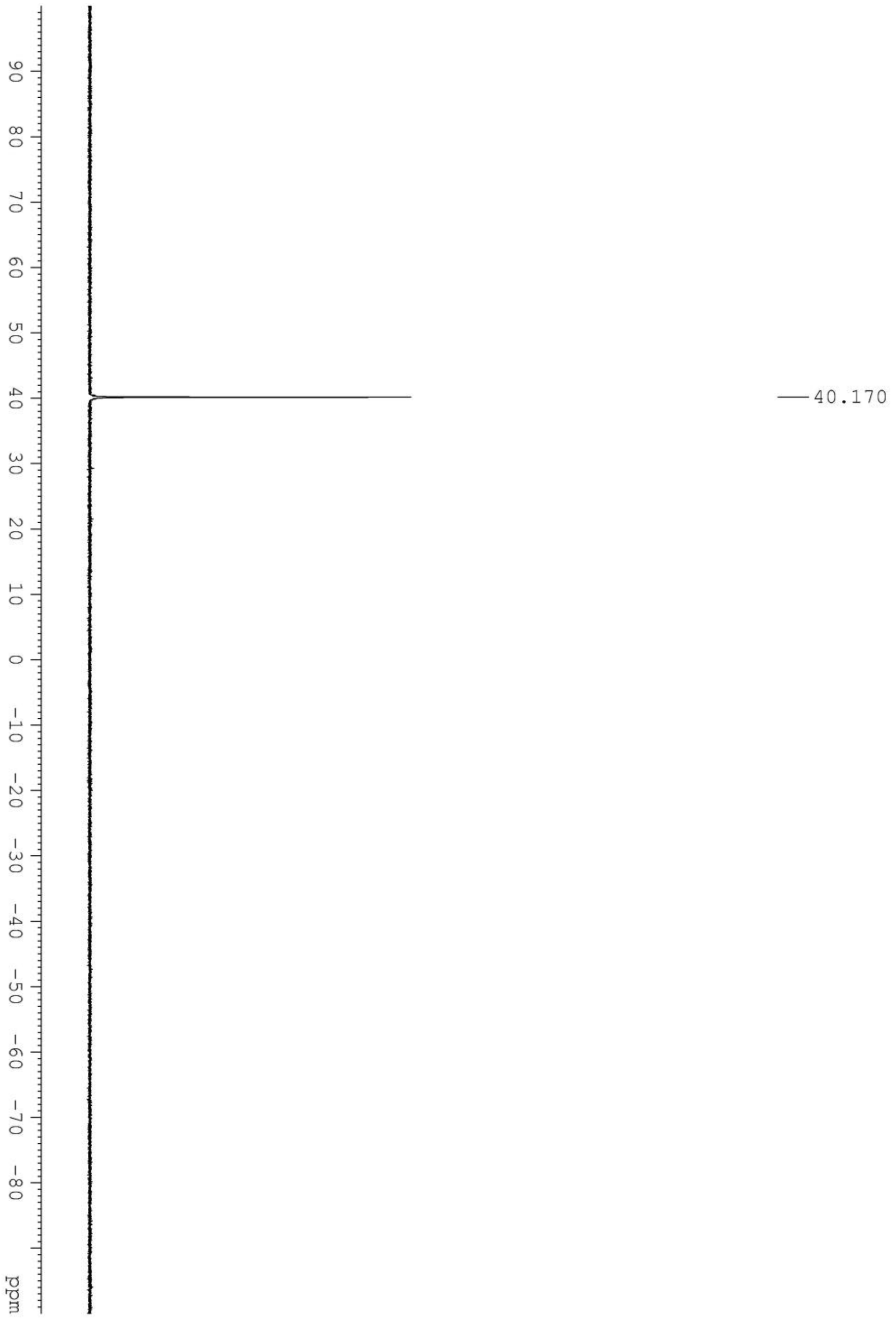
40.170
ppm

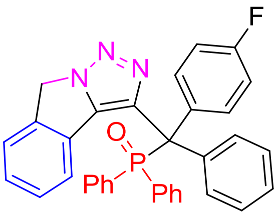
F
N
N
N
O
P
Ph
Ph

3i

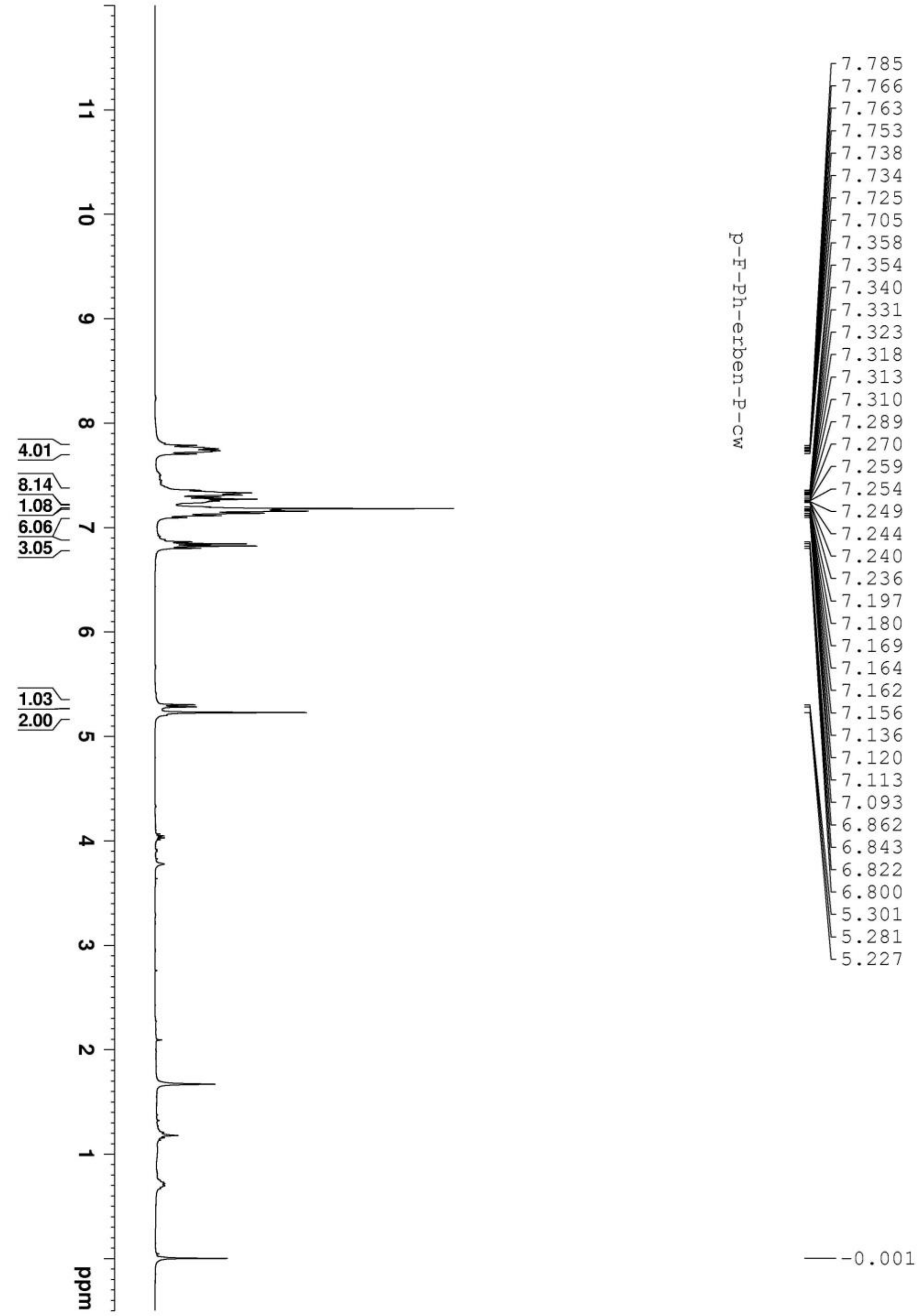
p-F-Ph-erben-P-cw
7.785
7.766
7.763
7.753
7.738
7.734
7.725
7.705
7.358
7.354
7.340
7.331
7.323
7.318
7.313
7.310
7.289
7.270
7.259
7.254
7.249
7.244
7.240
7.236
7.197
7.180
7.169
7.164
7.162
7.156
7.136
7.120
7.113
7.093
6.862
6.843
6.822
6.800
5.301
5.281
5.227
-0.001
4.01
8.14
1.08
6.06
3.05
1.03
2.00
ppm

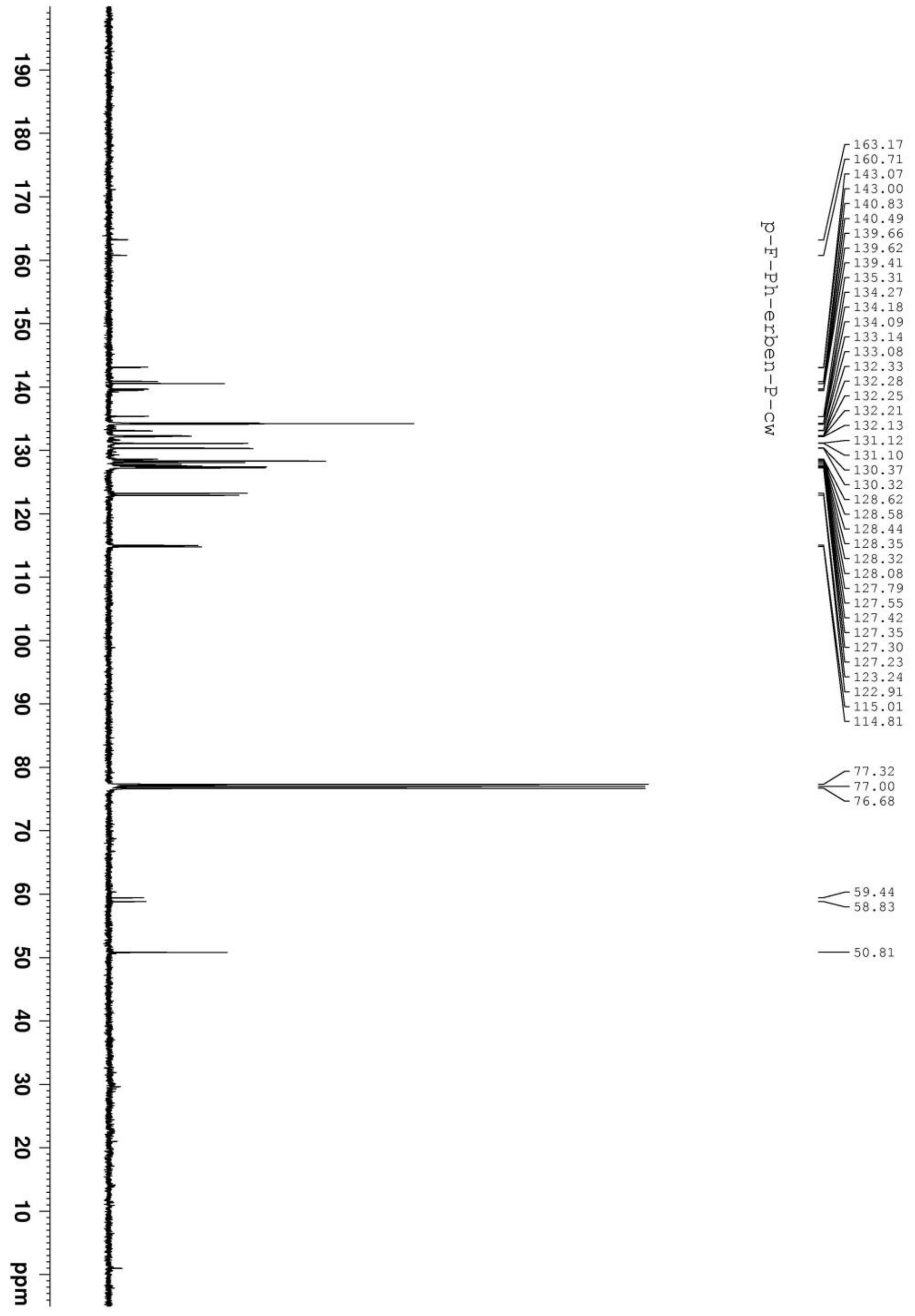
p-F-Ph-erben-P-cw
163.17
160.71
143.07
143.00
140.83
140.49
139.66
139.62
139.41
135.31
134.27
134.18
134.09
133.14
133.08
132.33
132.28
132.25
132.21
132.13
131.12
131.10
130.37
130.32
128.62
128.58
128.44
128.35
128.32
128.08
127.79
127.55
127.42
127.35
127.30
127.23
123.24
122.91
115.01
114.81
77.32
77.00
76.68
59.44
58.83
50.81
190 180 170 160 150 140 130 120 110 100 90 80 70 60 50 40 30 20 10 ppm

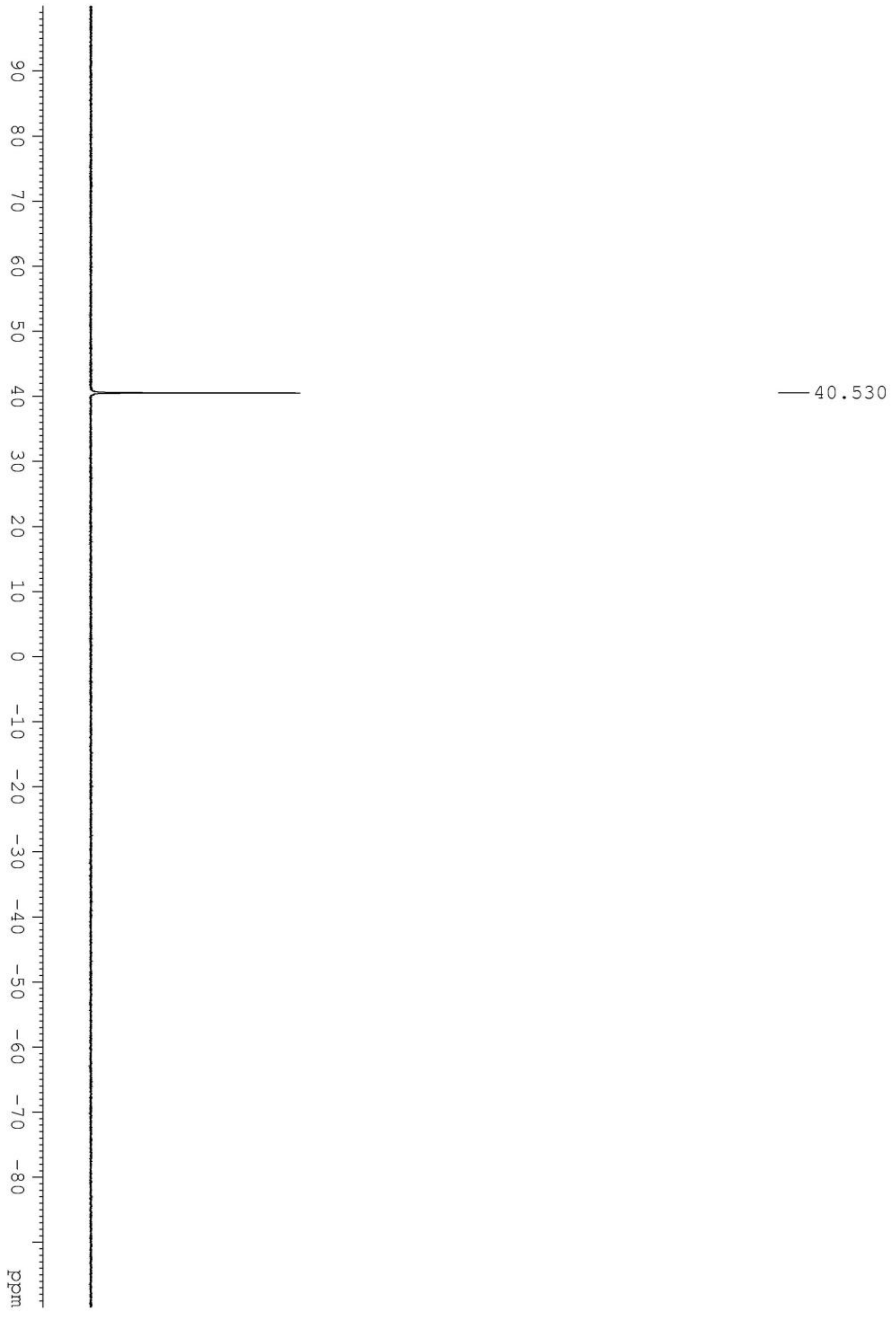
40.530
90
80
70
60
50
40
30
20
10
0
-10
-20
-30
-40
-50
-60
-70
-80
ppm

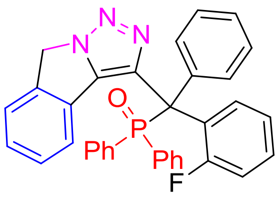
N
N
N
O
P
Ph
Ph
F

3j

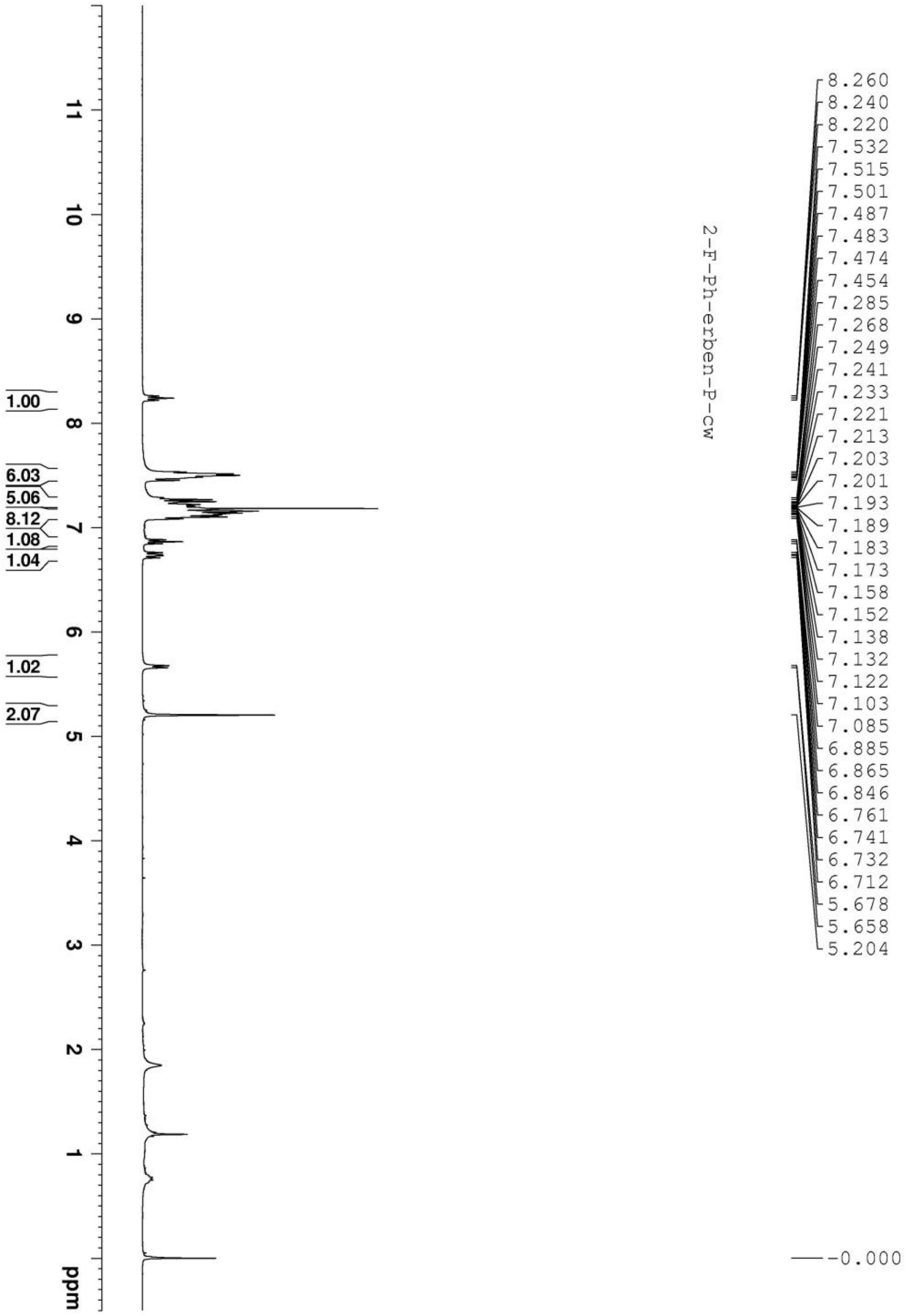
2-F-Ph-erben-P-cw
8.260
8.240
8.220
7.532
7.515
7.501
7.487
7.483
7.474
7.454
7.285
7.268
7.249
7.241
7.233
7.221
7.213
7.203
7.201
7.193
7.189
7.183
7.173
7.158
7.152
7.138
7.132
7.122
7.103
7.085
6.885
6.865
6.846
6.761
6.741
6.732
6.712
5.678
5.658
5.204
-0.000
1.00
6.03
5.06
8.12
1.08
1.04
1.02
2.07
ppm

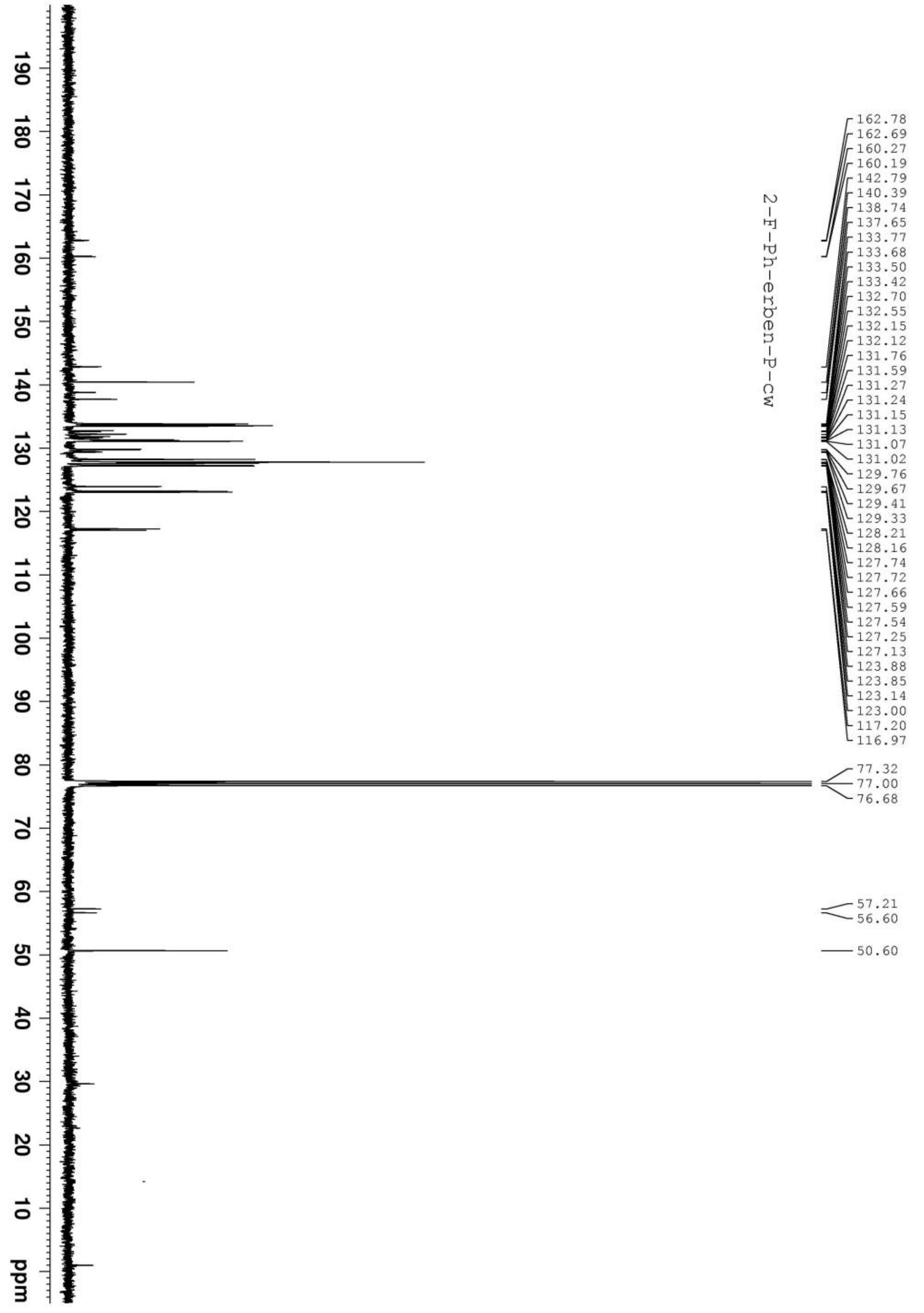
2-F-Ph-erben-P-cw
162.78
162.69
160.27
160.19
142.79
140.39
138.74
137.65
133.77
133.68
133.50
133.42
132.70
132.55
132.15
132.12
131.76
131.59
131.27
131.24
131.15
131.13
131.07
131.02
129.76
129.67
129.41
129.33
128.21
128.16
127.74
127.72
127.66
127.59
127.54
127.25
127.13
123.88
123.85
123.14
123.00
117.20
116.97
77.32
77.00
76.68
57.21
56.60
50.60
ppm

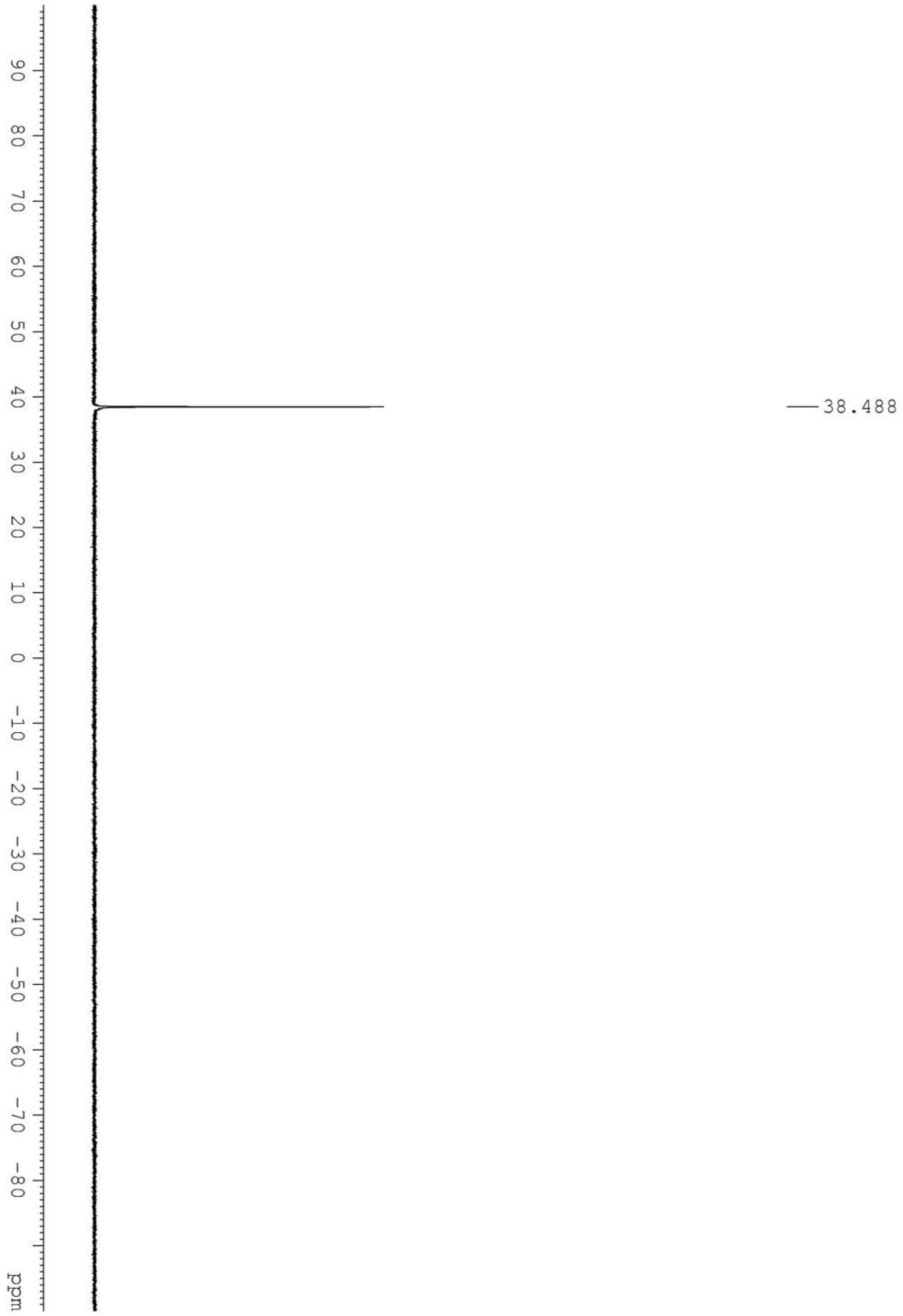
38.488
ppm

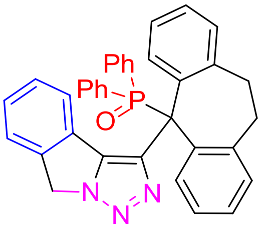
Ph
Ph
P
O
N
N
N

3k

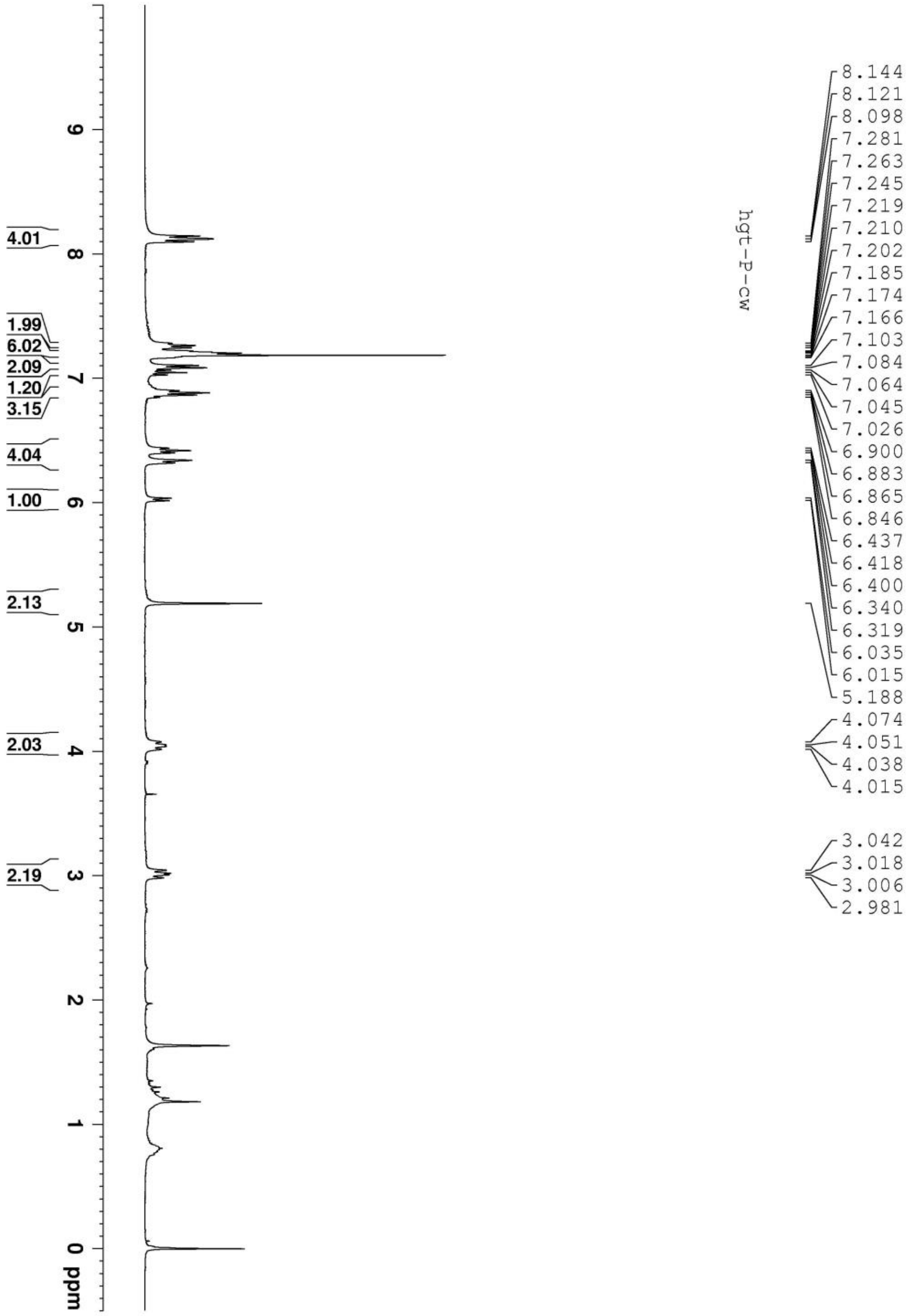
8.144
8.121
8.098
7.281
7.263
7.245
7.219
7.210
7.202
7.185
7.174
7.166
7.103
7.084
7.064
7.045
7.026
6.900
6.883
6.865
6.846
6.437
6.418
6.400
6.340
6.319
6.035
6.015
5.188
4.074
4.051
4.038
4.015
3.042
3.018
3.006
2.981
hgt-P-cw
4.01
1.99
6.02
2.09
1.20
3.15
4.04
1.00
2.13
2.03
2.19
9
8
7
6
5
4
3
2
1
0 ppm

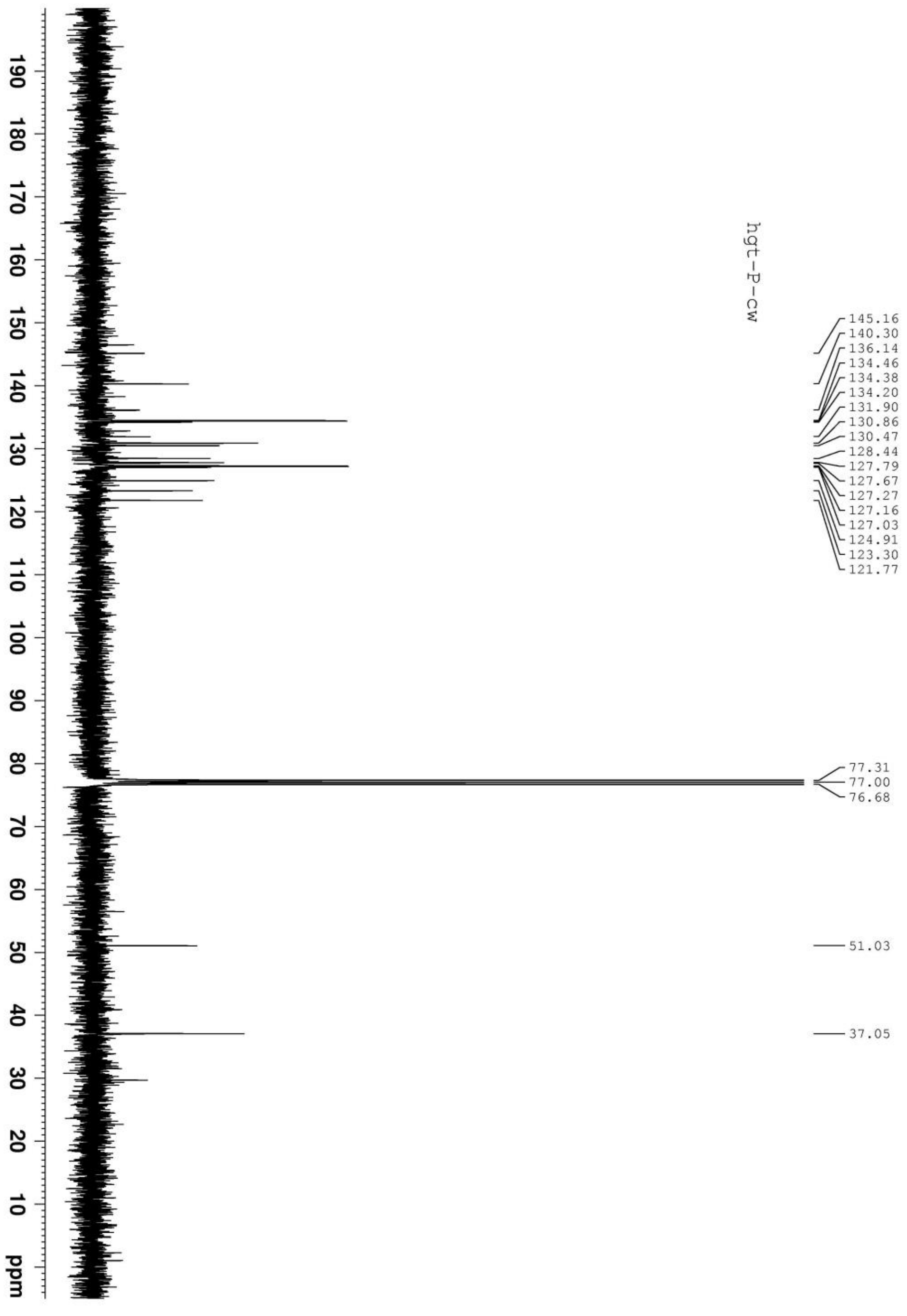
hgt-P-cw
145.16
140.30
136.14
134.46
134.38
134.20
131.90
130.86
130.47
128.44
127.79
127.67
127.27
127.16
127.03
124.91
123.30
121.77
77.31
77.00
76.68
51.03
37.05
ppm

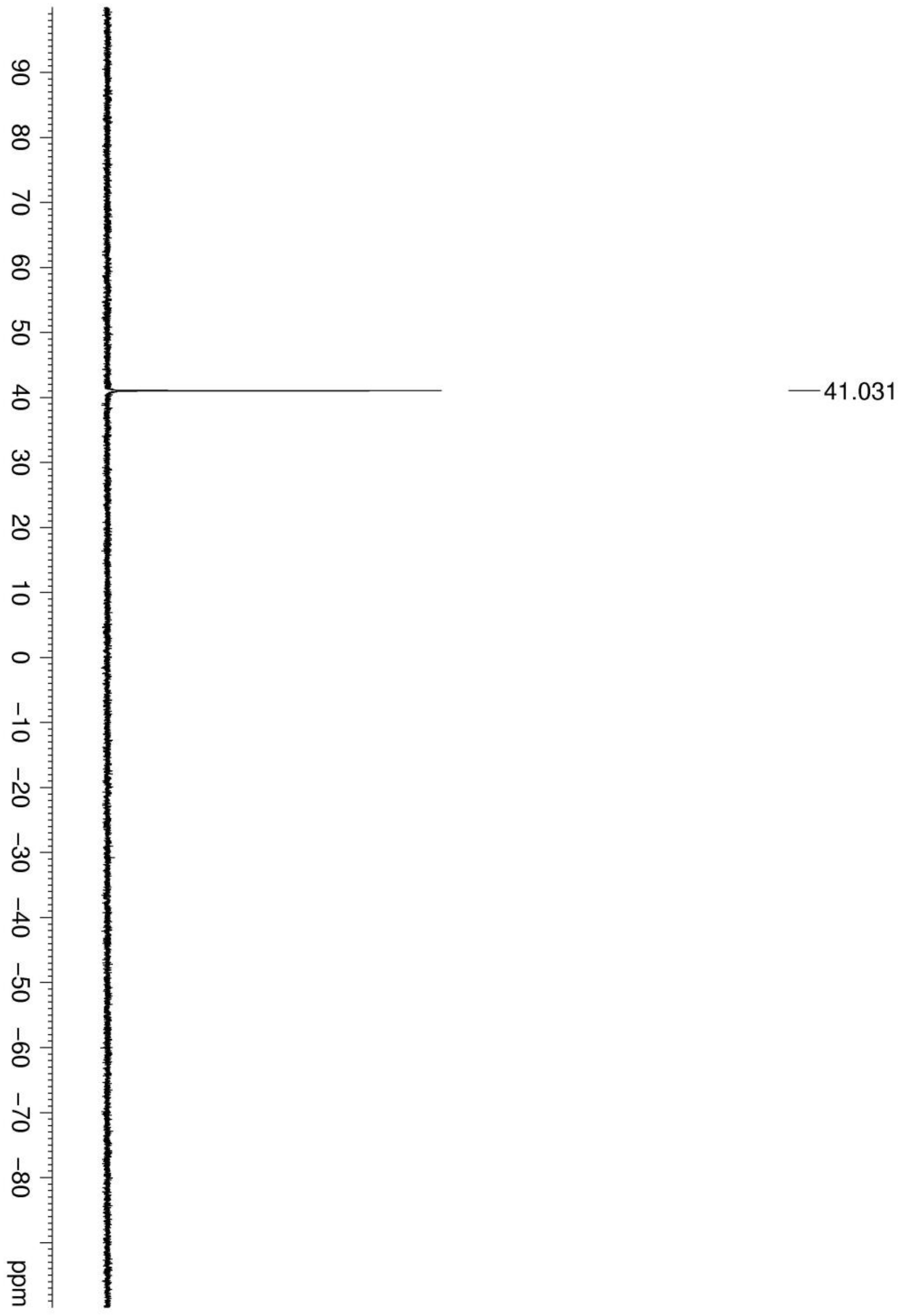
41.031
ppm
90
80
70
60
50
40
30
20
10
0
−10
−20
−30
−40
−50
−60
−70
−80

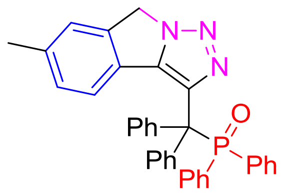
N
N
N
Ph
Ph
O
P
Ph
Ph

3l

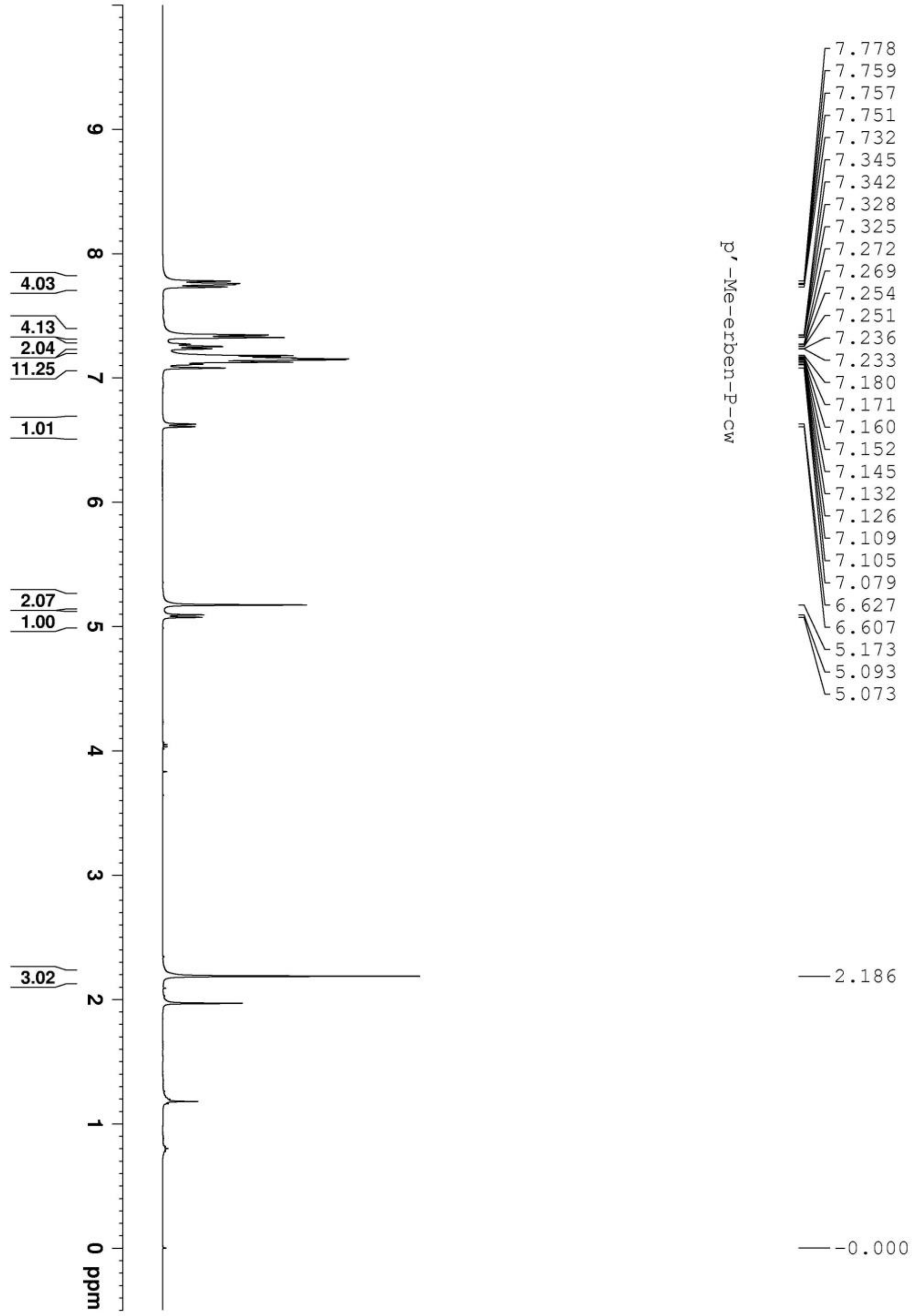
p'-Me-erben-P-cw
7.778
7.759
7.757
7.751
7.732
7.345
7.342
7.328
7.325
7.272
7.269
7.254
7.251
7.236
7.233
7.180
7.171
7.160
7.152
7.145
7.132
7.126
7.109
7.105
7.079
6.627
6.607
5.173
5.093
5.073
2.186
-0.000
4.03
4.13
2.04
11.25
1.01
2.07
1.00
3.02
9
8
7
6
5
4
3
2
1
0 ppm

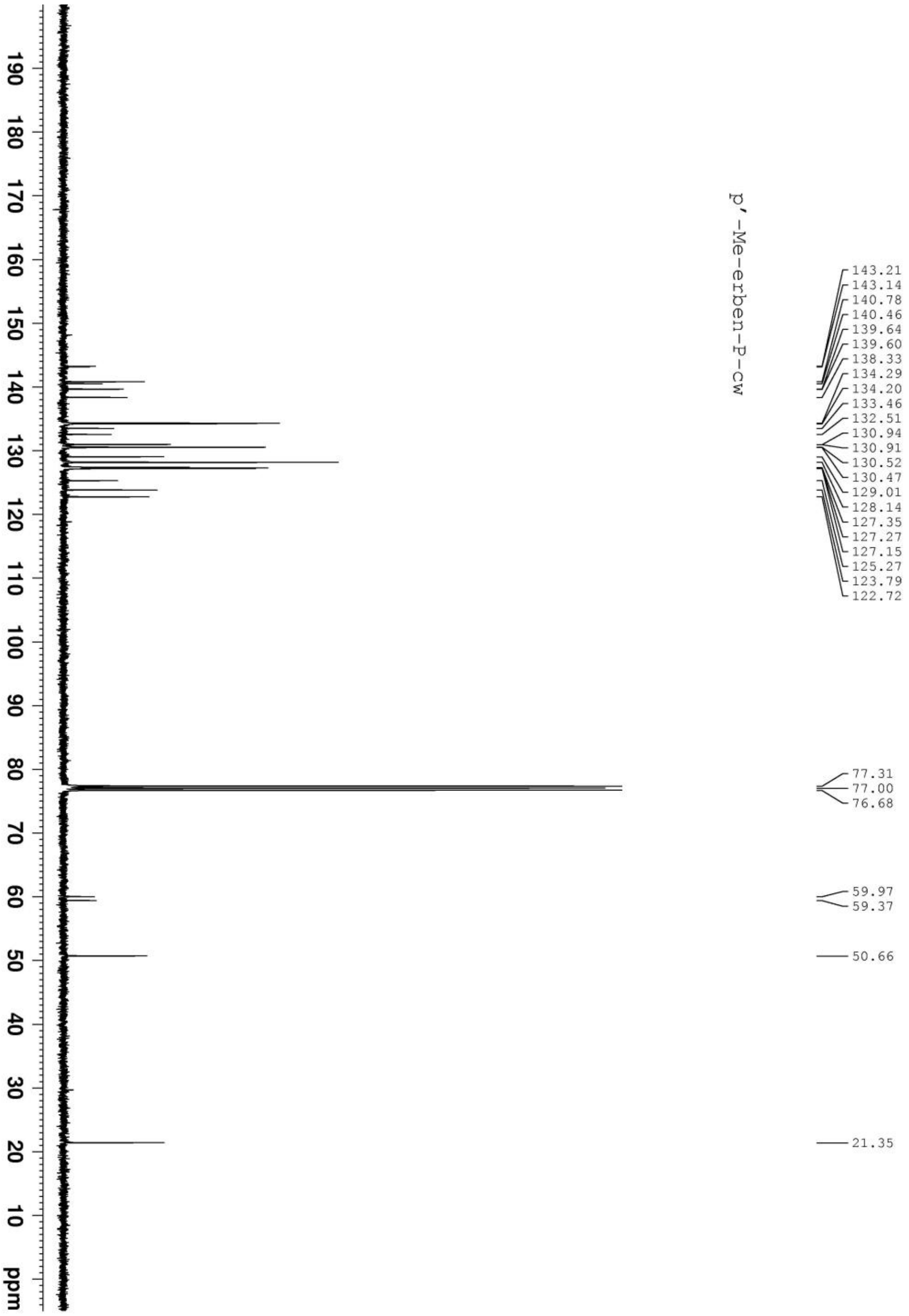
p'-Me-erben-P-cw
143.21
143.14
140.78
140.46
139.64
139.60
138.33
134.29
134.20
133.46
132.51
130.94
130.91
130.52
130.47
129.01
128.14
127.35
127.27
127.15
125.27
123.79
122.72
77.31
77.00
76.68
59.97
59.37
50.66
21.35
190
180
170
160
150
140
130
120
110
100
90
80
70
60
50
40
30
20
10
ppm

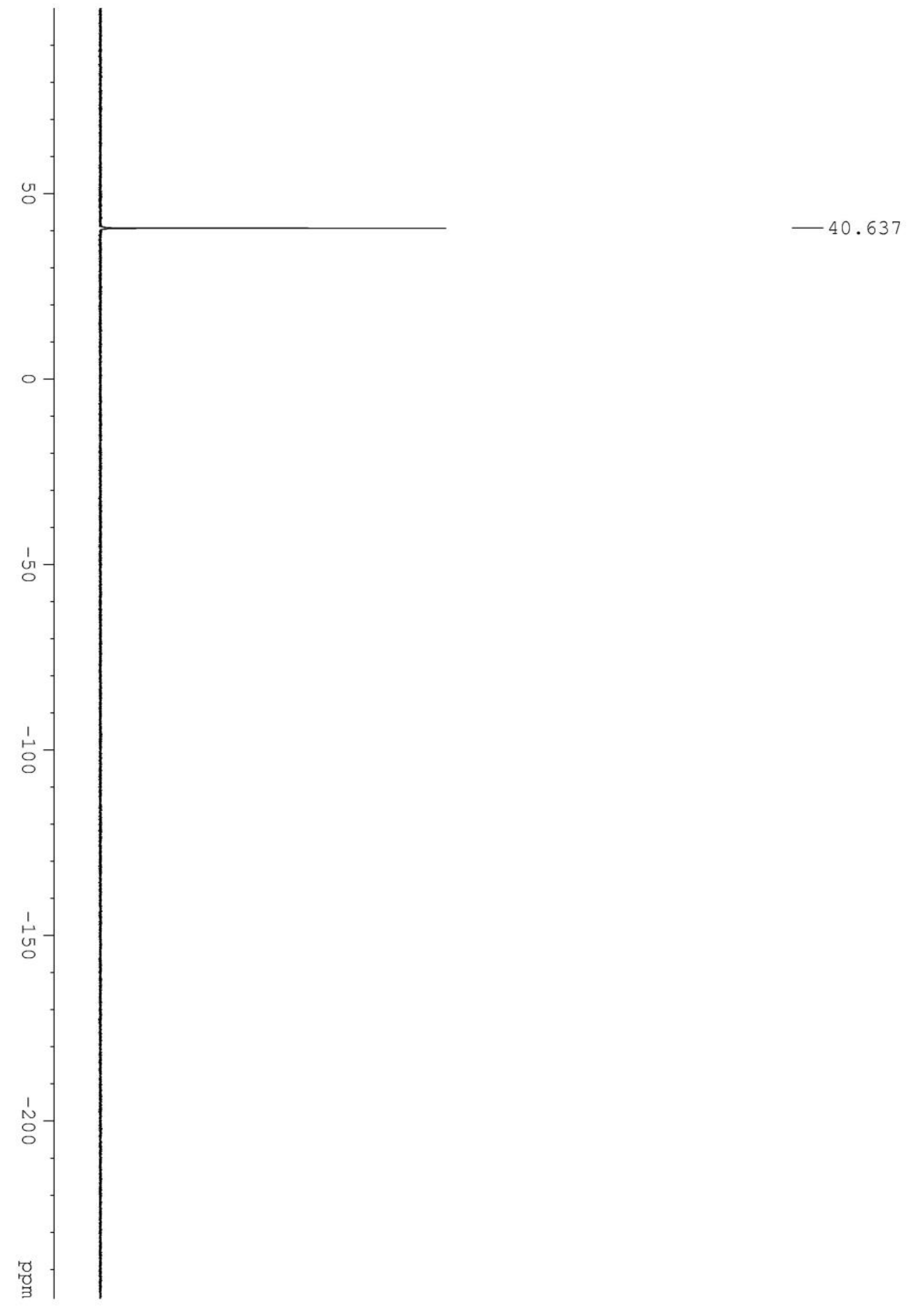
40.637
50
0
-50
-100
-150
-200
ppm

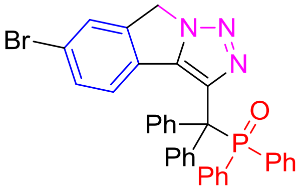
Br
N
N
N
Ph
Ph
O
P
Ph
Ph

3m

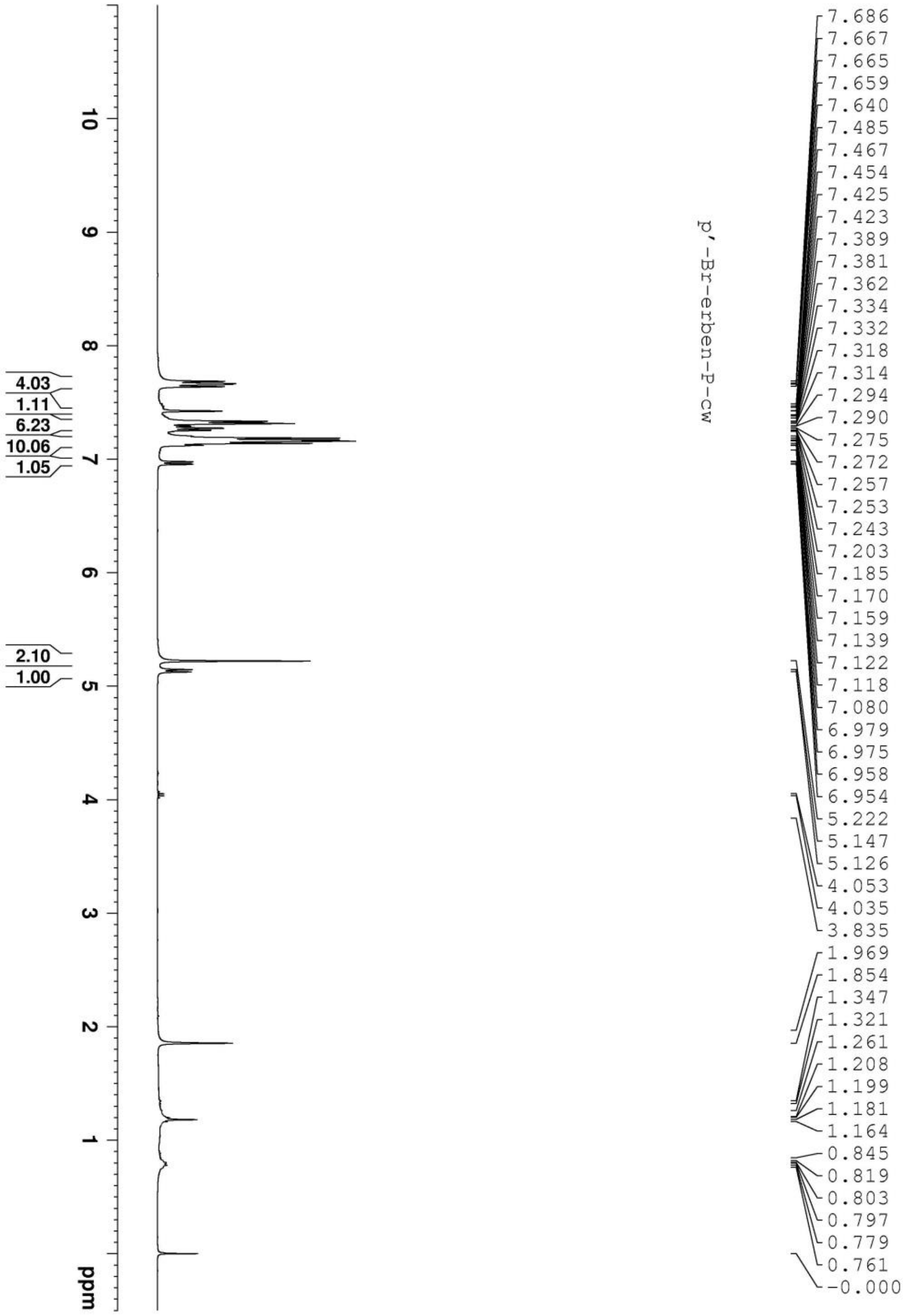
p'-Br-erben-P-cw
7.686
7.667
7.665
7.659
7.640
7.485
7.467
7.454
7.425
7.423
7.389
7.381
7.362
7.334
7.332
7.318
7.314
7.294
7.290
7.275
7.272
7.257
7.253
7.243
7.203
7.185
7.170
7.159
7.139
7.122
7.118
7.080
6.979
6.975
6.958
6.954
5.222
5.147
5.126
4.053
4.035
3.835
1.969
1.854
1.347
1.321
1.261
1.208
1.199
1.181
1.164
0.845
0.819
0.803
0.797
0.779
0.761
-0.000
4.03
1.11
6.23
10.06
1.05
2.10
1.00
ppm

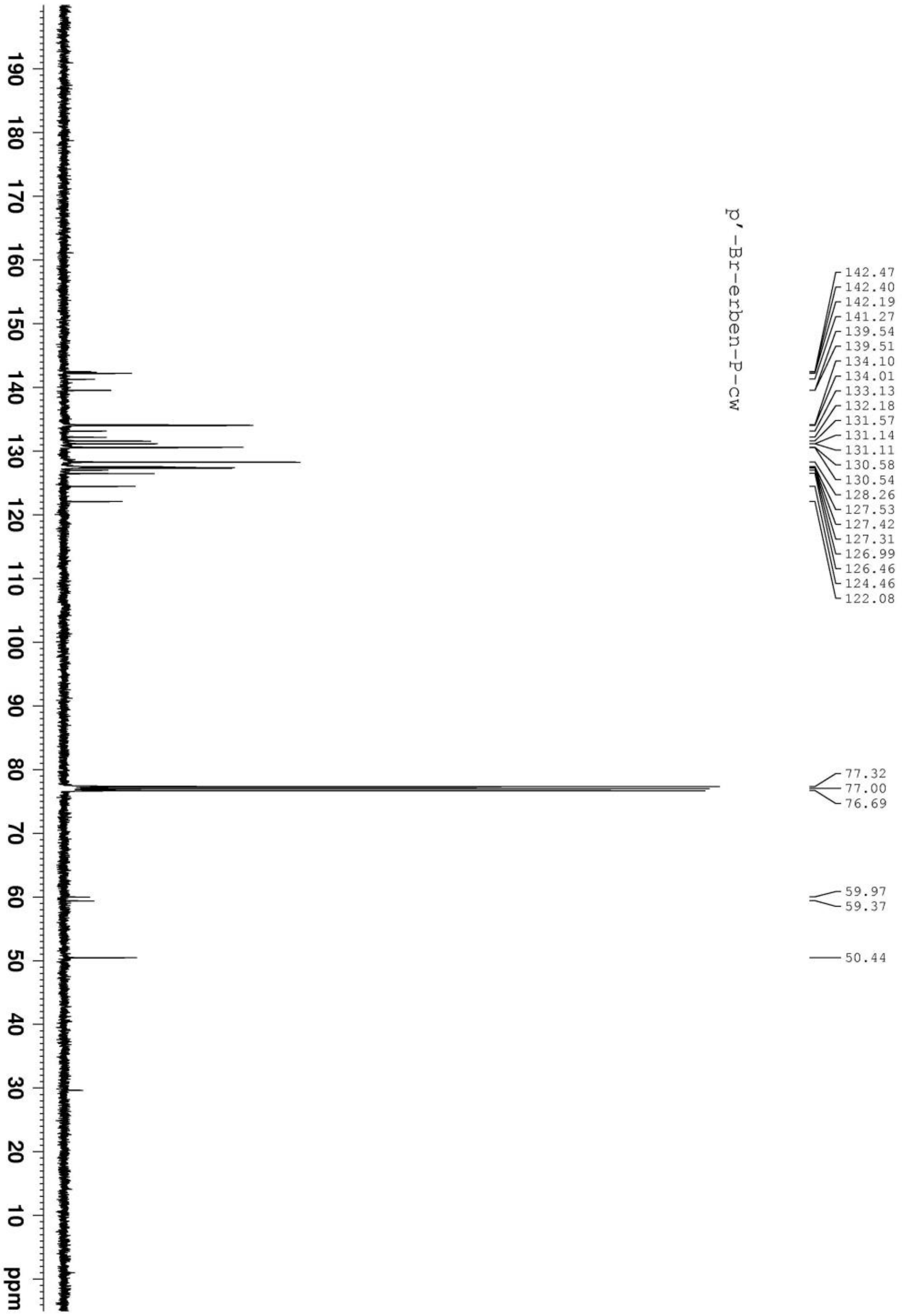
p'-Br-erben-P-cw
142.47
142.40
142.19
141.27
139.54
139.51
134.10
134.01
133.13
132.18
131.57
131.14
131.11
130.58
130.54
128.26
127.53
127.42
127.31
126.99
126.46
124.46
122.08
77.32
77.00
76.69
59.97
59.37
50.44
190
180
170
160
150
140
130
120
110
100
90
80
70
60
50
40
30
20
10
ppm

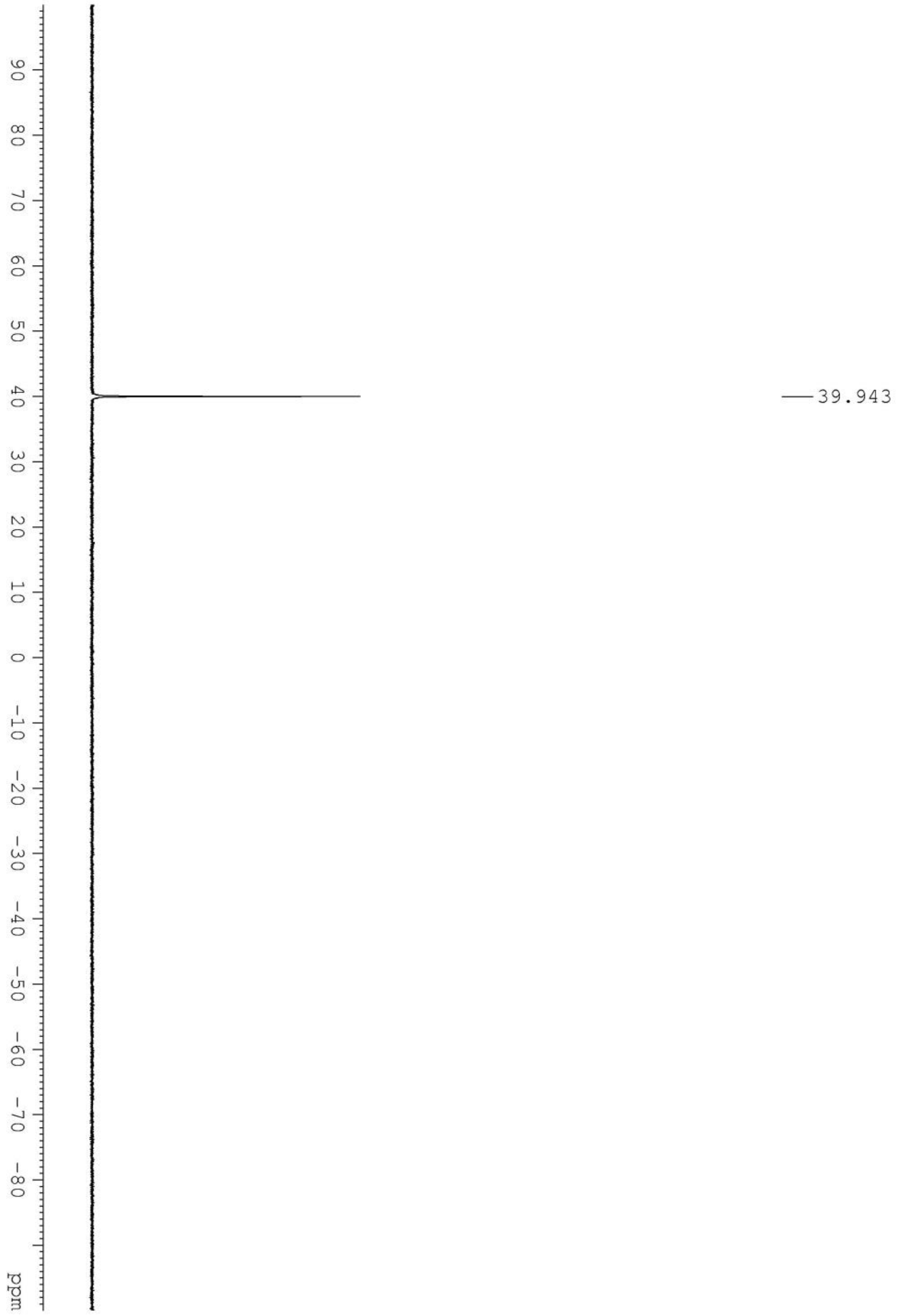
39.943
90
80
70
60
50
40
30
20
10
0
-10
-20
-30
-40
-50
-60
-70
-80
ppm

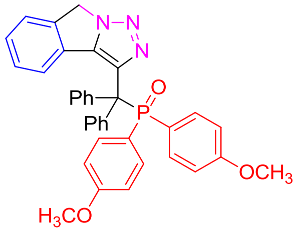
N
N
N
Ph
Ph
P
O
OCH3
H3CO

3p

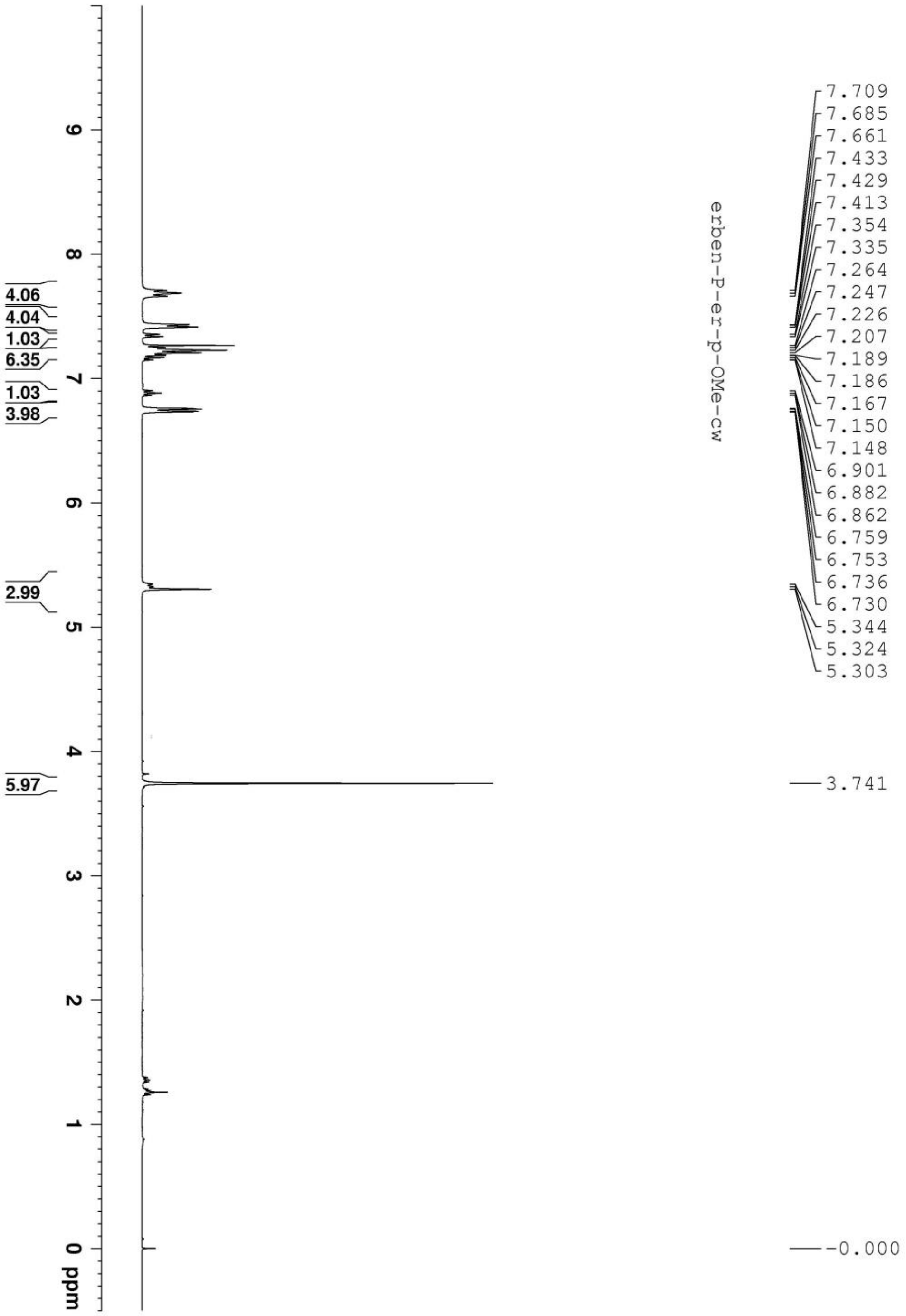
erben-P-er-p-OMe-cw
7.709
7.685
7.661
7.433
7.429
7.413
7.354
7.335
7.264
7.247
7.226
7.207
7.189
7.186
7.167
7.150
7.148
6.901
6.882
6.862
6.759
6.753
6.736
6.730
5.344
5.324
5.303
3.741
-0.000
4.06
4.04
1.03
6.35
1.03
3.98
2.99
5.97
ppm

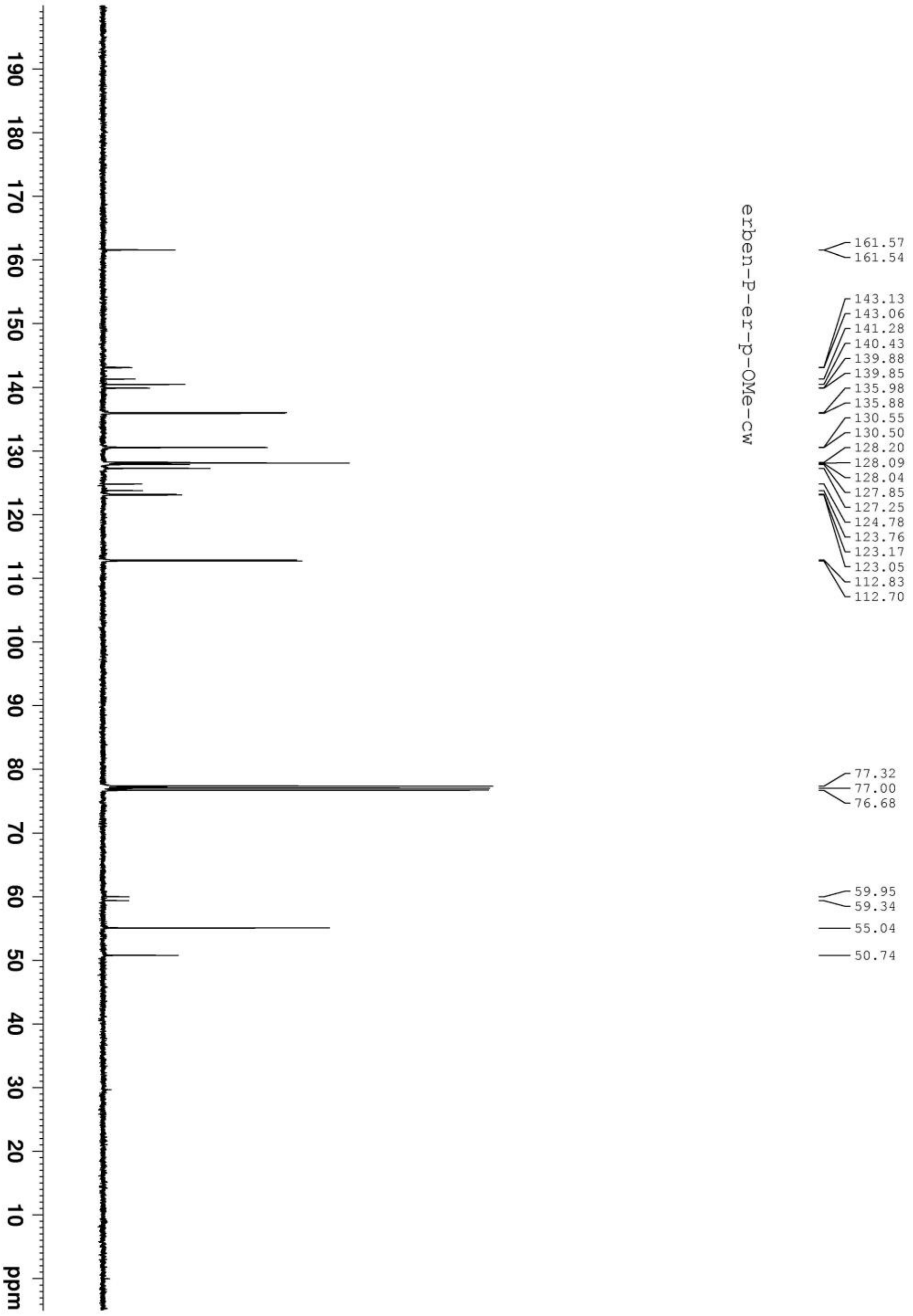
erben-P-er-p-OMe-cw
161.57
161.54
143.13
143.06
141.28
140.43
139.88
139.85
135.98
135.88
130.55
130.50
128.20
128.09
128.04
127.85
127.25
124.78
123.76
123.17
123.05
112.83
112.70
77.32
77.00
76.68
59.95
59.34
55.04
50.74
190 180 170 160 150 140 130 120 110 100 90 80 70 60 50 40 30 20 10 ppm

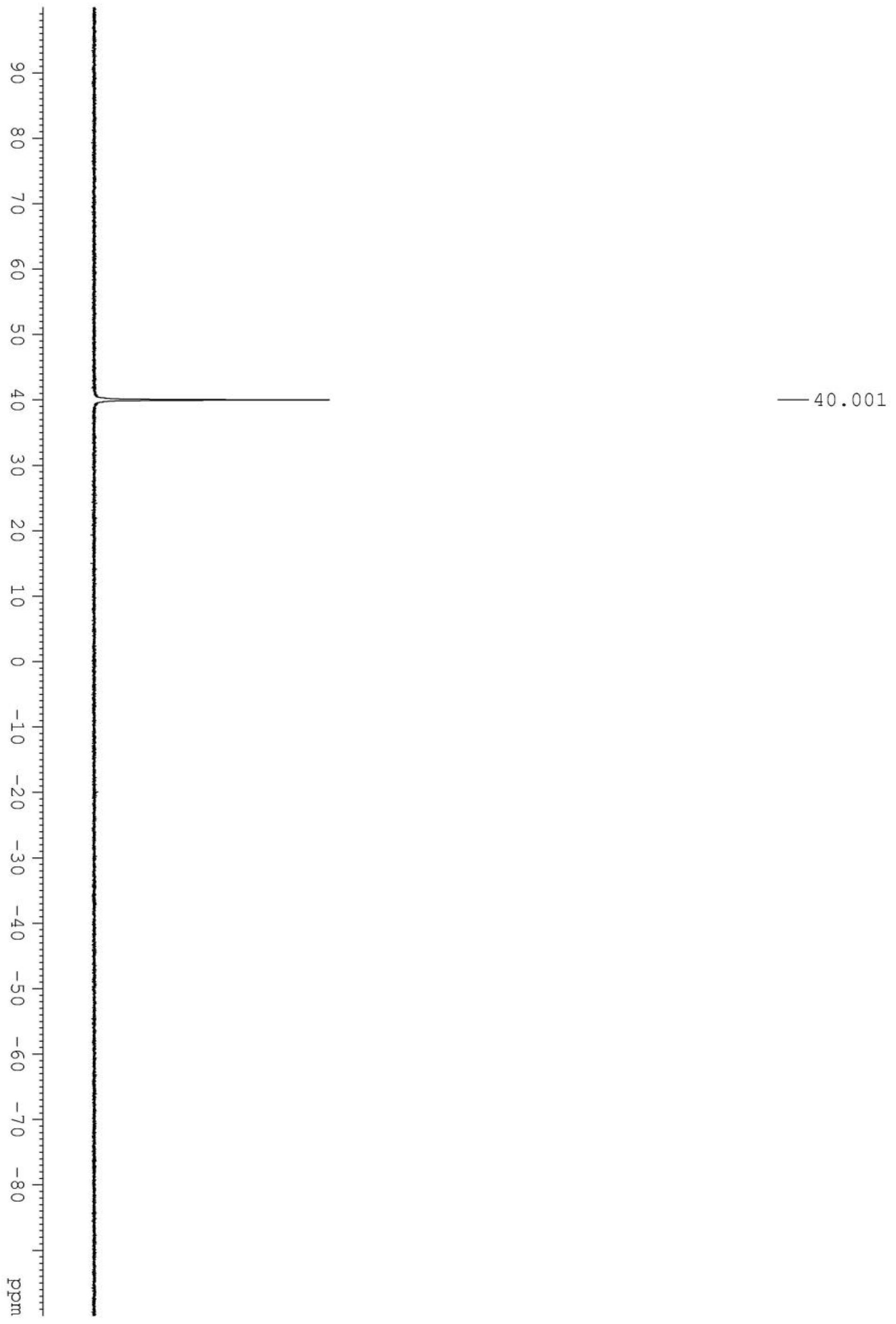
40.001
90
80
70
60
50
40
30
20
10
0
-10
-20
-30
-40
-50
-60
-70
-80
ppm

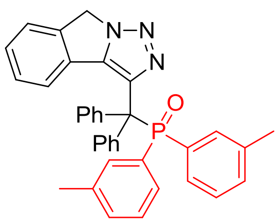
N–N
N
Ph
Ph
P
O

3q

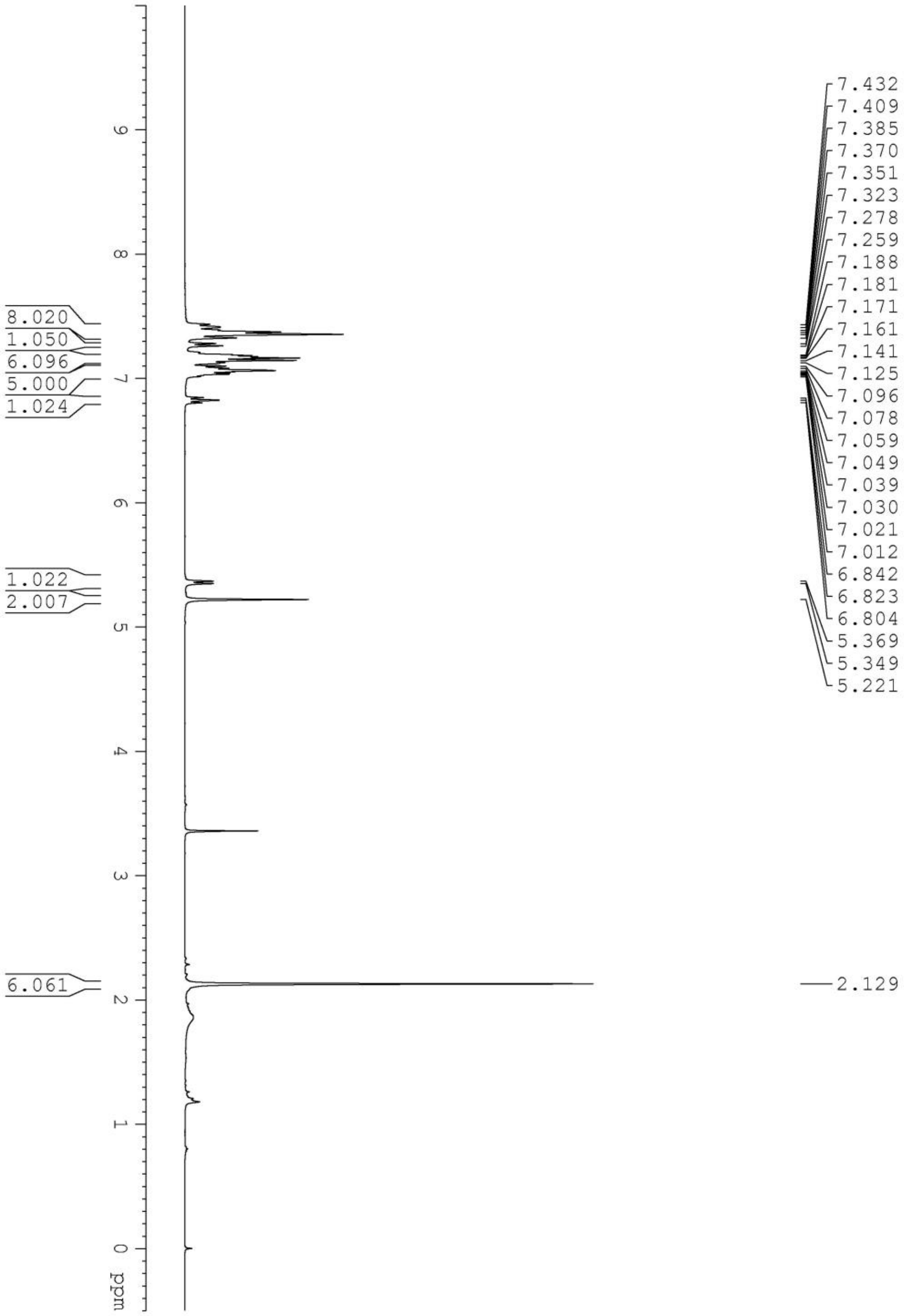
8.020
1.050
6.096
5.000
1.024
1.022
2.007
6.061
7.432
7.409
7.385
7.370
7.351
7.323
7.278
7.259
7.188
7.181
7.171
7.161
7.141
7.125
7.096
7.078
7.059
7.049
7.039
7.030
7.021
7.012
6.842
6.823
6.804
5.369
5.349
5.221
2.129
ppm

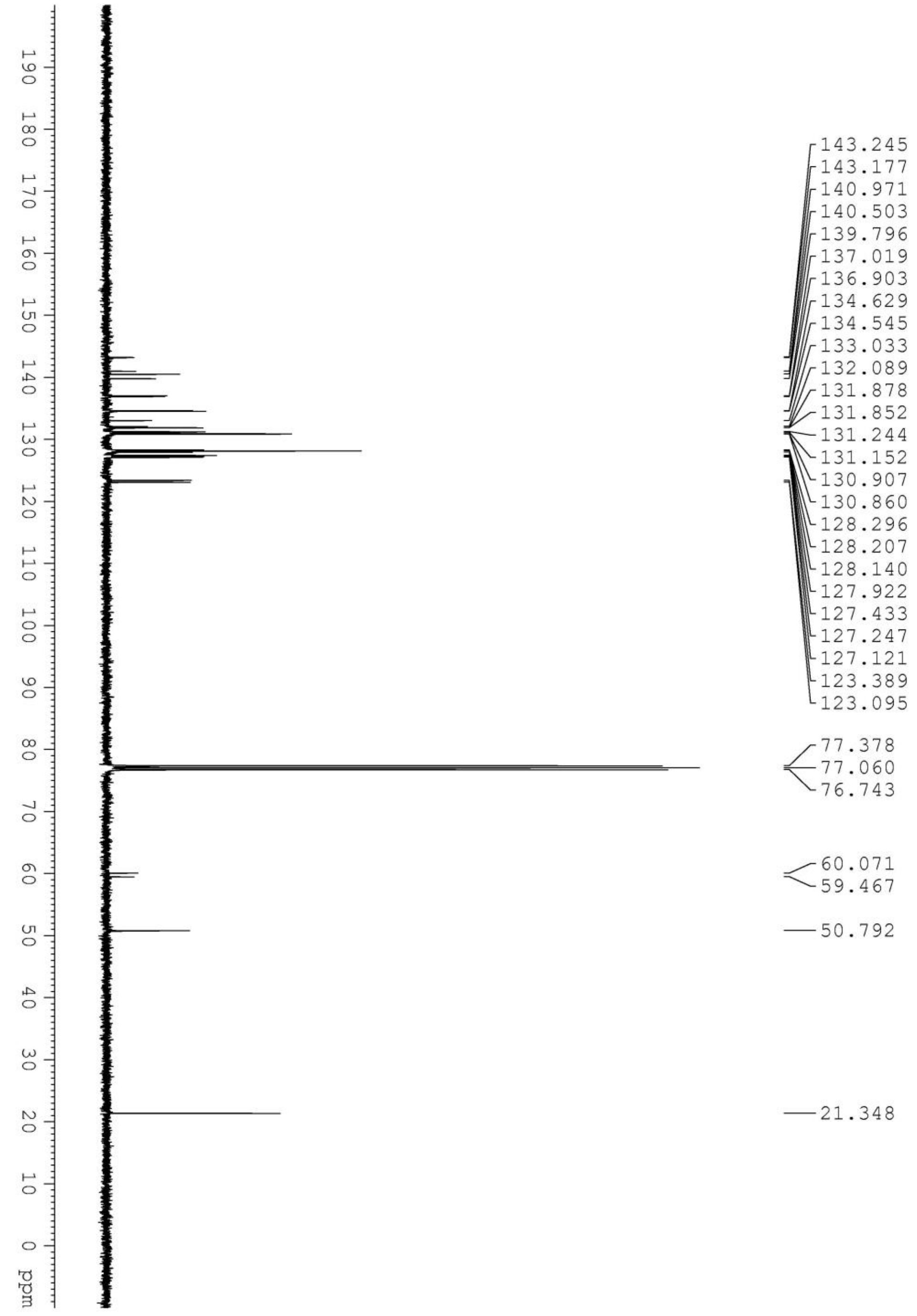

143.245
143.177
140.971
140.503
139.796
137.019
136.903
134.629
134.545
133.033
132.089
131.878
131.852
131.244
131.152
130.907
130.860
128.296
128.207
128.140
127.922
127.433
127.247
127.121
123.389
123.095
77.378
77.060
76.743
60.071
59.467
50.792
21.348
190
180
170
160
150
140
130
120
110
100
90
80
70
60
50
40
30
20
10
0
ppm

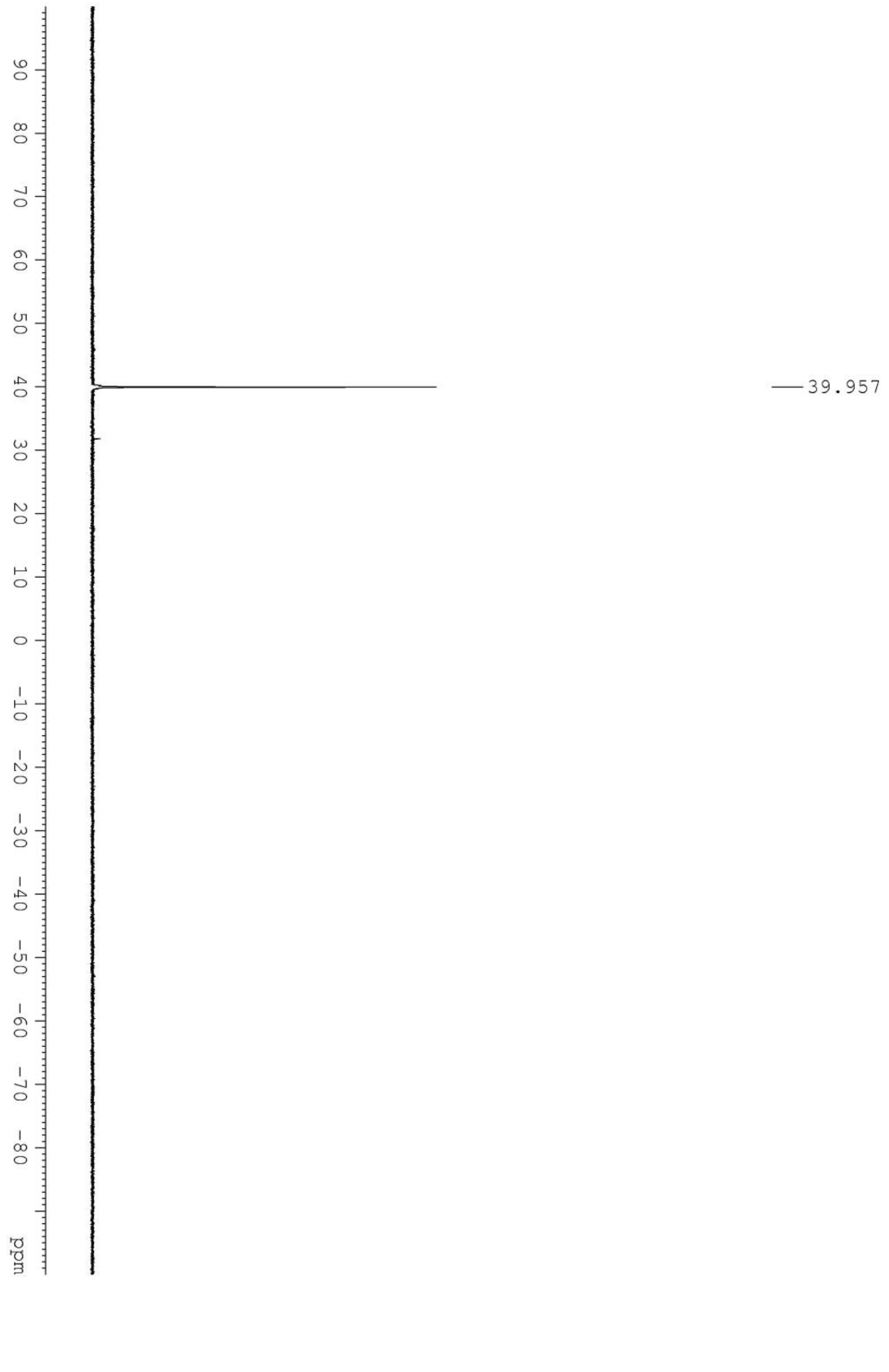

39.957
90
80
70
60
50
40
30
20
10
0
-10
-20
-30
-40
-50
-60
-70
-80
ppm

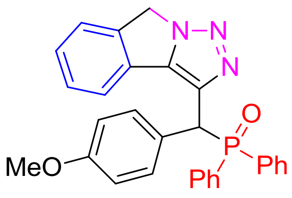
MeO
Ph
Ph
O
P
N
N
N

3s

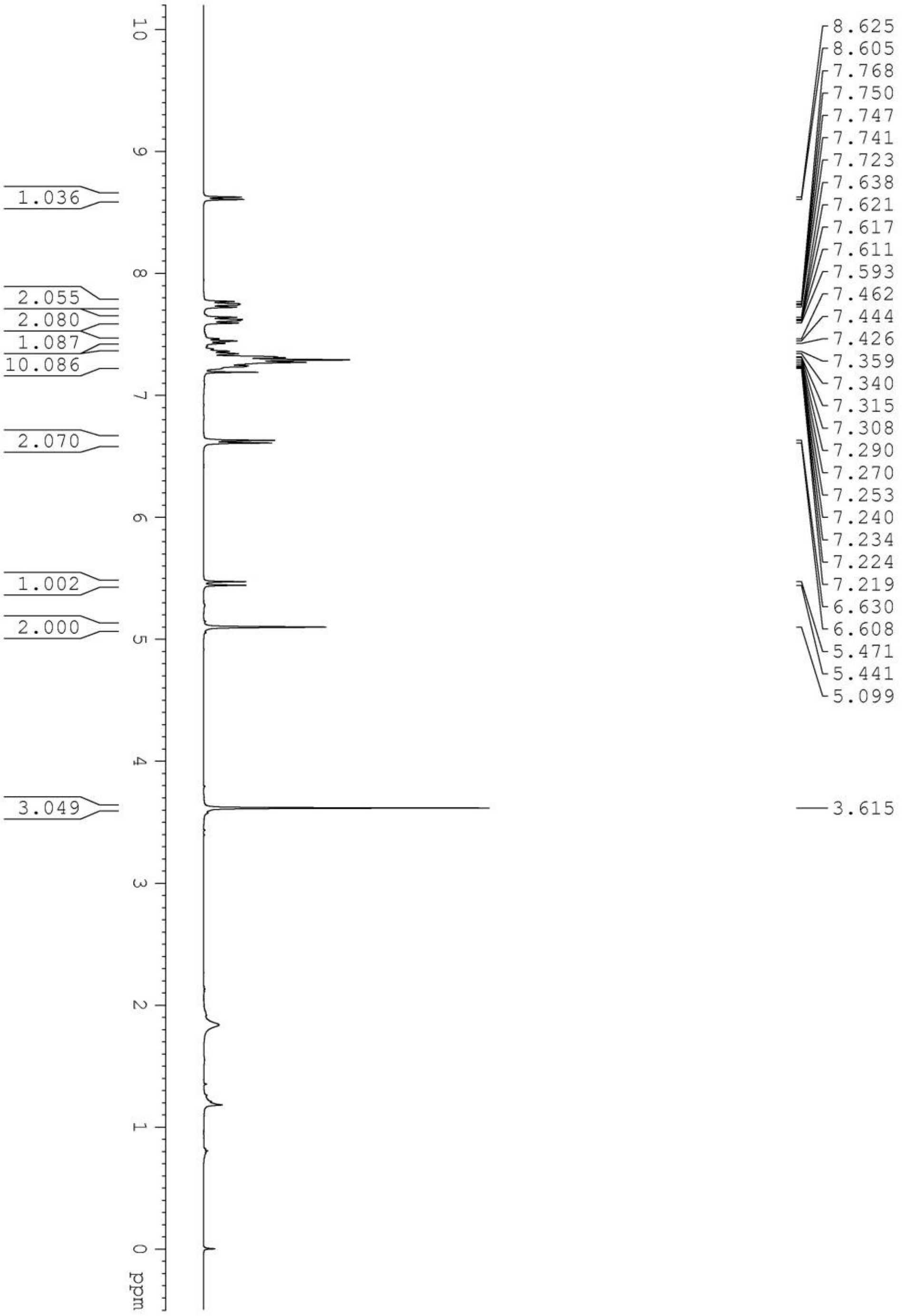
8.625
8.605
7.768
7.750
7.747
7.741
7.723
7.638
7.621
7.617
7.611
7.593
7.462
7.444
7.426
7.359
7.340
7.315
7.308
7.290
7.270
7.253
7.240
7.234
7.224
7.219
6.630
6.608
5.471
5.441
5.099
3.615
1.036
2.055
2.080
1.087
10.086
2.070
1.002
2.000
3.049
ppm

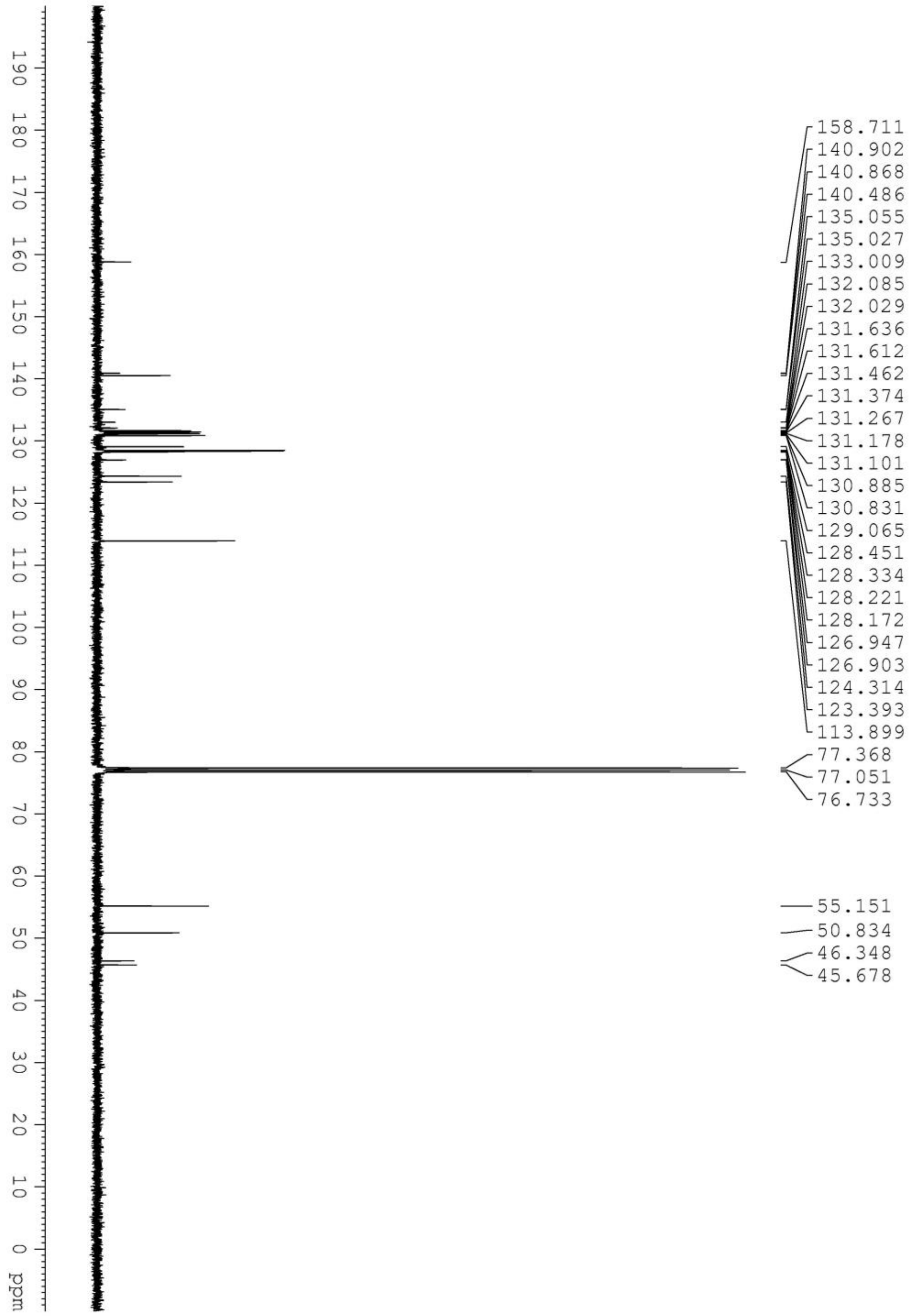
158.711
140.902
140.868
140.486
135.055
135.027
133.009
132.085
132.029
131.636
131.612
131.462
131.374
131.267
131.178
131.101
130.885
130.831
129.065
128.451
128.334
128.221
128.172
126.947
126.903
124.314
123.393
113.899
77.368
77.051
76.733
55.151
50.834
46.348
45.678
ppm

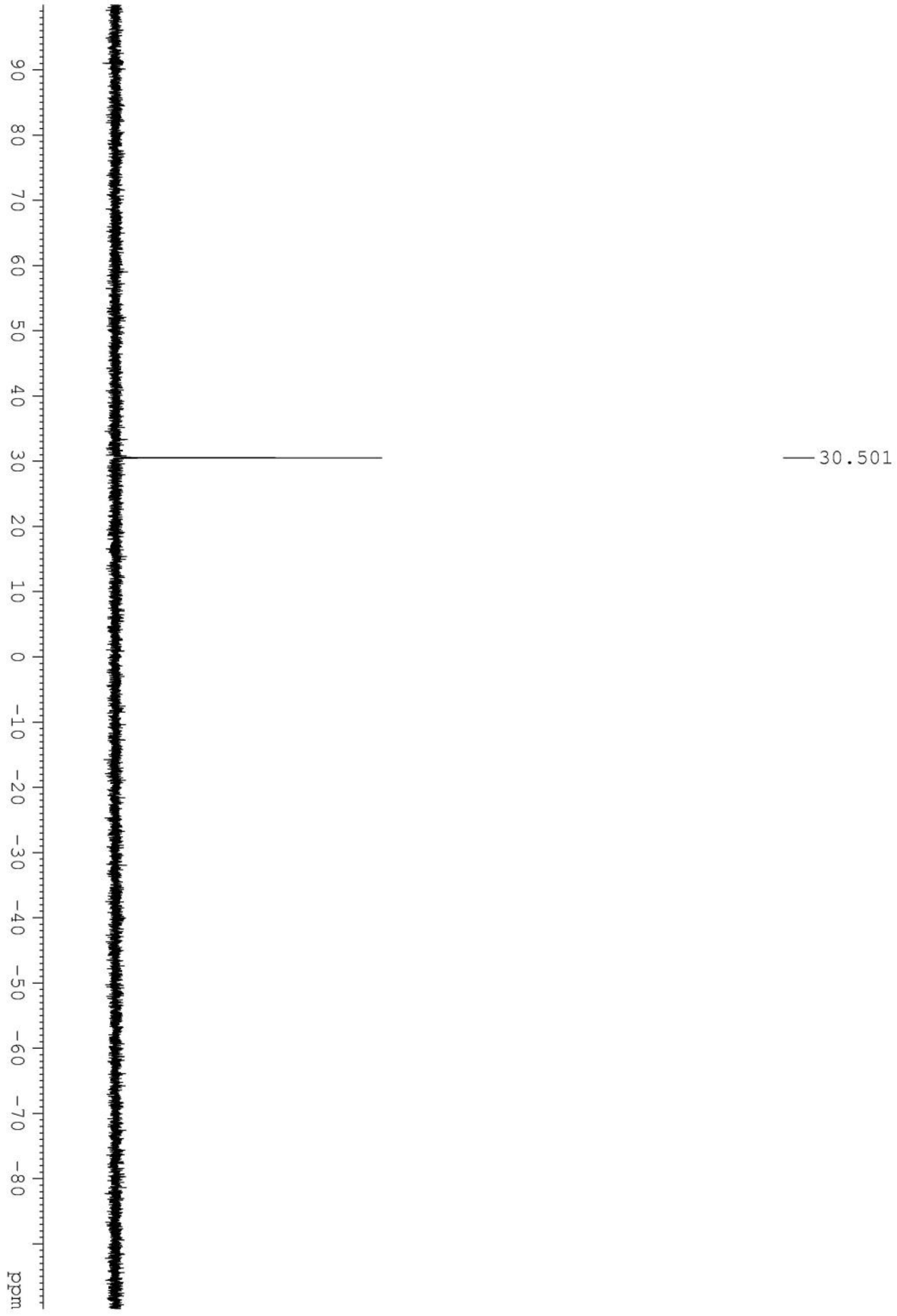
30.501
90 80 70 60 50 40 30 20 10 0 -10 -20 -30 -40 -50 -60 -70 -80
ppm

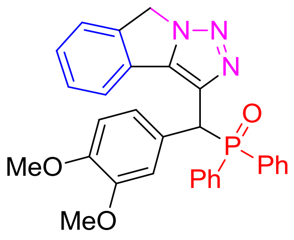

N
N
N
O
P
Ph
Ph
MeO
MeO

3t

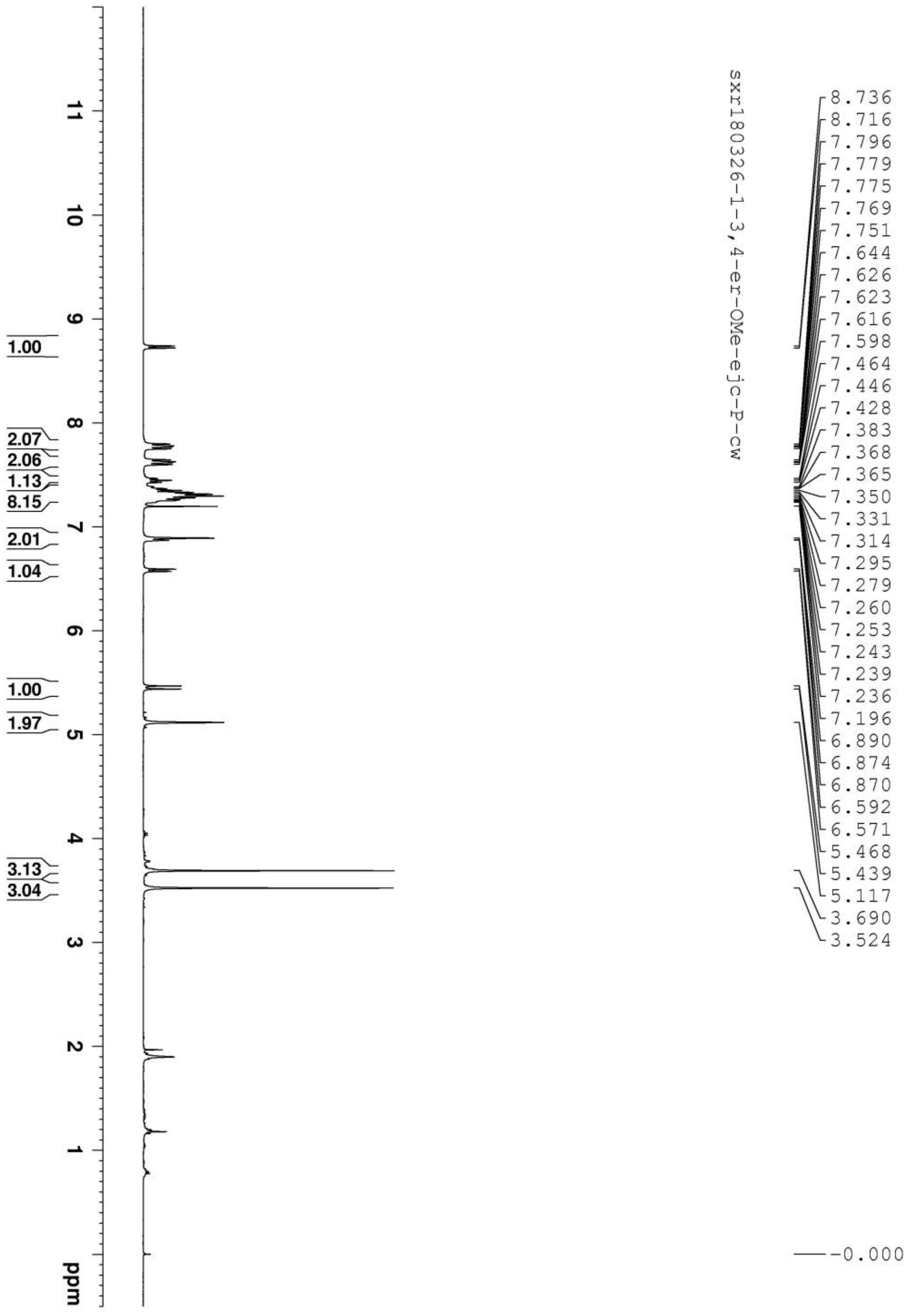

sxr180326-1-3,4-er-OMe-ejc-P-cw
8.736
8.716
7.796
7.779
7.775
7.769
7.751
7.644
7.626
7.623
7.616
7.598
7.464
7.446
7.428
7.383
7.368
7.365
7.350
7.331
7.314
7.295
7.279
7.260
7.253
7.243
7.239
7.236
7.196
6.890
6.874
6.870
6.592
6.571
5.468
5.439
5.117
3.690
3.524
-0.000
1.00
2.07
2.06
1.13
8.15
2.01
1.04
1.00
1.97
3.13
3.04
11
10
9
8
7
6
5
4
3
2
1
ppm

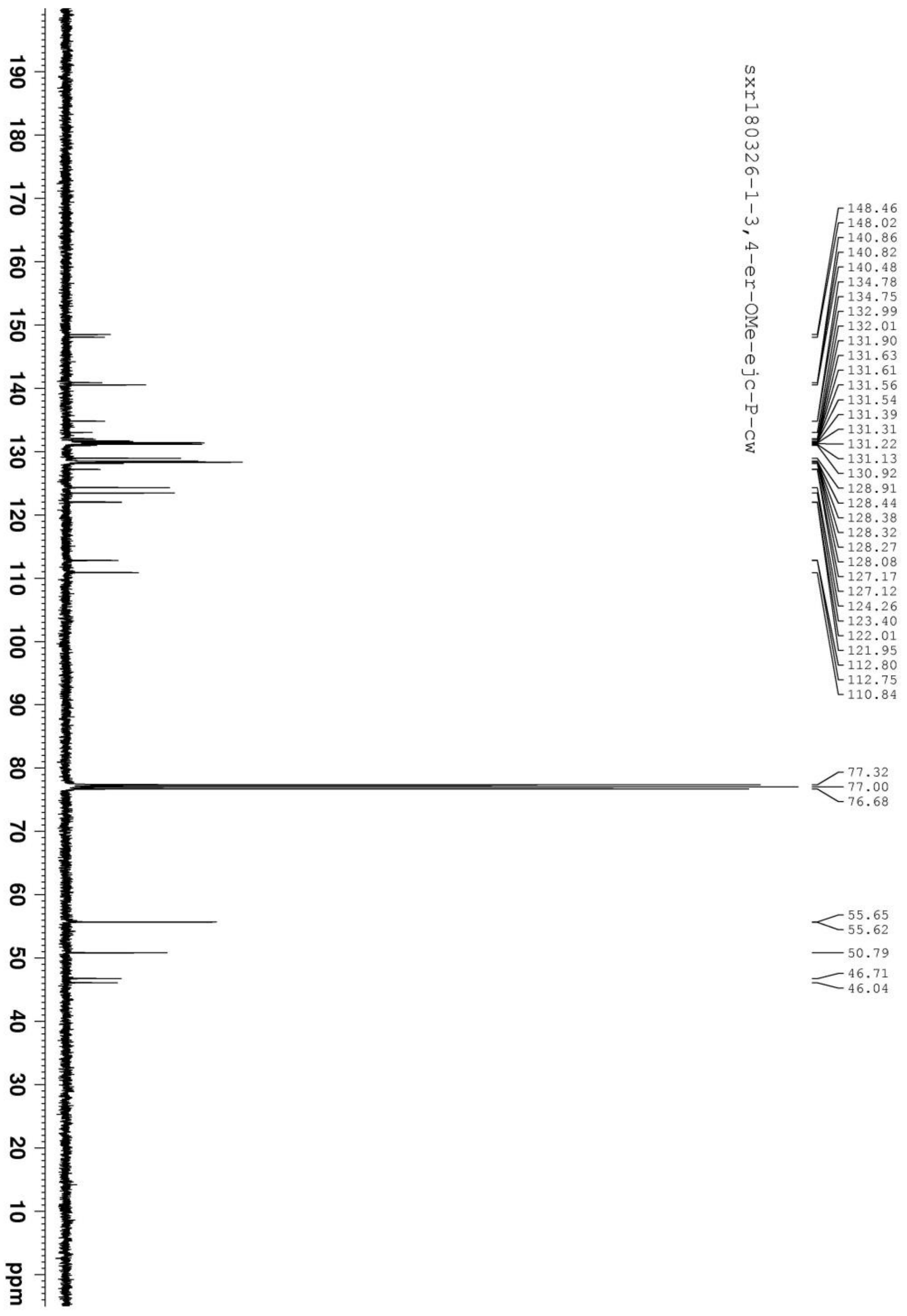
sxr180326-1-3,4-er-OMe-ejc-P-cw
148.46
148.02
140.86
140.82
140.48
134.78
134.75
132.99
132.01
131.90
131.63
131.61
131.56
131.54
131.39
131.31
131.22
131.13
130.92
128.91
128.44
128.38
128.32
128.27
128.08
127.17
127.12
124.26
123.40
122.01
121.95
112.80
112.75
110.84
77.32
77.00
76.68
55.65
55.62
50.79
46.71
46.04
190
180
170
160
150
140
130
120
110
100
90
80
70
60
50
40
30
20
10
ppm

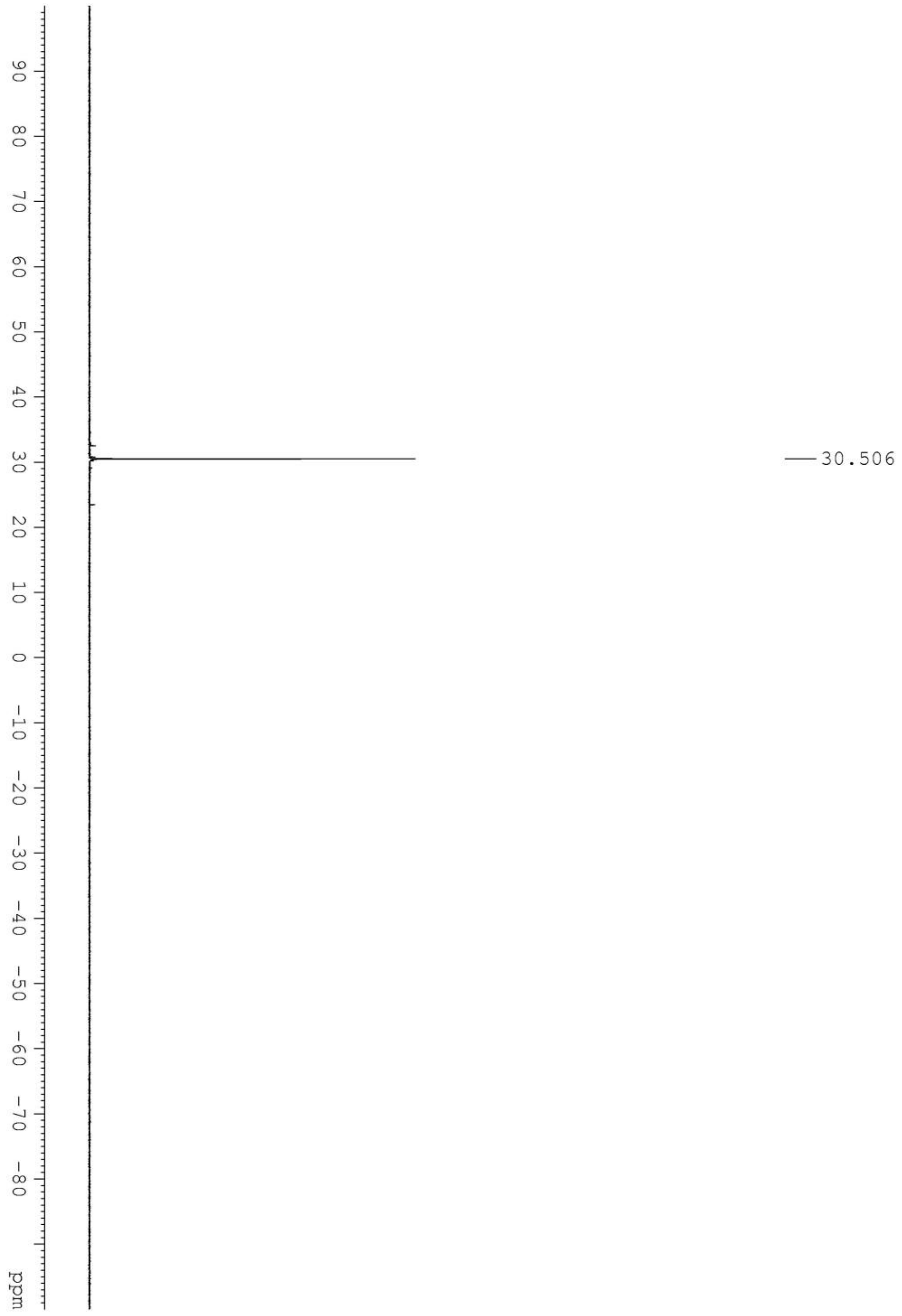
30.506
90
80
70
60
50
40
30
20
10
0
-10
-20
-30
-40
-50
-60
-70
-80
ppm
